# A patient-centric paradigm and tool for clinical research: the DOOR is open

**DOI:** 10.1128/aac.01478-25

**Published:** 2025-11-24

**Authors:** Toshimitsu Hamasaki, Yijie He, Qihang Wu, Jessica Howard-Anderson, Helen W. Boucher, Sarah B. Doernberg, Thomas L. Holland, John H. Powers, Jing Wang, Guoqing Diao, David van Duin, Vance G. Fowler, Henry F. Chambers, Scott R. Evans

**Affiliations:** 1 The Biostatistics Center, Milken Institute School of Public Health, The George Washington University 8367, Washington, DC, USA; 2 Department of Biostatistics and Bioinformatics, Milken Institute School of Public Health, George Washington University 50430, Washington, DC, USA; 3 Department of Medicine, Division of Infectious Diseases, Emory University School of Medicine 12239, Atlanta, Georgia, USA; 4 Tufts University School of Medicine and Tufts Medicine 12261, Boston, Massachusetts, USA; 5 Department of Medicine, Division of Infectious Diseases, University of California San Francisco 166668, San Francisco, California, USA; 6 Department of Medicine, Duke University Medical Center 609772, Durham, North Carolina, USA; 7 Duke Clinical Research Institute 169142, Durham, North Carolina, USA; 8 Department of Medicine, George Washington University School of Medicine, Washington, DC, USA; 9 Clinical Monitoring Research Program Directorate, Frederick National Laboratory for Cancer Research, Frederick, Maryland, USA; 10 Division of Infectious Diseases, School of Medicine, University of North Carolina Chapel Hill 2331, Chapel Hill, North Carolina, USA; Houston Methodist Hospital and Weill Cornell Medical College, Houston, Texas, USA

**Keywords:** benefit:risk, DOOR, DOOR probability, partial credit, patient-centric, pragmatic trial

## Abstract

Randomized clinical trials are the gold standard for evaluating the benefits and harms of interventions and yet may not provide the evidence needed to inform medical decision-making, an ultimate goal for clinical research. Commonly used design and analysis approaches are often not suited to answer the most important questions to inform clinical practice, specifically how do resulting patient experiences, when comprehensively considering benefits and harms, compare between therapeutic alternatives? The standard approach of siloed analysis of one outcome at a time: (i) does not incorporate associations between multiple outcomes; (ii) does not recognize the cumulative nature of multiple outcomes in individual patients or recognize important gradations of global patient response; (iii) suffers from competing risk complexities during interpretation of individual outcomes; (iv) provides for ambiguous generalizability with respect to benefit:risk since efficacy and safety analyses are often conducted on different populations. Evaluation of treatment effect heterogeneity to identify subgroups for treatment or avoidance of treatment is typically evaluated based on a single efficacy or safety endpoint and rarely evaluated based on the overall benefit:risk. Methods that quantify and compare the patient experience are needed. The desirability of outcome ranking (DOOR) is a paradigm for the design, monitoring, analysis, interpretation, and reporting of clinical trials and other research studies based on patient-centric benefit:risk evaluation, developed to address these issues and advance clinical trial science. Aligning the clinical research strategy with the relevant question for clinical practice will enhance research applicability. Careful design and comprehensive analyses are critical for DOOR paradigm application. We provide a recommended statistical analysis plan for research studies implementing DOOR, describe its elements, and illustrate analysis application using examples. A freely available online tool for the recommended analyses and the design of studies implementing the DOOR paradigm is provided.

## INTRODUCTION

### The motivating question

The ultimate goal of clinical research is to inform clinical decision-making. The most important “real world” question for which answers aid clinical decision-making is: how do resulting patient experiences, when comprehensively considering benefits and harms, compare between therapeutic alternatives?

### Limitations of siloed analyses

Randomized clinical trials are the gold standard for evaluating the benefits and harms of interventions but often fail to provide the necessary evidence to inform medical decision-making (1). Typical analyses involve siloed intervention comparisons for each efficacy and safety outcome. Estimated outcome-specific effects are potentially combined in benefit:risk analyses.

However, traditional siloed analyses of efficacy and safety, or aggregation of such, do not tell the full story of the patient’s experience. We suppose 100 patients are treated with an intervention, with 50 (50%) resulting in a positive efficacy outcome. We suppose further that 50 (50%) have an equally important safety outcome. Now consider two possible scenarios. One scenario is that the 50 patients with efficacy are the same as the 50 that had a safety outcome, resulting in zero patients with benefit without negating toxicity, evidence of a worthless treatment. Consider another possibility that the 50 patients with efficacy are distinct from the 50 who had a safety outcome, resulting in 50 patients with benefit without negating toxicity, evidence of a good treatment if one can find the right patients. Siloed analyses cannot tell the difference between scenarios, ineffectively describing the patient experiences (see Section 1.1 of the Supplemental material for further explanations).

Generally, siloed analyses: (i) do not incorporate associations between outcomes; (ii) do not recognize the cumulative nature of various outcomes on individuals or important gradations of the overall patient response; (iii) suffer from competing risk challenges when interpreting outcome-specific results; (iv) since efficacy and safety analyses are conducted on different analysis populations, the population to which these analyses generalize and the target estimand is unclear. Treatment effect heterogeneity is typically evaluated based on a single endpoint and rarely evaluated based on benefit:risk. See the Rational Section of the Supplemental material for further explanation of limitations.

## DESIRABILITY OF OUTCOME RANKING (DOOR)

The desirability of outcome ranking (DOOR) is a patient-centric paradigm for the design, data monitoring, analysis, interpretation, and reporting of clinical trials and other research studies based on benefit:risk evaluation (2–6). Patient-centric in this context means that the DOOR paradigm uses outcomes to analyze patients rather than patients to analyze outcomes by comparing the experiences of trial participants in different treatment arms by the desirability of the overall patient response, with the aim of directly addressing the important question for clinical decision-making, and addressing the limitations described above.

### DOOR outcome

The first step in implementing DOOR is to define an ordinal DOOR outcome representing the “patient story/experience,” a global patient-centric summary of the benefits and harms for the patient. This task requires evaluating the tradeoffs among outcomes and the cumulative nature of benefits and harms at the patient level.

Clinical importance considerations drive DOOR outcome development. The experience or status of the trial participant is summarized at a patient level, similar to the manner in which a clinician may summarize the experience or status of a patient in clinical practice. It is recommended that a DOOR outcome is constructed using component outcomes that represent important measurements of how patients feel, function, or survive. Component outcomes are selected based on the clinical importance with a goal to define a fair representation of the overall patient response. Selective inclusion or exclusion of component outcomes to favor a specific treatment is discouraged. Guidelines for construction of the DOOR outcome are as follows: (i) define gradations of patient response to ensure recognition of responses that are importantly different clinically; (ii) ensure defined gradations are clinically important to assure that nonmeaningful effects are not overemphasized; (iii) simplicity.

Binary outcomes are commonly utilized in infectious disease studies. There is often reluctance to develop ordinal outcomes due to uncertainties regarding how to define the response, i.e., how to layer or analyze the outcome. Yet avoidance of intentional layering or grading through the use of a binary endpoint does not prevent it, but instead results in incidental all-or-nothing grading, which may not recognize nuanced but clinically meaningful levels of response. We consider mortality, a noncomposite binary endpoint. Its noncomposite nature does not imply homogeneity within its dichotomized levels. During analyses, the response of a patient who survives without complications is classified equivalently to the response of a patient who survives but is hospitalized with organ support via dialysis or mechanical ventilation or both. Other trials utilize an endpoint of “clinical failure,” a binary composite of mortality and other criteria indicating a poor response. During analyses, the response of a patient who dies is classified equivalently to the response of a patient who survives but fails based on a non-fatal criterion. Though possibly unintentionally or unwittingly, all-or-nothing grading has been implemented. More meaningful and calculated layering of patient-centric responses can be considered.

For example, a trial evaluating monoclonal antibodies to prevent *S. aureus* ventilator-associated pneumonia in intubated and ventilated ICU patients who are positive for *S. aureus* colonization of the lower respiratory tract may consider a clinical failure composite endpoint consisting of mortality, ventilator use, or unresolved symptoms. The binary nature of the endpoint equates mortality with being alive without symptom resolution or on a ventilator. A 4-level DOOR outcome: (a) alive with resolved symptoms and without ventilator use (most desirable); (b) alive with either unresolved symptoms or ventilator use; (c) alive with both unresolved symptoms and ventilator use; and (d) death (least desirable), distinguishes important gradations of patient response, allowing for recognition of more subtle though important treatment effects should they exist.

DOOR outcomes can help address competing risk challenges. Competing risks typically arise when the plan is to evaluate clinical success/failure at a test-of-cure visit, but mortality occurs prior to the test-of-cure visit. Mortality could be treated as clinical failure along with other non-fatal criteria for failure, and analyses of the composite binary endpoint can ensue. However, this approach equates mortality with non-fatal clinical failure criteria. Alternatively, the competing risk of death could be incorporated but in a manner that distinguishes between mortality and survival with clinical failure, resulting in, e.g., a 3-level DOOR outcome: (a) alive with clinical success; (b) alive with clinical failure; (c) death. For example, a trial evaluating fecal microbiota transplantation (FMT) in participants colonized with multidrug-resistant organisms (MDRO) may investigate whether FMT reduces intestinal MDRO colonization. Mortality is a competing risk challenging the interpretation of a colonization reduction outcome. A composite binary outcome of colonization or mortality would not distinguish between them. A 3-level DOOR outcome: (a) alive with reduced MDRO colonization (most desirable); (b) alive without reduced MDRO colonization; (c) death addresses the competing risk challenge and distinguishes mortality from reduced MDRO colonization.

DOOR outcomes are ideally evidence-based and consensus-informed with stakeholder engagement to maximize objectivity, transparency, and utility for clinical practice. Conjoint analyses and surveys of expert clinicians and patients can be used to inform DOOR outcome construction (7). Delphi analyses have been used to develop a DOOR outcome in periprosthetic joint infection (8). The Antibacterial Resistance Leadership Group (ARLG), funded by the National Institute of Allergy and Infectious Diseases (NIAID), hosts an Innovation Working Group that works with regulators to develop DOOR outcomes for each of the four most common indications for regulatory approval of antibacterial compounds (9) including complicated intra-abdominal infections (cIAI) based on an FDA ORISE fellowship (10), complicated urinary tract infection (cUTI) (11), hospital-acquired bacterial pneumonia and ventilator-associated bacterial pneumonia (HABP/VABP) (12, 13), and acute bacterial skin and skin structure infection (ABSSSI) (14).

For most DOOR outcomes, mortality defines the worst category. If there is a non-fatal event that is clearly worse than other non-fatal events, then that event may define the next worst category, and so on. A survey of clinicians conducted by the ARLG indicated that non-fatal absence of clinical response (a typical efficacy variable), non-fatal infectious complications, and non-fatal SAEs had comparable importance, but that having more of these deleterious events was worse than fewer (7). DOOR outcomes were developed based on these findings. There may be situations where the events are unequally important. For example, regulators inquired about whether efficacy or safety events could be prioritized. Versions of DOOR outcomes further prioritizing efficacy or safety events were easily constructed.

A common question is whether it is advantageous to have more or fewer categories in the DOOR outcome when considering statistical power. Binary outcomes are 2-level DOOR outcomes. Allowing for more categories: (i) allows for recognition of finer gradations of patient responses if they are present, increasing power; and (ii) increases variance, decreasing power. The net effect of including more categories on power depends upon the nature of the effects and how the DOOR outcome is defined. In our experience, 3–6 levels work well balancing simplicity with the goal of defining clinically meaningful gradations of the patient response. Once the DOOR outcome is defined, freely available online tools for sample size and power evaluation are available for clinical trial design (15) (https://methods.bsc.gwu.edu/web/methods/door-sample-size).

### Analyses

There are two complementary approaches to DOOR analyses, a rank-based and a grade-based approach. Analyses are conducted in parallel to maximize the understanding of treatment effects (6).

The rank-based approach utilizes the concept of pairwise comparisons of the DOOR outcome responses between two intervention groups and summarizes the intervention contrast by estimating the “DOOR probability,” i.e., the probability of a more desirable result [adjusted for tied desirability] for a trial participant assigned to one intervention relative to a trial participant assigned to the other intervention. Although unfamiliar and distinct from commonly used metrics such as the difference in means or proportions, or a hazard ratio, the DOOR probability can have intuitive appeal. During clinical decision-making, it is helpful to know the probability that a patient will have a more desirable overall outcome, i.e., DOOR outcome, while on one intervention relative to a therapeutic alternative. When the two DOOR outcome distributions are identical, then the DOOR probability is 50%, resembling flipping a fair coin. However, a DOOR probability of 50% does not necessarily imply equivalent distributions.

The DOOR probability is simple to estimate. For example, if one intervention group has $n_{1}$ patients and the other has $n_{2}$ patients, then there are $n_{1}\times n_{2}$ possible pairwise comparisons among patient responses. When comparing a specific patient’s DOOR outcome results from one group to a patient from the other group, a more desirable, less desirable, or equally desirable (tie) result is observed. The DOOR probability is estimated by the Wilcoxon-Mann-Whitney (WMW) statistic, i.e.,

DOOR probability = ([# of more desirables] + 1/2 [# of ties])/(${n_{1}\times n}_{2}$)

DOOR probabilities can be estimated for binary, ordinal, continuous, or censored event-time endpoints. Confidence intervals (CI) for the DOOR probability are estimated using Halperin et al. (16) and hypothesis testing, e.g., whether the DOOR probability is greater than 50% can be estimated using the WMW test.

The rank-based approach does not make assumptions about the perceived spacing between the DOOR outcome categories, basing analyses entirely on ranks. During analyses, researchers may wish to directly incorporate perspectives on the desirability of the DOOR outcome categories via deliberate grading. This can alleviate concerns that a small decrement in a very important outcome component could be offset by a large advantage in an outcome component of lesser importance despite appropriate prioritization and ranking.

Partial credit analysis, the grade-based approach (3), is designed for this purpose. Partial credit analysis involves a “grading key” for the levels of the DOOR outcome similar to an academic test, i.e., from 0 to 100%. If the patient experiences the most desirable overall outcome, generally benefits without harms, then they receive a grade of 100%. If the patient has the least desirable result, i.e., death, then they receive a grade of 0. Partial credit, a grade between 0 and 100%, is given for intermediate DOOR outcome categories. Partial credit may be informed from the following: (i) patients using, e.g., patient-reported outcome measures (PROMs) or (ii) expert clinicians via surveys. Treatment comparisons are made by comparing means based on partial credit grades, between treatments. The advantages of the partial credit scoring approach are that it strategically grades the DOOR categories in a calculated and transparent way and it has an intuitive interpretation given the 100-point scale. Partial credit scoring can be prespecified during clinical trial design for transparency and serve as a foundation for statistical error control. During analyses, the estimated treatment contrast can be displayed as partial credit assignment varies, allowing for the following: (i) robustness analyses; (ii) researchers, clinicians, and patients the freedom to evaluate the treatment effects based on personalized grading-key perspectives regarding the desirability of the DOOR categories; (iii) identification of the “tipping boundary” that distinguishes grading keys that favor one therapy vs. an alternative therapy, valuable for comparative effectiveness studies.

### Guiding principles, recommended statistical analysis plan, and online application

DOOR analyses are shaped by guiding principles for maximizing replicability, robustness, and rigor (Table 1). The recommended statistical analysis plan (SAP) for DOOR and a freely available online application (app; https://methods.bsc.gwu.edu/) implementing the recommended analyses are available. A summary of app output based on the recommended SAP for DOOR is displayed in Table 1. Resulting tables and figures can be saved for manuscripts, presentations, and reports.

**TABLE 1:** DOOR analyses: Guiding principles, recommended statistical analysis plan, and online DOOR application

Guiding principles for maximizing pragmatism, robustness, replicability, objectivity, and transparency
Patient-Centricity and Pragmatism Preservation
Analyze the patient story/journey; recognize the cumulative nature of effects
Distinguish important gradations of the patient’s response
Best Practices
Composite endpoints (integrate the presentation of component outcome analyses)
Multi-outcome/benefit:risk analyses (analyses based on the absolute risk scale providing a common scale for simultaneous interpretation of multiple outcomes; avoid relative risk/ratio scales)
Ordinal outcomes (cumulative analyses)
Statistical Integrity and Discipline
Robustness: avoid/minimize reliance upon assumptions, e.g., common odds, distribution of treatment effects, specification of model form, and for analysis validity
Incorporate competing risks into patient-centric outcomes
Intention-to-treat principle with a full analysis set for all outcomes (clarity of generalizability; established applicability at the time of treatment initiation)
Objectivity: free from subjective investigator-specific beliefs
Defined population parameters and estimands
Theoretical foundation for the confirmatory evidence standard Unbiased estimates of treatment effects Correct coverage probability for confidence interval estimation Error control in hypothesis testing Avoid black-box computation
Incorporation of ties into rank-based statistics utilizing pairwise comparisons
Implementation of rank-based and grade-based analyses of treatment contrast
Evaluation of the robustness of grade-based analyses

Recommended statistical analysis plan and associated DOOR app output
Statistical Analysis Plan
Table: DOOR Outcome Distribution by Treatment Note: The table includes a standardized expected gain/loss summary. The expected DOOR distribution for the control therapy standardized per a specified number of user-defined assigned patients is displayed. The summary provides the number of patients that would be gained or lost in each DOOR level and the cumulative gain/loss if treated with the investigational therapy relative to the control.
Table: Distribution of Component Outcomes by Treatment
Figure: Bar Chart of DOOR Outcome Distribution by Treatment
Figure: Bar Chart of Component Outcomes Distributions by Treatment
Figure: Forest Plot of Confidence Interval Estimates for the DOOR Probability for the DOOR Outcome and Respective Components Note: The DOOR outcome is a composite. A fundamental tenet of analyses of composites is incorporation of the analysis of each of the components to ensure a thorough understanding of the manifestation of effects (17), e.g., whether effects on components go in similar or opposing directions, or if there are specific components driving the observed effect on the composite. Comprehensive analyses include estimation of the DOOR probability for individual components such as mortality. The DOOR probability metric is an absolute metric that provides for a common scale for all outcomes, allowing for simultaneous interpretation of multiple outcomes, providing an advantage over relative measures that do not have a common scale due to different baseline rates (18). Resulting analyses includes confidence interval estimates of a more desirable result with respect to the DOOR outcome and each of its components. Note: The app includes options to prioritize efficacy or safety components when estimating the DOOR probability by using pairwise comparisons of surviving patients with events.
Figure: Forest Plot of the Confidence Interval Estimates for the Cumulative DOOR Probability Based on Sequential Dichotomization of the DOOR Outcome
Table: Partial Credit Analyses Summary for Selected Grading Keys
Figure: Plot of the Difference in Means vs. DOOR Probability Based upon Selected Grading Keys The plot includes vertical and horizontal lines for each bivariate estimate displaying grading key-specific confidence interval estimates for the difference in means and the DOOR probability.
Figure: Anthology of Patient Stories Clarification: The plot displays the percentage of patients experiencing each combination of component outcomes by treatment.
Figure: For DOOR outcomes with three levels, a plot of the difference in means and associated point-wise confidence band, as the partial credit assigned to the middle DOOR outcome category varies from 0 to 100%, is provided. For DOOR outcomes with four levels, a contour plot of the difference in means as the partial credit assigned to the middle two DOOR outcome categories varies from 0 to 100%, is provided. Color provides information regarding statistical significance, e.g., areas shaded in green identify grading keys that result in *P*-values less than 0.05.
DOOR App
Standard and professional editions of the app are provided with supporting documentation. The standard edition reads in the DOOR outcome distribution summary table (frequencies or percentages) by treatment and the desired partial credit grading keys, producing all of the summaries in Table 1, except the Anthology of Patient Stories. There is a pull-down menu for standard DOOR outcomes, e.g., those being developed by the ARLG (10–13) or investigators can create their own DOOR outcome by choosing and labeling the number of levels and component outcomes. The app allows for the labeling of treatment groups, choice of confidence levels for confidence interval estimates, range selections on forest plots, and the selection of partial credit grading keys.
The professional edition reads in patient-level data producing all of the summaries in Table 1. It allows for application of inverse-probability of treatment weighting (IPTW) that can be used to address confounding in observational studies or missing data issues in clinical trials and allows for tie-breaker analyses that can further distinguish ranks within DOOR outcome categories.

## EXAMPLES

Application of the recommended DOOR analyses using the app is illustrated using examples. Selected components of the analyses are displayed here. The remainder of the recommended analyses is provided in the Supplemental material.

### Intravenous (IV) doripenem vs. IV levofloxacin for treatment of complicated urinary tract infections (cUTI) and pyelonephritis (DORI-05)

DORI-05 was a randomized double-blind clinical trial that compared IV doripenem to IV levofloxacin with a step-down option of oral levofloxacin in both groups after 3 days of IV therapy, for the treatment of cUTI and pyelonephritis (19). Analyses are illustrated using a DOOR outcome developed by the ARLG (11). The DOOR outcome incorporates mortality and three non-fatal deleterious events: absence of clinical response (a typical efficacy variable), non-fatal serious adverse events (SAEs), and infectious complications. The resulting 5-level DOOR outcome recognizes death as the worst outcome and distinct from other deleterious events and the cumulative nature of the non-fatal deleterious events, i.e., being alive with zero events is the most desirable patient response and alive with one of the deleterious events is the second most desirable. (Table 2).

**TABLE 2:** DOOR outcome and respective component outcomes distributions by treatment in DORI-05, ACTT-1, CRACKLE (unadjusted), and CRACKLE (adjusted)

DOOR category/components			Cum.				Cum.		Expected gain or loss^*a*^	
	N	(%)	N	(%)	N	(%)	N	(%)	Gain or loss	Cum.
DORI-05	Doripenem (DOR)				Levofloxacin (LEV)				DOR - LEV	
Alive with no events	263	70.3	263	70.3	253	67.6	253	67.6	27	27
Alive with 1 event	93	24.9	356	95.2	111	29.7	364	97.3	−48	−21
Alive with 2 events	16	4.3	372	99.5	9	2.4	373	99.7	19	−3
Alive with 3 events	1	0.3	373	99.7	1	0.3	374	100.0	0	−3
Death	1	0.3	374	100.0	0	0.0	374	100.0	3	0
Absence of clinical success	81	21.7			113	30.2			−86	
Infectious complications	23	6.1			5	1.3			48	
Non-fatal SAEs	25	6.7			14	3.7			29	
Death	1	0.3			0	0.0			3	

ACTT-1	Remdesivir (RDV)				Placebo (PLB)				RDV - PLB	
Alive with no events	433	80.0	433	80.0	382	73.3	382	73.3	67	67
Alive with 1 event	42	7.8	475	87.8	57	10.9	439	84.3	−32	35
Alive with 2 events	8	1.5	483	89.3	6	1.2	445	85.4	3	39
Death	58	10.7	541	100.0	76	14.6	521	100.0	−39	0
Hosp. with IMV/ECMO	45	8.3			55	10.6			−22	
Non-fatal SAEs	11	2.0			13	2.5			−5	
Death	58	10.7			76	14.6			−39	

CRACKLE (unadjusted)	Ceftazidime-avibactam (CAZ-AVI)				Colistin (CST)				CAZ-AVI - CST	
Discharged home	6	23.1	6	23.1	4	8.7	4	8.7	144	144
Alive in hospital or discharged not to home, no renal failure incident	17	65.4	23	88.5	25	54.3	29	63.0	110	254
Alive in hospital or discharged not to home, renal failure incident	1	3.8	24	92.3	5	10.9	34	73.9	−70	184
Hospital death	2	7.7	26	100.0	12	26.1	46	100.0	−184	0
Renal failure	1	3.8			7	15.2			−114	
Not discharged	20	76.9			42	91.3			−144	
Death	2	7.7			12	26.1			−184	

CRACKLE (adjusted)	Ceftazidime-avibactam (CAZ-AVI)				Colistin (CST)				CAZ-AVI - CST	
Discharged home		20.5		20.5		7.8		7.8	126	126
Alive in hospital or discharged not to home, no renal failure incident		65.4		85.9		55.9		63.7	95	221
Alive in hospital or discharged not to home, renal failure incident		5.1		90.9		11.3		75.0	−62	159
Hospital death		9.1		100.0		25.0		100.0	−159	0
Renal failure		5.1				14.9			-98	
Not discharged		79.5				92.2			-126	
Death		9.1				25.0			-159	

^*a*^ The expected DOOR distribution for the control intervention standardized per a specified number of assigned patients (per 1,000) is shown. The summary provides the number of patients who would be gained or lost in each category if treated with the experimental relative to the control. The cumulative gain/loss is also provided.

Table 2 displays the distribution of the DOOR outcome and respective components by treatment; 70.3% of trial participants assigned to doripenem had the most desirable result (alive with no events) vs. 67.6% for levofloxacin. One patient died in the doripenem arm vs. zero for levofloxacin. If 1,000 patients like those in the trial received doripenem in practice instead of levofloxacin, then there would comparatively be an estimated gain of 27 patients as alive with zero events, 86 fewer clinical failures, 48 more infectious complications, 29 more SAEs, and three more deaths.

**Fig 1:**
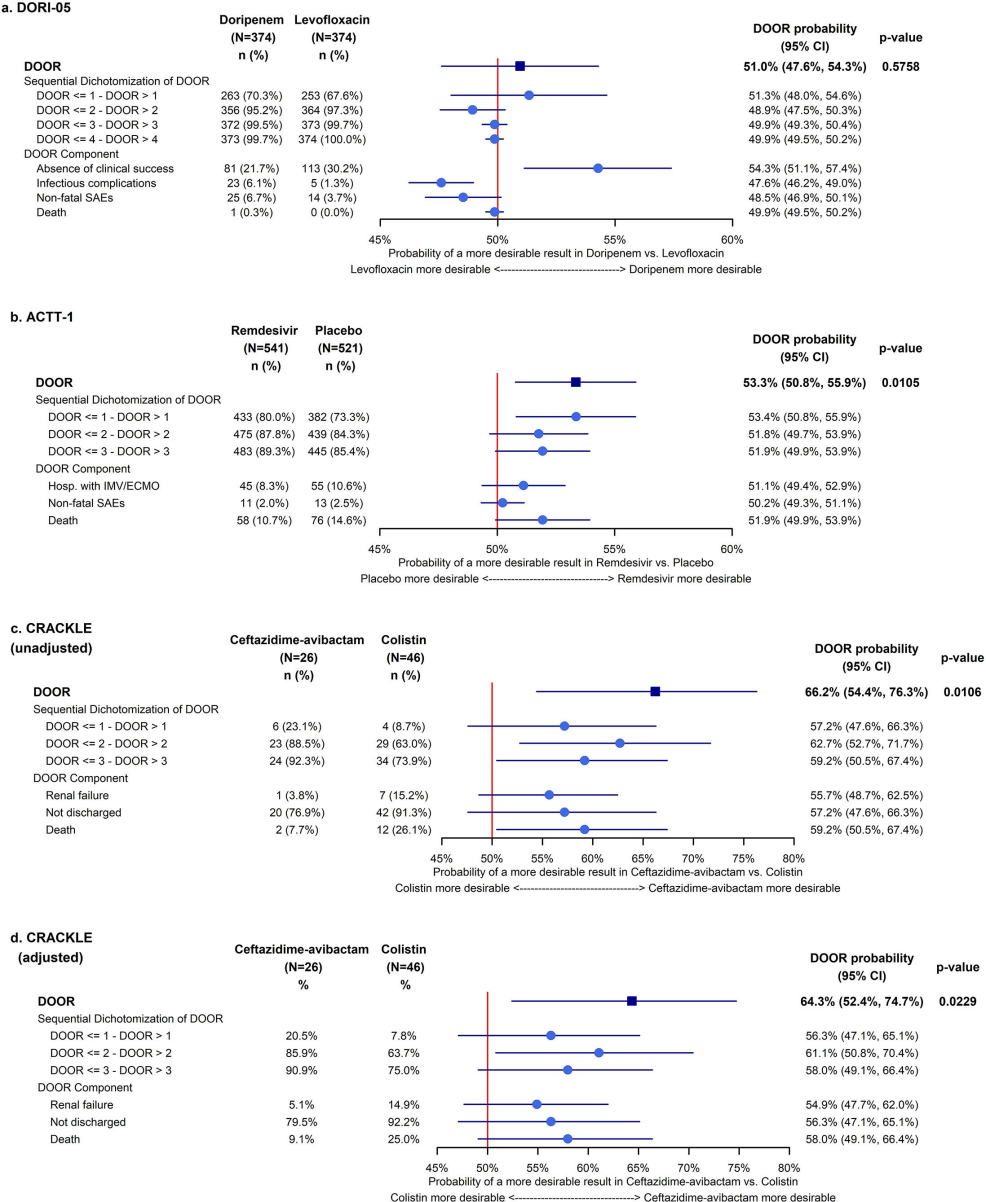
Forest plots of confidence interval estimates of the DOOR probability for the DOOR outcome and respective components in (**a**) DORI-05, (**b**) ACTT-1, (**c**) CRACKLE (unadjusted), and (**d**) CRACKLE (adjusted).

The probability of a more desirable outcome for doripenem compared to levofloxacin is 51.0% (95% CI: 47.6%, 54.3%; *P* = 0.58). CI estimates for the cumulative DOOR probability are displayed in Fig. 1a. The probability of a more desirable outcome for doripenem compared to levofloxacin with respect to the absence of the clinical response is 54.3% (95% CI: 51.1%, 57.4%) and for infectious complications is 47.6% (95% CI: 46.2%, 49.0%) (Fig. 1a).

### Adaptive COVID-19 treatment trial (ACTT-1)

The Adaptive COVID-19 Treatment Trial (ACTT-1) was a randomized, double-blind, placebo-controlled trial of IV remdesivir in adults who were hospitalized with COVID-19 and had evidence of lower respiratory tract infection. Trial participants were randomly assigned to receive remdesivir or placebo for up to 10 days (20).

We illustrate *post hoc* DOOR analyses from ACTT-1 using a DOOR outcome developed by trial investigators. The DOOR outcome incorporates mortality, and two non-fatal deleterious events: hospitalization with invasive mechanical ventilation (IMV)/extracorporeal membrane oxygenation (ECMO) and SAEs. This results in a 4-level DOOR outcome that recognizes death as the worst outcome and the cumulative nature of the non-fatal deleterious events (Table 2).

Table 2 displays the distribution of the DOOR outcome and respective components by treatment; 80.0% of trial participants assigned to remdesivir had the most desirable result (alive with no events) vs. 73.3% for placebo; 14.6% of trial participants assigned to the placebo arm had the least desirable outcome of death vs. 10.7% for remdesivir. If 1,000 patients in practice were given remdesivir instead of standard of care, then there would comparatively be an estimated gain of 67 patients as alive with zero events, 22 fewer hospitalizations with IMV/ECMO, five fewer SAEs, and 39 fewer deaths.

The probability of a more desirable outcome on remdesivir is 53.3% (95% CI: 50.8%, 55.9%; *P* = 0.01), indicating an overall remdesivir benefit. CI estimates for the cumulative DOOR probability are displayed in Fig. 1b. The probability of a more desirable outcome on remdesivir for the hospitalization with IMV/ECMO is 51.1% (95% CI: 49.4%, 52.9%), for SAEs is 50.2% (95% CI: 49.3%, 51.1%), and for death is 51.9% (95% CI: 49.9%, 53.9%) (Fig. 1a).

**Fig 2:**
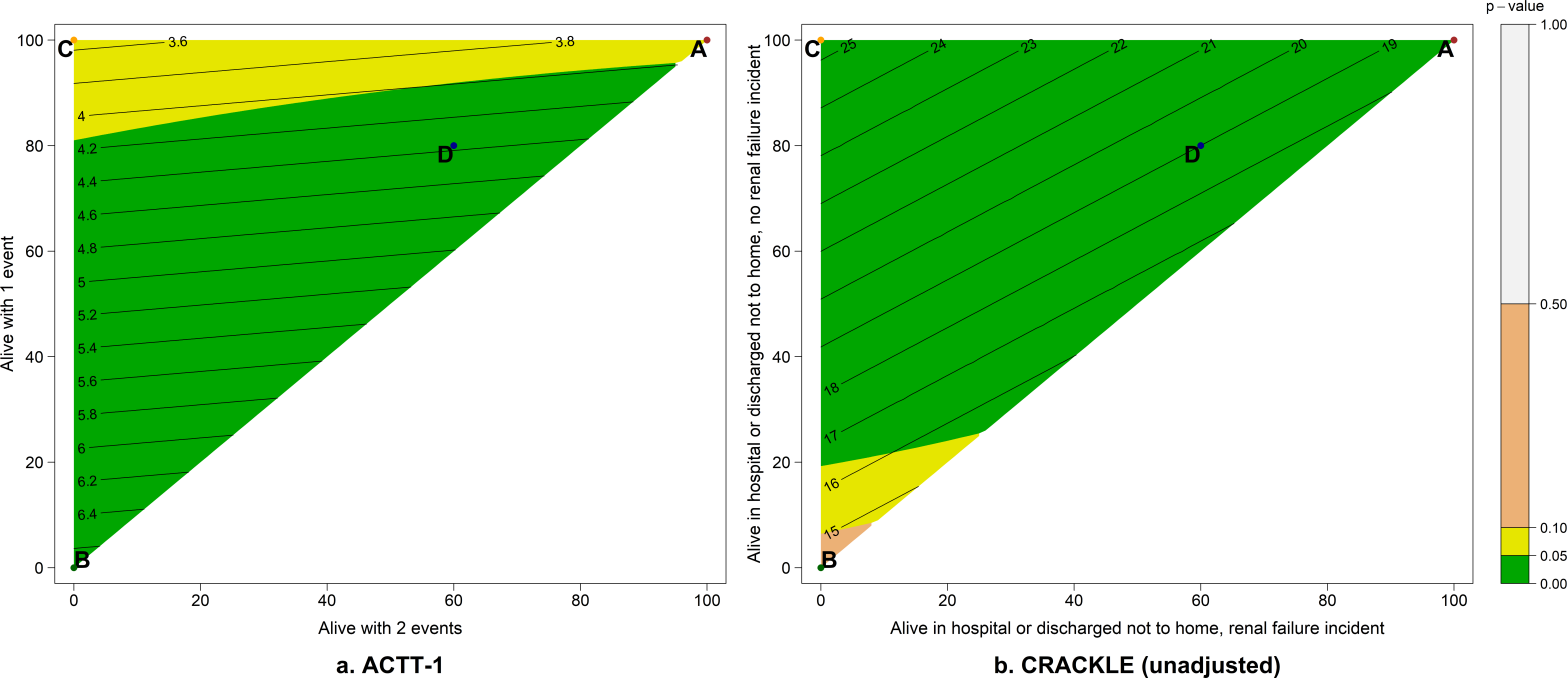
Contour plots of the between-group difference in means as the partial credit assigned to DOOR outcome level 2 (alive with one deleterious event) and level 3 (alive with both deleterious events) vary in (**a**) ACTT-1 and (**b**) CRACKLE (unadjusted).

Figure 2a displays a contour plot of the difference in means as the partial credit assigned to the DOOR outcome level 2 (alive with one deleterious event) and level 3 (alive with both deleterious events) vary. Green areas indicate grading key combinations that result in *P*-values less than 0.05.

### Consortium on resistance against carbapenems in *Klebsiella* and other Enterobacteriaceae (CRACKLE)

CRACKLE was a prospective, multicenter, observational study involving 18 hospitals from eight healthcare systems in the United States. One substudy compared ceftazidime-avibactam vs. colistin for the treatment of *Klebsiella pneumoniae* carbapenemase–producing CRE infections. A 4-level DOOR outcome was defined (from most to least desirable): (i) discharged home; (ii) alive in hospital or discharged not to home, no renal failure incident; (iii) alive in hospital or discharged not to home, renal failure incident; (iv) death (21). Inverse-probability of treatment weighting (IPTW) was utilized to account for potential confounding associated with the nonrandomized nature of the study.

Table 2 display the unadjusted and IPTW-adjusted (for baseline Pitt score and type of infection [21]) distribution of the DOOR outcome and respective components by treatment. The unadjusted probability of a more desirable outcome for ceftazidime-avibactam compared to colistin is 66.2% (95% CI: 54.4%, 76.3%; *P* = 0.01). The adjusted probability of a more desirable outcome for ceftazidime-avibactam compared to colistin is 64.3% (95% CI: 52.4%, 74.7%; *P* = 0.02), indicating an overall ceftazidime-avibactam benefit (Fig. 1c and d).

Figure 2b displays a contour plot of the difference in means as the partial credit assigned to DOOR outcome level 2 (alive in hospital or discharged not to home, no renal failure incident) and level 3 (alive in hospital or discharged not to home, renal failure incident) varies for the unadjusted distributions of the DOOR outcome.

## DISCUSSION

The provenance of the DOOR paradigm is to inform clinical decision-making by addressing a pragmatic question: how do patient experiences, when comprehensively considering benefits and harms, compare between therapeutic alternatives? For example, noninferiority (NI) trials typically aim to evaluate if an intervention with expected advantages in safety, quality of life (QoL), or convenience is not importantly less efficacious than control. However, a question more consistent with the pragmatic goal of informing clinical decision-making than “Is treatment A noninferior to B regarding the primary efficacy endpoint, if the purported advantage of A holds?” may be “Is treatment A superior to B by providing a favorable benefit:risk profile?” (22).

The recently published “Benefit:risk balance for medicinal products” from the Council for International Organizations of Medical Sciences (CIOMS) (23) includes two new points of emphasis: (i) transitioning benefit:risk evaluation from a *post hoc* exercise to a comprehensively integrated element of clinical trial design and conduct and (ii) a pragmatic patient-centric approach to benefit:risk assessment to ensure proper reflection and evaluation of the benefits and harms as experienced by patients. Specific recommendations include prespecifying benefit:risk DOOR endpoints representing a global patient outcome, in trial protocols in parallel with efficacy and safety endpoints, conducting DOOR analyses on these endpoints, and reporting analyses of these endpoints when publishing trial results in the medical literature and reporting results in trial registries.

The examples highlight important aspects of the DOOR paradigm: (i) it recognizes important tradeoffs of benefits and harms and (ii) significance on component outcomes does not necessarily imply significance on the DOOR outcome, and significance on the DOOR outcome does not necessarily imply significance on the component outcomes. In the DORI-05, acknowledging the *post hoc* nature of the analyses and the multiplicity issues associated with examination of multiple outcomes, doripenem displayed a more desirable result with respect to efficacy via avoidance of the absence of a clinical response (DOOR probability of 54.3% [95% CI: 51.1%, 57.4%]); however, a less desirable result with respect to safety via avoidance of infectious complications (DOOR probability of 47.6% [95% CI: 46.2%, 49.0%]) was observed. Although statistically significant efficacy advantages and safety disadvantages were observed, the DOOR outcome considers the benefit vs. harm tradeoff providing a compromise resulting a nonsignificant DOOR probability of 51.0% (95% CI: 47.6%, 54.3%) (Fig. 1a). In contrast, none of the component outcomes in the ACTT-1 trial are significant. However, the effects on all components favor remdesivir, and subsequent benefits of remdesivir amass, resulting in a significant overall DOOR probability of 53.3% (95% CI: 50.8%, 55.9%) (Fig. 1b). Similarly, none of the component outcomes in the CRACKLE are significant in the IPTW-adjusted analyses. Nonetheless, the point estimates of the effects on all components are in the same direction, and subsequent benefits of ceftazidime-avibactam amass, resulting in a significant overall DOOR probability of 64.3% (95% CI: 52.4%, 74.7%) (Fig. 1c and d).

The philosophical outline of the DOOR paradigm is as follows:

- Optimal pragmatism requires a patient-centric outcome, composing component outcomes to represent the patient experience
- Composites need not be binary; patient responses are more layered; components need not be treated as equivalent, e.g., death is more important than non-fatal events
- Composite endpoints require intricate analyses (all components should be evaluated individually to see if effects go in similar vs. opposing directions and to elucidate components driving the overall response; conduct cumulative evaluation of the outcome)
- The multiple outcome nature of the patient response necessitates an absolute risk scale
- Prioritize robustness, objectivity, error control, transparency, and avoidance of concessions of these principles during analyses

The guiding principles and SAP for DOOR are notable. Important points include that DOOR analyses do not replace traditional analyses of each efficacy and safety outcome. It builds upon it, composing the component outcomes toward patient-centric evaluation. Analyses of each component outcome are part of comprehensive DOOR analyses consistent with fundamental analyses of composite outcomes. It is important for analyses to occur on the absolute scale. Relative risk/ratio (RR) measures are challenging to interpret, convey no direct information about the individual risk of the occurrence of outcomes (22), and are contraindicated in multiple outcome settings. Suppose an intervention increases the risk of death from 1 in 10 to 2 in 10. This represents a RR = 2 and is very important. Suppose an intervention increases the risk of death from 1 in 10,000 to 2 in 10,000. This also represents a RR = 2 but is nearly irrelevant. How should an RR = 2 be interpreted? The challenge is compounded in multiple outcome settings. Suppose the efficacy rate is doubled (RR = 2) and that an equally important safety event rate is also doubled (RR = 2). This may lead to the belief that there is an equal tradeoff. But if the doubling of efficacy is 1 in 10 to 2 in 10 and the doubling of the safety event is 1 in 10,000 to 2 in 10,000, then the tradeoff is unequal. Interpreting relative risk/ratio measures from multiple outcomes is misleading due to different baseline risks. Absolute risk summaries are necessary when synthesizing the result of composite and multiple outcomes since component outcomes must be evaluated and interpreted simultaneously with other outcomes (24, 25).

The rank-based and partial credit DOOR analyses have two important advantages over the ordinal logistic regression model for the analyses of the DOOR outcome: (i) increased robustness and (ii) their absolute risk nature. The pinnacle of robust analyses is model- and assumption-free. The ordinal logistic model requires the assumption of proportional odds for validity (24). Rank-based analyses do not require distributional assumptions for validity. The validity of the partial credit analyses requires only sufficient sample size for the effects of the central limit theorem. Thus, the rank-based and partial credit analyses provide increased robustness over the ordinal logistic regression model. The ordinal logistic regression model typically produces estimates of odds ratios, relative measures that are contraindicated in benefit:risk evaluation (5, 18, 24, 25).

One of the DOOR analyses, the DOOR probability, has a relationship with the win ratio (26) or win odds (27). Both approaches compose multiple outcomes into unified, comprehensive analyses, DOOR through the ordinal DOOR outcome and the win ratio via ordered priorities (or hierarchical endpoint that can be considered a special case of a DOOR outcome with successive tiebreaks), though either statistic can be calculated using either composition strategy. Both statistics are calculated using pairwise comparisons of patient-centric responses. The approaches differ in other ways including the metric scale (relative vs. absolute risk), the handling of ties when comparing patient-centric responses, and resulting issues of statistical properties (e.g., whether there exists an unbiased estimator). The win ratio may be viewed as the relative risk version of the DOOR probability, an absolute risk measure. The generalized pairwise comparisons (GPC) (28) adapts ordered priorities, but uses the net treatment benefit (NTB) instead of the win ratio, where the NTB is calculated 2 × DOOR probability - 1, which makes it closely related—but not exactly identical—to the DOOR probability since the NTB is computed conditional on the previous ordered comparisons and combined accordingly. It is advisable to conduct benefit:risk and composite evaluation on an absolute scale as relative risk approaches are contraindicated in such settings. A fundamental tenet of composite endpoints is to analyze each component to understand treatment effect intricacies. Evaluating multiple outcomes using relative risk measures results in heterogeneous outcome scales since each outcome has its own baseline risk, complicating interpretation when evaluating multiple outcomes (see Section 1 in the Supplemental material). Absolute risk measures result in a common scale for all outcomes, allowing for simultaneous evaluation of multiple outcomes, critical for interpreting component evaluations (5, 18, 24, 28–30). The DOOR outcome allows for a tabular summary of the outcome by treatment and for grade-based analyses using partial credit. A comparison of DOOR to the win ratio with respect to statistical properties and qualifications for the guiding principles criteria (Table A9) and further details of the statistical relationships are provided in Section 6 of the Supplemental material.

A DOOR outcome may help with cases in which there are indeterminates arising when an evaluator is unable to classify a result as a clinical success or clinical failure because results lie within an intermediate “grey zone.” Here, indeterminates are distinct from completely missing information and should not arise due to challenges with evaluator adjudication of the reason for the patient status or experience as the outcome is an assessment of patient status or experience, and not an evaluation of its reason. With indeterminates recognized as an intermediate category, then DOOR analyses can be applied to a 3-level DOOR outcome: (i) clinical success; (ii) indeterminate, and (iii) clinical failure and may be more informative than other single imputation techniques.

DOOR can be tailored to specific diseases to inform clinical decision-making by focusing on patient-centric benefit:risk. The DOOR concept has been applied in intervention studies for several disease areas (7, 8, 10, 14, 21, 31–43). Researchers have proposed a DOOR that integrates patient preferences of outcome importance concluding that it can be used in pivotal or comparative effectiveness trials for a patient-centered evaluation of a therapeutic intervention (39). Industry sponsors are using DOOR, for example, in the development of interventions to treat cystic fibrosis pulmonary exacerbations (ClinicalTrials.gov ID: NCT05641298). The ALRG is using DOOR in the Bacteriophage Therapy in Cystic Fibrosis Subjects Colonized with *Pseudomonas aeruginosa* (PHAGE) (40) and Dalbavancin as an Option for Treatment of *S. aureus* bacteremia (DOTS) (41) clinical trials. DOOR has been recommended and applied to aid data and safety monitoring board (DSMB) evaluations (18, 44–46).

Ongoing work regarding DOOR includes the following: (i) subgroup analyses as subgroups should be identified and evaluated based on patient centric benefit:risk rather than limiting evaluation to a single variable; (ii) longitudinal evaluation of the DOOR outcome as a dynamic patient state (“sliding DOOR”) realizing the importance of not only knowing whether events occurred, but when, for how long, and whether they resolve or relapse, with the treatment effect characterized by the contrast in the time spent in the most and least desirable DOOR outcome categories (47, 48); (iii) group-sequential design and interim monitoring strategies (46); (iv) meta-analyses of multiple studies using DOOR to aid, e.g., in regulatory evaluation; (v) stratified and covariate-adjusted methods; (vi) cluster-randomization methods; (vii) multiarm clinical trials; (viii) refining of the online DOOR apps for qualification as regulatory science tools.

## AFTER EPUB

*[This article was published on 24 November 2025 with an error in the abstract. The abstract was corrected in the current version, posted on 2 December 2025.]*

## SUPPLEMENTAL MATERIAL

Supplemental material aac.01478-25-s0001.pdf Analysis of the DOOR outcomes.

ASM does not own the copyrights to Supplemental Material that may be linked to, or accessed through, an article. The authors have granted ASM a non-exclusive, world-wide license to publish the Supplemental Material files. Please contact the corresponding author directly for reuse.
