# Supplementary material for: A patient-centric paradigm and tool for clinical research: the DOOR is open
Source: Antimicrob Agents Chemother. 2025 Nov 24;70(1):e01478-25. doi: 10.1128/aac.01478-25 (PMC12777562; doi:10.1128/aac.01478-25)
Supplement: Supplemental material — Analysis of the DOOR outcomes. [file aac.01478-25-s0001.pdf]

Technical Report

The DOOR Methodology: Analysis of the DOOR Outcomes

Yijie He[1,2], Qihang Wu[1,2], Scott R. Evans, PhD[1,2], Toshimitsu Hamasaki, PhD[1,2,*]

November 19, 2025

[1] The Biostatistics Center, Milken Institute School of Public Health, George Washington University, Bethesda, Maryland, USA.

[2] Department of Biostatistics and Bioinformatics, Milken Institute School of Public Health, The George Washington University, Washington DC, USA

[*] Corresponding author: Toshimitsu Hamasaki, The Biostatistics Center, Milken Institute School of Public Health, The George Washington University, 6710A Rockledge Dr., Suite 250, Bethesda, Maryland 20817, USA, thamasaki@gwu.edu

Attached to

A patient-centric paradigm and tool for clinical research: the DOOR is open

Toshimitsu Hamasaki[1,2], Yijie He[1,2], Qihang Wu[1,2], Jessica-Howard Anderson[3], Helen Boucher[4], Sarah Doernberg[5], Thomas L Holland[6,7], John H Powers[8], Jing Wang[9], Guoqing Diao[1,2], David van Duin[10], Vance G Fowler[6,7], Henry F Chambers[5], Scott R. Evans [1,2]

[1] The Biostatistics Center, Milken Institute School of Public Health, George Washington University, Bethesda, Maryland, USA; [2] Department of Biostatistics and Bioinformatics, Milken Institute School of Public Health, George Washington University, Washington DC, USA; [3] Department of Medicine, Division of Infectious Diseases, Emory University School of Medicine, Atlanta, Georgia, USA; [4] Tufts University School of Medicine and Tufts Medicine, Boston, Massachusetts, USA; [5] Department of Medicine, Division of Infectious Diseases, University of California San Francisco, San Francisco, California, USA; [6] Department of Medicine, Duke University Medical Center, Durham, North Carolina, USA; [7] Duke Clinical Research Institute, Durham, North Carolina, USA, [8] Department of Medicine, George Washington University School of Medicine; [9] Biostatistics Research Branch, National Institute of Allergy and Infectious Diseases, Maryland, USA; [10] Division of Infectious Diseases, School of Medicine, University of North Carolina, Chapel Hill, North Carolina, USA

This work was supported by the National Institute of Allergy and Infectious Diseases of the NIH (award number UM1AI104681). The content is solely the responsibility of the authors and does not necessarily represent the official views of the National Institutes of Health (NIH).

TABLE OF CONTENTS

Table of Contents i

List of Abbreviations iii

1. Introduction 1

1.1 Issues to consider for improving benefit:risk analyses 1

Generalizability: To whom do analyses apply? 1

Challenge of interpreting individual outcomes: Competing risks 1

Absolute vs. relative risk 1

Cumulative and multidimensional effects on patients 2

The clinical trial arithmetic: Use patients to analyze outcomes or outcomes to analyze patients? 2

Subgroup evaluation and personalized medicine 4

1.2 Objectives 4

2. Rank-based Analysis: DOOR Probability-based Analysis 4

2.1 DOOR probability 5

2.2 Confidence intervals 6

Wald-type CIs 6

Halperin et al. (1989)'s CI 7

Transformation-based CIs 8

Pseudo-score CIs 9

Other CIs 11

Simulation study: Objectives and design 12

Simulation study: Results 13

2.3 Hypothesis tests 15

WMW test 15

Continuity correction and t-approximation 15

Transformation-based methods 16

Simulation study: Objectives and design 16

Simulation study: Results 17

2.4 Recommendations 18

3. Grade-based Analyses: Partial Credits 21

4. Guiding Principles, Recommended Statistical Analysis Plan, and Online DOOR Analysis Tools 22
5. Illustrations with Clinical Studies 24
5.1 DORI-05 24
5.2 ACTT-1 26
5.3 CRACKLE 27
6. Related Summary Measures, Paradigms, and Methods 29
6.1 Related summary measures 29
6.2 Related paradigms and methods 30
7. Summary and Further Developments 35
Acknowledgements 36
References 36
Appendix: Tables and Figures 41
Tables 41
Figures 52

LIST OF ABBREVIATIONS

Abbreviations	Full Description
APS	Anthology of Patient Stories
ARLG	The Antibacterial Resistance Leadership Group
AUC	Area Under the Curve
CI	Confidence Interval
DOOR	Desirability Of Outcome Ranking
ECMO	ExtraCorporeal Membrane Oxygenation
GPC	Generalized Pairwise Comparisons
HR	Hazard Ratio
IMV	Invasive Mechanical Ventilation
ITT	Intension-To-Treat
NTB	Net-Treatment-Benefit
ROC	Receiver Operating Characteristic
RR	Relative Risk (or Ratio)
SAE	Serious Adverse Event
SAP	Statistical Analysis Plan
WMW	Wilcoxon-Mann-Whitney

1. INTRODUCTION

1.1 Issues to consider for improving benefit:risk analyses

Randomized clinical trials are the gold standard for evaluating the benefits and harms of interventions, though often fail to provide the practical evidence to inform medical decision-making (DeMets and Califf, 2011). A primary reason is failure to recognize the most important questions for treating patients in clinical practice which are benefit:risk in nature, and using this as the motivation for the design, monitoring, analysis, and reporting of clinical trials and product development.

Standard approaches to benefit:risk evaluation synthesizing information obtained from separate marginal analysis of each outcome, do not address the most important questions for clinical practice as they are not patient-centric. They fail to incorporate associations between outcomes and recognize the cumulative nature of outcomes in individual patients, suffer from competing risk complexities during interpretation of component outcomes, and since efficacy and safety analyses are often conducted on different populations, generalizability to patient populations is unclear. Treatment effect heterogeneity is typically evaluated based on a single efficacy or safety endpoint, and rarely evaluated based on benefit:risk.

These challenges can be addressed by placing increased emphasis on patient-centric benefit:risk evaluation and questions of a pragmatic origin to match their clinical importance. The desirability of outcome ranking (DOOR) is a paradigm for the design, analysis, and interpretation of clinical trials and other research studies based on patient-centric benefit:risk evaluation (Evans et al., 2015; Evans and Follmann, 2016). The DOOR uses outcomes to analyze patients rather than patients to analyze outcomes by comparing the experiences of trial participants in different treatment arms by the desirability of the overall patient outcome.

Generalizability: To whom do analyses apply?

Analysis populations are carefully defined during the design and analysis of clinical trials. An intention-to-treat (ITT) population is typically used to analyze efficacy endpoints in late-phase trials. A distinct safety population is used for safety endpoints. Benefit:risk evaluation may combine these marginal analyses together. To whom does this benefit:risk analysis apply? The target population and estimand is not well-defined.

Challenge of interpreting individual outcomes: Competing risks

Suppose the duration of hospitalization is measured. Shorter duration is interpreted as better. However, the faster the patient dies (a competing risk), the shorter the duration. The interpretation of the duration of hospitalization needs the context of survival status. Summary statistics of duration of hospitalization are not interpretable unless survival status is known. However once survival status is established, then the duration of hospitalization has context for meaningful interpretation.

Absolute vs. relative risk

Suppose an intervention increases the risk of death from 1 in 10 to 2 in 10. This is a relative risk (RR) = 2 and very important. Now suppose and intervention increases the risk of death from 1 in

100,000 to 2 in 100,000. This is also a RR = 2 but nearly irrelevant. Is the RR the most informative measure when summarizing the impact of the intervention?

Consider the THALES clinical trial (Johnston et al., 2020), a randomized, double-blinded, placebo-controlled trial ($N = 11{,}016$) comparing “Ticagrelor and aspirin” (Ticagrelor) ($N = 5{,}523$) vs. aspirin and “Ticagrelor and Placebo” (Placebo) ($N = 5{,}493$) in acute ischemic stroke or TIA. The primary outcome was the time to stroke or death at 30 days resulting in a hazard ratio (HR) = 0.83 (95% Confidence Interval (CI): 0.71, 0.96, log-rank test: P = 0.015). The primary safety outcome was the time to severe bleeding by 30 days resulting in a HR = 3.99 (95% CI: 1.74, 9.14, log-rank test: P = 0.001). Is there too much bleeding relative to the benefits being observed?

Further examination of the primary outcome revealed that there were 303 events (5.5%) in the Ticagrelor arm and 362 events (6.6%) in the placebo arm. Ticagrelor saved 59 efficacy events in the trial. For the safety outcome, there were 28 events (0.5%) in the Ticagrelor arm and 7 events (0.1%) in the placebo arm. Ticagrelor cost 21 safety events. If the events are comparable, then there was a total savings from Ticagrelor of 38 events.

Suppose instead that for the primary safety outcome of severe bleeding, the results were 10 events for Ticagrelor and 1 event for placebo. This represents a HR = 10. This sounds worse than the earlier HR = 4. However, the cost is only 9 events resulting in a total savings of 50 events i.e., a better overall result for Ticagrelor. Comparing HRs from multiple outcomes can be misleading due to the different baseline risks. Absolute risks summaries are more appropriate when synthesizing the result of multiple endpoints.

Cumulative and multidimensional effects on patients

It is important to recognize that patients experience the cumulative and multidimensional effects of an intervention. The Antibacterial Resistance Leadership Group (ARLG) (https://arlg.org/) conducted a study in *Staphylococcus aureus* bacteremia (Doernberg et al. 2019). Twenty representative patient profiles summarizing the major events and outcomes (benefits, harms, and quality-of-life (QoL)), were constructed based on experiences observed in prior trials. The profiles were sent to 43 expert clinicians. The clinicians were asked to rank the patient profiles by the “desirability” of the overall patient experience. Factors driving clinician rankings were evaluated. Findings revealed that the cumulative nature of events was a major driver of clinician ranking e.g., patients that had clinical failure and non-fatal serious adverse events (SAEs) were ranked as having a worse experience than patients that had clinical failure without non-fatal SAE. This is intuitive though goes unrecognized when analyses consist of separate marginal analyses of each outcome.

The clinical trial arithmetic: Use patients to analyze outcomes or outcomes to analyze patients?

The culture in benefit:risk evaluation has been to use the patients to analyze the outcomes. Typically, in trials, the first endpoint is analyzed; results in Treatment A are aggregated, results in Treatment B are aggregated, and then treatments are compared. This process is repeated for all of the other endpoints. Benefit:risk analyses are often conducted by combining the separate marginal analyses together in some way. Unfortunately, this approach does not compose data in a manner consistent with the way the outcomes are experienced by patients.

Table 1. Evaluation of three treatments A, B, and C with treatment success and safety event

		Treatment success		
Treatment	Safety event	Yes	No	Total
A	Yes	15	15	30
	No	35	35	70
	Total	50	50	100
B	Yes	50	0	50
	No	0	50	50
	Total	50	50	100
C	Yes	0	50	50
	No	50	0	50
	Total	50	50	100

Suppose a person is diagnosed with a serious disease. Treatment is being selected among three treatment options, A, B, and C. A trial comparing these alternatives was conducted, shown in Table 1 (Evans, 2022). There are two major outcomes that are considered equally important: (i) a binary treatment success (Yes/No), and (ii) a binary safety event (Yes/No). There were 100 patients in each treatment arm. The proportion of patients with treatment success (Yes) was 50% (50/100) in Treatment A, 50% (50/100) in Treatment B and 50% (50/100) in Treatment C. The proportion of patients with safety event (Yes) was 30% (30/100) in Treatment A, 50% (50/100) in Treatment B and 50% (50/100) in Treatment C. Which treatment do you choose? They all have the same success proportion, and A has the lowest safety event proportion. Treatments B and C are indistinguishable. Clearly Treatment A should be chosen.

These analyses are the typical approach to benefit:risk analyses. Patients are randomized, followed over time, and used to analyze the outcomes. However, this strategy is inverted, instead "using the outcomes to analyze the patients rather than patients to analyze the outcomes".

There are four possible "patient outcomes". A patient may experience treatment success with or without the safety event, or they may not experience treatment success with or without the safety event. Treatment success and safety outcomes can be cross-classified to examine the distribution of the patient outcomes by treatment arm. In Treatment A, there was no correlation between the success and the safety event, resulting in 35 patients that experienced the treatment success and avoided the safety problem. In Treatment B, the outcomes were positively correlated resulting in zero patients with success without the safety event. In Treatment C, the outcomes were negatively correlated resulting in 50 patients that experienced success and avoided the safety event. This is striking since the typical analyses was unable to distinguish between Treatments B and C though they are importantly different. Since treatment success and the safety event have similar importance, nobody assigned to Treatment B had a net benefit. In contrast, Treatment C may be a good treatment if the right subgroup of patients for its application can be identified. Typical analyses combining marginal effects are blind to this difference. Critical thought is needed regarding how to aggregate data to describe treatment effects on patients and better inform medical decision-making. The purpose of measuring the outcomes in the trial is to inform patient status particularly in late-phase trials where there is a focus on describing and making inferences regarding the disease burden and impact on patients.

A global outcome for the patient is needed. Component outcomes may be used to holistically evaluate the patient's status and experience. Aggregations over treatments A and B can then be made, and the treatments can be compared. This evaluation reflects how treatments compare with respect to their effect on patients (Evans and Follmann, 2016).

Subgroup evaluation and personalized medicine

How do we identify the subgroup of patients we want to treat with a particular intervention? Recall Table 1 summarizing the results of the trial comparing A, B, and C. Predictive markers for efficacy may identify the 50 patients in the treatment success column for each treatment. However, this includes patients with safety concerns, e.g., 15 in Treatment A and all 50 in Treatment B. Predictive markers for safety may identify the 70 patients in Treatment A and 50 patients in Treatment B, in the bottom row for each treatment. However, this includes patients that do not experience efficacy, e.g., 35 in Treatment A and all 50 in Treatment B. Subgroup identification based only on efficacy or safety does not identify the patients that should be treated. Treatment effect heterogeneity, subgroup identification, and effect estimates should be based upon patient-centric benefit:risk analysis to more optimally inform therapeutic decision-making.

1.2 Objectives

In this technical report, we describe statistical issues in the implementation of the DOOR methodology, in particular two statistical methods for the analysis of clinical trials and other clinical research using the DOOR methodology, the rank-based approach and the grade-based analysis. We provide the recommended statistical analysis plan for the DOOR methodology and illustrate the statistical analyses for DOOR outcomes with three clinical studies.

2. RANK-BASED ANALYSIS: DOOR PROBABILITY-BASED ANALYSIS

The DOOR adopts an outcome with gradations of patient response to allow recognition of important differences in ultimate responses resulting from a therapeutic intervention, representing a global patient response constructed on the basis of important clinical benefits, harms, and possibly quality of life. The ordinal "DOOR outcome" reflects the trade-offs among component outcomes and the cumulative nature of benefits and harms to patients. The definition of the DOOR outcome is tailored to the disease of interest, and the clinical research question. In many disease areas, researchers have proposed a DOOR outcome that integrates patient preferences of outcome importance and concluded that it can be used in pivotal trials or comparative effectiveness trials for a patient-centered evaluation of a therapeutic intervention (e.g., see Chamberlain et al. (2023), Gewandter et al. (2018), Evans et al. (2020), Kinamon et al. (2023), Kleykamp et al. (2022), Lu et al. (2022), Lodise et al. (2025), Sandoval et al. (2023), and so on).

One analysis in the DOOR paradigm compares the outcome between interventions using rank-based methods and estimating the DOOR probability, i.e., the probability that a participant assigned to one intervention has a more desirable outcome than a participant assigned to the other intervention. In this section, we review statistical methods for constructing a CI and testing a hypothesis for the DOOR probability and discuss improved methods. We evaluate the performance of the methods by a simulation. We provide practical guidance on the use of confidence intervals and hypothesis tests associated with the DOOR probability.

Table 2. A summary table of a DOOR outcome with K-ranked categories by two intervention groups

	Experimental (E)		Control (C)	
DOOR outcome category	Proportion	Frequency	Proportion	Frequency
1: most desirable	P_1^{E}	n_1^{E}	P_1^{C}	n_1^{C}
⋮	⋮	⋮	⋮	⋮
k	P_k^{E}	n_k^{E}	P_k^{C}	n_k^{C}
⋮	⋮	⋮	⋮	⋮
K: least desirable	P_K^{E}	n_K^{E}	P_K^{C}	n_K^{C}
Total	$\Sigma_{k=1}^{K} P_k^{\mathrm{E}} = 1$	$n^{\mathrm{E}} = \Sigma_{k=1}^{K} n_k^{\mathrm{E}}$	$\Sigma_{k=1}^{K} P_k^{\mathrm{C}} = 1$	$n^{\mathrm{C}} = \Sigma_{k=1}^{K} n_k^{\mathrm{C}}$

2.1 DOOR probability

Consider a comparison of experimental (E) vs. control (C), based on a DOOR outcome with K-ranked categories. The distribution of DOOR outcome is summarized by intervention in Table 2, having K-ranked categories with corresponding proportion distributions $\boldsymbol{P}^g = (P_1^g, \ldots, P_K^g)$, where $\Sigma_{k=1}^{K} P_k^g = 1 (g = \mathrm{E}, \mathrm{C})$. Let Y_i^{E} and Y_j^{C} denote independent random variables having the same ordinal scale ($i = 1, \ldots, n^{\mathrm{E}}; j = 1, \ldots, n^{\mathrm{C}}$), where $Y_1^{\mathrm{E}}, \ldots, Y_{n^{\mathrm{E}}}^{\mathrm{E}}$ are from $\boldsymbol{P}^{\mathrm{E}} = (P_1^{\mathrm{E}}, \ldots, P_K^{\mathrm{E}})$ and $Y_1^{\mathrm{C}}, \ldots, Y_{n^{\mathrm{C}}}^{\mathrm{C}}$ are from $\boldsymbol{P}^{\mathrm{C}} = (P_1^{\mathrm{C}}, \ldots, P_K^{\mathrm{C}})$, where the (g, k) category count n_k^g corresponds to the number of Y_i^g's that equal Rank k, and the K-ranked categories are labeled as 1, 2, …, K, from most to least desirable. Each group simple size is $n^g = \Sigma_{k=1}^{K} n_k^g$ and the sample size allocation is $a^g = n^g / n$, where $n = n^{\mathrm{E}} + n^{\mathrm{C}}$ is the total sample size.

The distribution of the DOOR outcomes is compared between two groups, using a rank-based approach, with the probability of a participant assigned to one group having a more desirable outcome than a participant assigned to the other group, i.e., the "DOOR probability" as the primary summary measure. The probability is defined by

$$\pi_{\mathrm{E} \geq \mathrm{C}} = \mathbb{P}[Y^{\mathrm{E}} > Y^{\mathrm{C}}] + 1/2 \mathbb{P}[Y^{\mathrm{E}} = Y^{\mathrm{C}}].$$

This measure has been studied by many researchers under different names, such as pivotal quantity, Wilcoxon-Mann-Whitney (WMW) parameter, ordinal effect size measure, and other (e.g., see Halperin, Hamdy and Thall (1989), Ryu and Agresti (2008)). In this report, we refer to it as the "DOOR probability". The DOOR probability is $\pi_{\mathrm{E} \geq \mathrm{C}} = 0.5$ if Y^{E} and Y^{C} are identically distributed. If categories are reversed in order or if Y^{E} and Y^{C} are interchanged, then

$$\pi_{\mathrm{E} \leq \mathrm{C}} = \mathbb{P}[Y^{\mathrm{E}} < Y^{\mathrm{C}}] + 1/2 \mathbb{P}[Y^{\mathrm{E}} = Y^{\mathrm{C}}] = 1 - \pi_{\mathrm{E} \geq \mathrm{C}}.$$

The DOOR probability will probably be simplified to $\pi_{\mathrm{E} \geq \mathrm{C}} = \mathbb{P}[Y^{\mathrm{E}} > Y^{\mathrm{C}}]$ if the variables are continuous. The DOOR probability incorporates differences in both location and dispersion of the two ranks (Simonoff, Hochberg and Reiser, 1986).

The probability $\pi_{\mathrm{E} \geq \mathrm{C}}$ can be estimated by Wilcoxon-Mann-Whitney (WMW) statistic corrected for ties, divided by the product of the numbers of participants $n^{\mathrm{E}} n^{\mathrm{C}}$ (Wilcoxon 1945; Mann and Whitney 1947). It is given by

$$\hat{\pi}_{\mathrm{E} \geq \mathrm{C}} = \frac{1}{n^{\mathrm{E}} n^{\mathrm{C}}} \Sigma_{i=1}^{n^{\mathrm{E}}} \Sigma_{j=1}^{n^{\mathrm{C}}} \phi(Y_i^{\mathrm{E}}, Y_j^{\mathrm{C}}),$$

where

$$\phi\left(Y_i^{\mathrm{E}}, Y_j^{\mathrm{C}}\right) = \begin{cases} 1, & \text{if } Y_i^{\mathrm{E}} > Y_j^{\mathrm{C}}, \\ 1/2, & \text{if } Y_i^{\mathrm{E}} = Y_j^{\mathrm{C}}, \\ 0, & \text{if } Y_i^{\mathrm{E}} < Y_j^{\mathrm{C}}. \end{cases}$$

For the DOOR outcomes with K-ranked categories, with the estimates of the proportions for each category p_k^g, assuming independent multinomial sampling, the unrestricted maximum likelihood estimate $p_k^g = n_k^g/n^g$, an unbiased estimate of the DOOR probability $\hat{\pi}_{\mathrm{E}\geq\mathrm{C}}$ is calculated given the estimates of the proportions for each category p_k^g, which is given by

$$\hat{\pi}_{\mathrm{E}\geq\mathrm{C}} = \Sigma_{k=1}^{K-1} p_k^{\mathrm{E}} \Sigma_{k'=k+1}^{K} p_{k'}^{\mathrm{C}} + \frac{1}{2}\Sigma_{k=1}^{K} p_k^{\mathrm{E}} p_k^{\mathrm{C}}$$

(e.g., see Halperin et al. (1989)). The first term is a sum of products of the category proportions in a rank for one intervention and the sum of the category proportions in worse ranks for the other intervention. The second is a sum of the products of the category proportions in the same rank for the test and control interventions, divided by two. As an alternative method, one may be interested in using regression models for ordinal responses such as cumulative logit regression (or proportional odds regression) to estimate the proportions for each category. The regression models require additional assumptions (e.g., proportional odds) for validity. If the model assumption is violated, the DOOR probability estimate will be biased and the statistical errors (e.g., the Type I error) will not be well controlled. We prefer using simpler, more robust approaches than complex modeling approaches.

2.2 Confidence intervals

Existing CI estimation methods for the DOOR probability are reviewed and their improved methods are discussed.

Wald-type CIs

As the DOOR probability is estimated using the WMW statistic, it is natural to construct the CI for the DOOR probability based on the WMW statistic-based quantity

$$z_{\mathrm{DOOR}} = \frac{\hat{\pi}_{\mathrm{E}\geq\mathrm{C}} - \pi_{\mathrm{E}\geq\mathrm{C}}}{\sqrt{\mathbb{V}\mathrm{ar}[\hat{\pi}_{\mathrm{E}\geq\mathrm{C}}]}}.$$

Wald-type CIs for the DOOR probability $\pi_{\mathrm{E}\geq\mathrm{C}}$ are constructed based on the standard error of the DOOR probability (Ryu and Agresti, 2008). Using the delta method, the first-order approximation to $\mathbb{V}\mathrm{ar}[\hat{\pi}_{\mathrm{E}\geq\mathrm{C}}]$ is

$$V \doteqdot \frac{1}{n^{\mathrm{E}}}[A - \pi_{\mathrm{E}\geq\mathrm{C}}^2] + \frac{1}{n^{\mathrm{C}}}[B - \pi_{\mathrm{E}\geq\mathrm{C}}^2],$$

where

$$A = \Sigma_{k=1}^{K-1} P_k^{\mathrm{E}} \left(\Sigma_{k'=k+1}^{K} P_{k'}^{\mathrm{C}} + \frac{P_k^{\mathrm{C}}}{2}\right)^2 + \frac{P_K^{\mathrm{E}}\left(P_K^{\mathrm{C}}\right)^2}{4} \text{ and}$$

$$B = \Sigma_{k'=2}^{K} P_{k'}^{\mathrm{C}} \left(\Sigma_{k=1}^{k'-1} P_k^{\mathrm{E}} + \frac{P_{k'}^{\mathrm{E}}}{2}\right)^2 + \frac{(P_1^{\mathrm{E}})^2 P_1^{\mathrm{C}}}{4}.$$

The exact variance of $\hat{\pi}_{\mathrm{E}\geq\mathrm{C}}$ has been discussed (Halperin et al., 1989; Ryu and Agresti, 2008; Tang, 2011). Using the iterated variance formula, it is given by

$$V_e = \frac{1}{n^{\mathrm{E}} n^{\mathrm{C}}}\left[\pi_{\mathrm{E}\geq\mathrm{C}} - (n-1)\pi_{\mathrm{E}\geq\mathrm{C}}^2 + (n^{\mathrm{C}}-1)A + (n^{\mathrm{E}}-1)B - \frac{1}{4}\Sigma_{k=1}^{K} P_k^{\mathrm{E}} P_k^{\mathrm{C}}\right].$$

$(\hat{\pi}_{\mathrm{E}\geq\mathrm{C}} - \pi_{\mathrm{E}\geq\mathrm{C}})/V^{1/2}$ is asymptotically normally distributed as $\mathrm{N}(0,1)$ for large samples (e.g., see Mann and Whitney (1947)). Let $\hat{V}$ and $\hat{V}_e$ be the estimates of V and V_e, with $\hat{\pi}_{\mathrm{E}\geq\mathrm{C}}$, p_k^{E}, and p_k^{C} plugged into V and V_e. With $\hat{V}$ and $\hat{V}_e$, the $100(1-\alpha)\%$ two-sided Wald-type confidence limits for $\pi_{\mathrm{E}\geq\mathrm{C}}$ are

$$\left(\hat{\pi}_{\mathrm{E}\geq\mathrm{C}} - z_{\alpha/2}\hat{V}^{1/2}, \hat{\pi}_{\mathrm{E}\geq\mathrm{C}} + z_{\alpha/2}\hat{V}^{1/2}\right) \text{ and } \left(\hat{\pi}_{\mathrm{E}\geq\mathrm{C}} - z_{\alpha/2}\hat{V}_e^{1/2}, \hat{\pi}_{\mathrm{E}\geq\mathrm{C}} + z_{\alpha/2}\hat{V}_e^{1/2}\right),$$

where $z_{\alpha/2}$ is the $(1-\alpha/2)$ quantile of the standard normal distribution. In extreme situations where the DOOR probability is close to zero or 100%, especially with small sample sizes, the lower or upper bounds of the Wald-type CIs are estimated to be below zero or above 100%. In addition, if there is no overlap in the DOOR outcome distribution between groups, which results in only two ordered categories, or if all patients in both groups fall into only one DOOR category, then the variance is estimated to be zero and thus the Wald-type CIs cannot be obtained.

Halperin et al. (1989)'s CI

Halperin et al. (1989) derived the modified version of $\hat{V}_e$, ignoring asymptotically negligible terms, which is given by

$$\hat{V}_{\mathrm{HHT}} = \frac{1}{n^{\mathrm{E}} n^{\mathrm{C}}}\left[(n-1) - (n-2)\hat{\theta}\right]\widehat{\pi_{\mathrm{E}\geq\mathrm{C}}(1-\pi_{\mathrm{E}\geq\mathrm{C}})},$$

where

$$\hat{\theta} = \frac{(n-2)\hat{\pi}_{\mathrm{E}\geq\mathrm{C}} - (n^{\mathrm{C}}-1)\hat{\hat{A}} - (n^{\mathrm{E}}-1)\hat{\hat{B}}}{(n-2)\widehat{\pi_{\mathrm{E}\geq\mathrm{C}}(1-\pi_{\mathrm{E}\geq\mathrm{C}})}}$$

$$\hat{\hat{A}} = \hat{A} - \frac{1}{n^{\mathrm{C}}-1}\Sigma_{k=1}^{K-1} p_k^{\mathrm{E}}\left[(1-p_k^{\mathrm{C}})\Sigma_{k'=k+1}^{K} p_{k'}^{\mathrm{C}} - \left(\Sigma_{k'=k+1}^{K} p_{k'}^{\mathrm{C}}\right)^2\right] - \frac{1}{4(n^{\mathrm{C}}-1)}\Sigma_{k=1}^{K} p_k^{\mathrm{E}} p_k^{\mathrm{C}}(1-p_k^{\mathrm{C}})$$

$$\hat{\hat{B}} = \hat{B} - \frac{1}{n^{\mathrm{E}}-1}\Sigma_{k'=2}^{K} p_{k'}^{\mathrm{C}}\left[(1-p_{k'}^{\mathrm{E}})\Sigma_{k=1}^{k'-1} p_k^{\mathrm{E}} - \left(\Sigma_{k=1}^{k'-1} p_{k'}^{\mathrm{E}}\right)^2\right] - \frac{1}{4(n^{\mathrm{E}}-1)}\Sigma_{k'=1}^{K} p_{k'}^{\mathrm{C}} p_{k'}^{\mathrm{E}}(1-p_{k'}^{\mathrm{E}}), \text{ and}$$

$$\widehat{\pi_{\mathrm{E}\geq\mathrm{C}}(1-\pi_{\mathrm{E}\geq\mathrm{C}})} = \frac{(n^{\mathrm{E}}n^{\mathrm{C}} - n + 2)\hat{\pi}_{\mathrm{E}\geq\mathrm{C}} - n^{\mathrm{E}}n^{\mathrm{C}}\hat{\pi}_{\mathrm{E}\geq\mathrm{C}}^2}{(n^{\mathrm{E}}-1)(n^{\mathrm{C}}-1)} + \frac{\hat{\hat{A}}}{n^{\mathrm{E}}-1} + \frac{\hat{\hat{B}}}{n^{\mathrm{C}}-1}.$$

Substituting $\hat{V}_{\mathrm{HHT}}$ into $z_{DOOR}^2 = (\hat{\pi}_{\mathrm{E}\geq\mathrm{C}} - \pi_{\mathrm{E}\geq\mathrm{C}})^2/\hat{V}_{\mathrm{HHT}}$ yields the quadratic inequity

$$n^{\mathrm{E}}n^{\mathrm{C}}\frac{(\hat{\pi}_{\mathrm{E}\geq\mathrm{C}} - \pi_{\mathrm{E}\geq\mathrm{C}})^2}{\gamma\pi_{\mathrm{E}\geq\mathrm{C}}(1-\pi_{\mathrm{E}\geq\mathrm{C}})} \leq \chi_{1-\alpha}^2(1)$$

By solving the quadratic inequity, the Halperin et al. (1989)'s $100(1-\alpha)\%$ two-sided confidence limits for $\pi_{\mathrm{E}\geq\mathrm{C}}$ are given by

$$\left(\frac{v+2\hat{\pi}_{\mathrm{E}\geq\mathrm{C}}-\sqrt{v^2+4v\hat{\pi}_{\mathrm{E}\geq\mathrm{C}}(1-\hat{\pi}_{\mathrm{E}\geq\mathrm{C}})}}{2(v+1)},\frac{v+2\hat{\pi}_{\mathrm{E}\geq\mathrm{C}}+\sqrt{v^2+4v\hat{\pi}_{\mathrm{E}\geq\mathrm{C}}(1-\hat{\pi}_{\mathrm{E}\geq\mathrm{C}})}}{2(v+1)}\right),$$

where $\gamma=(n-1)-(n-2)\hat{\theta}, v=\gamma\chi^2_{1-\alpha}(1)/(n^{\mathrm{E}}n^{\mathrm{C}})$, and $\chi^2_{1-\alpha}(1)$ denotes the $(1-\alpha)$ quantile of the chi-square distribution with one degree of freedom. If there is no overlap in the DOOR outcome distribution between groups, then $\widehat{\hat{A}}=\hat{\hat{B}}=0$. Halperin et al. (1989) suggested $\hat{\theta}$ should be set to zero be set to zero, even though this leads to a "conservative" CI estimate, meaning that the actual coverage probability exceeds the nominal level.

Transformation-based CIs

Simonoff, Hochberg and Reiser (1988) suggested the use of inverse hyperbolic tangent ($\tanh^{-1}$) transformation for Somers' d (Somers, 1962), to construct the CI, which is $2\tanh^{-1}(d)=\log[(1+d)/(1-d)]$ (Edwardes, 1995). When $2\times K$ table, as Somers' d can be represented by using the DOOR probability, i.e., $d=2\pi_{\mathrm{E}\geq\mathrm{C}}-1$, this transformation is simply implemented to $\hat{\pi}_{\mathrm{E}\geq\mathrm{C}}$ for $2\tanh^{-1}(\hat{\pi}_{\mathrm{E}\geq\mathrm{C}})=\log[\hat{\pi}_{\mathrm{E}\geq\mathrm{C}}/(1-\hat{\pi}_{\mathrm{E}\geq\mathrm{C}})]$. Using the delta method, the first-order approximation to $\mathbb{V}\mathrm{ar}[\log\hat{\varphi}_{\mathrm{E}\geq\mathrm{C}}]$ is given by

$$\mathbb{V}\mathrm{ar}[\log\hat{\varphi}_{\mathrm{E}\geq\mathrm{C}}]\doteqdot\frac{\mathbb{V}\mathrm{ar}[\hat{\pi}_{\mathrm{E}\geq\mathrm{C}}]}{[\pi_{\mathrm{E}\geq\mathrm{C}}(1-\pi_{\mathrm{E}\geq\mathrm{C}})]^2},$$

where $\hat{\varphi}_{\mathrm{E}\geq\mathrm{C}}=\hat{\pi}_{\mathrm{E}\geq\mathrm{C}}/(1-\hat{\pi}_{\mathrm{E}\geq\mathrm{C}})$. If the estimate of $\mathbb{V}\mathrm{ar}[\log\hat{\varphi}_{\mathrm{E}\geq\mathrm{C}}]$ is calculated by $\hat{V}/[(\hat{\pi}_{\mathrm{E}\geq\mathrm{C}}(1-\hat{\pi}_{\mathrm{E}\geq\mathrm{C}})]^2$, then the $100(1-\alpha)\%$ two-sided Wald-type confidence limits for $\log\varphi_{\mathrm{E}\geq\mathrm{C}}$ are

$$\left(\log\hat{\varphi}_{\mathrm{E}\geq\mathrm{C}}-z_{\alpha/2}\frac{\sqrt{\hat{V}}}{\hat{\pi}_{\mathrm{E}\geq\mathrm{C}}(1-\hat{\pi}_{\mathrm{E}\geq\mathrm{C}})},\log\hat{\varphi}_{\mathrm{E}\geq\mathrm{C}}+z_{\alpha/2}\frac{\sqrt{\hat{V}}}{\hat{\pi}_{\mathrm{E}\geq\mathrm{C}}(1-\hat{\pi}_{\mathrm{E}\geq\mathrm{C}})}\right).$$

Therefore, by back-transforming the above limits, the $100(1-\alpha)\%$ two-sided $\tanh^{-1}$-based confidence limits for $\pi_{\mathrm{E}\geq\mathrm{C}}$ are

$$\left(\frac{\exp(LL_{\tanh})}{1+\exp(LL_{\tanh})},\frac{\exp(UL_{\tanh})}{1+\exp(UL_{\tanh})}\right),$$

where $LL_{\tanh}=\log\hat{\varphi}_{\mathrm{E}\geq\mathrm{C}}-z_{\alpha/2}\sqrt{\hat{V}}/[\hat{\pi}_{\mathrm{E}\geq\mathrm{C}}(1-\hat{\pi}_{\mathrm{E}\geq\mathrm{C}})]$ and $UL_{\tanh}=\log\hat{\varphi}_{\mathrm{E}\geq\mathrm{C}}+z_{\alpha/2}\sqrt{\hat{V}}/[\hat{\pi}_{\mathrm{E}\geq\mathrm{C}}(1-\hat{\pi}_{\mathrm{E}\geq\mathrm{C}})]$. This CI was discussed as the Wald-type CI for the logit of the DOOR probability in Ryu and Agresti (2008).

The arcsine square root transformation is often used on binomial proportions to stabilize the variance of the proportions (Bartlett, 1948), and it is known that variance stabilizing transformations can improve the normality of the statistical quantities. Chao and Cheng (1985) discussed the arcsine square root transformation to the DOOR probability when an outcome is continuous. Using the delta method, the first-order approximation to $\mathbb{V}\mathrm{ar}\left[\arcsin\sqrt{\hat{\pi}_{\mathrm{E}\geq\mathrm{C}}}\right]$

$$\mathbb{V}\mathrm{ar}\left[\arcsin\sqrt{\hat{\pi}_{\mathrm{E}\geq\mathrm{C}}}\right]\doteqdot\frac{\mathbb{V}\mathrm{ar}[\hat{\pi}_{\mathrm{E}\geq\mathrm{C}}]}{4\pi_{\mathrm{E}\geq\mathrm{C}}(1-\pi_{\mathrm{E}\geq\mathrm{C}})}.$$

If $\mathbb{V}\text{ar}\left[\arcsin\sqrt{\hat{\pi}_{\text{E}\geq\text{C}}}\right]$ is estimated by $\hat{V}/[4\hat{\pi}_{\text{E}\geq\text{C}}(1-\hat{\pi}_{\text{E}\geq\text{C}})]$, then the$100(1-\alpha)\%$ two-sided Wald-type confidence limits for $\arcsin\sqrt{\pi_{\text{E}\geq\text{C}}}$ are

$$\left(\arcsin\sqrt{\hat{\pi}_{\text{E}\geq\text{C}}} - z_{\alpha/2}\frac{\sqrt{\hat{V}}}{2\sqrt{\hat{\pi}_{\text{E}\geq\text{C}}(1-\hat{\pi}_{\text{E}\geq\text{C}})}}, \arcsin\sqrt{\hat{\pi}_{\text{E}\geq\text{C}}} + z_{\alpha/2}\frac{\sqrt{\hat{V}}}{2\sqrt{\hat{\pi}_{\text{E}\geq\text{C}}(1-\hat{\pi}_{\text{E}\geq\text{C}})}}\right).$$

Therefore, by back-transforming the above limits, the $100(1-\alpha)\%$ two-sided arcsine square root transformation-based confidence limits for $\pi_{\text{E}\geq\text{C}}$ are

$$((\sin LL_{\text{arcsin}})^2, (\sin UL_{\text{arcsin}})^2),$$

where $LL_{\text{arcsin}} = \arcsin\sqrt{\hat{\pi}_{\text{E}\geq\text{C}}} - z_{\alpha/2}\sqrt{\hat{V}}/[2\sqrt{\hat{\pi}_{\text{E}\geq\text{C}}(1-\hat{\pi}_{\text{E}\geq\text{C}})}]$ and $UL_{\text{arcsin}} = \arcsin\sqrt{\hat{\pi}_{\text{E}\geq\text{C}}} + z_{\alpha/2}\sqrt{\hat{V}}/[2\sqrt{\hat{\pi}_{\text{E}\geq\text{C}}(1-\hat{\pi}_{\text{E}\geq\text{C}})}]$. Similar to the Wald-type CIs, if there is no overlap in the DOOR outcome distribution between groups, or if all patients in both groups fall into only one DOOR category, the variance is estimated to be zero and thus the transformation-based CIs cannot be obtained.

Pseudo-score CIs

Our initial investigation suggests that the Wald-type CIs are always "liberal" i.e., the coverage probability is below the nominal level, and the Halperin et al. (1989)'s and $\tanh^{-1}$ transformation-based CIs generally work well even with small sample sizes, controlling for the coverage probability at the nominal level, although they tend to be liberal when the total sample size is less than 200 to 300 and the sample size per group is extremely imbalanced (e.g., 4:1 or greater sample size allocation ratio). As discussed in Ryu and Agresti (2008), using a pseudo-score-approach may be one way to improve the liberalness. The pseudo-score CIs require the variance of $\hat{\pi}_{\text{E}\geq\text{C}}$ under the null hypothesis $\text{H}_0: \pi_{\text{E}\geq\text{C}} = \pi^0_{\text{E}\geq\text{C}}$.

Ryu and Agresti (2008) discussed pseudo-score Wald-type CI, and the $100(1-\alpha)\%$ two-sided CI for $\pi_{\text{E}\geq\text{C}}$ is the set of $\pi^0_{\text{E}\geq\text{C}}$ that satisfies

$$\frac{(\hat{\pi}_{\text{E}\geq\text{C}} - \pi^0_{\text{E}\geq\text{C}})^2}{\hat{V}(\pi^0_{\text{E}\geq\text{C}})} < \chi^2_{1-\alpha}(1),$$

where $\hat{V}(\pi^0_{\text{E}\geq\text{C}})$ is the variance estimate of V given $\pi^0_{\text{E}\geq\text{C}}$. An extension of the pseudo-score CI to the Halperin et al. (1989)'s and the tahn^{-1} transformation-based CIs is straightforward. For the Halperin et al. (1989)'s CI, the$100(1-\alpha)\%$ two-sided CI for $\pi_{\text{E}\geq\text{C}}$ is the set of $\pi^0_{\text{E}\geq\text{C}}$ that satisfies

$$\frac{(\hat{\pi}_{\text{E}\geq\text{C}} - \pi^0_{\text{E}\geq\text{C}})^2}{\hat{V}_{\text{HHT}}(\pi^0_{\text{E}\geq\text{C}})} < \chi^2_{1-\alpha}(1)$$

and for the tahn^{-1} transformation-based CI, the $100(1-\alpha)\%$ two-sided CI for $\log\varphi_{\text{E}\geq\text{C}}$ is the set of $\pi^0_{\text{E}\geq\text{C}}$ that satisfies

$$\frac{(\log\hat{\varphi}_{\text{E}\geq\text{C}} - \log\varphi^0_{\text{E}\geq\text{C}}(\pi^0_{\text{E}\geq\text{C}}))^2}{\hat{V}_{\log\varphi^0_{\text{E}\geq\text{C}}}(\pi^0_{\text{E}\geq\text{C}})} < \chi^2_{1-\alpha}(1),$$

where

Table 3. An algorithm to find the upper and lower limits of the pseudo-score type CIs

Upper limit

Step 1: Select the two initial values $\pi_{\mathrm{E}\geq\mathrm{C}}^{UL(0)}$ and $\pi_{\mathrm{E}\geq\mathrm{C}}^{UL(1)}$ for the upper limit (UL), and then calculate the pseudo score

$$PS^{UL(0)} = \frac{\left(\hat{\pi}_{\mathrm{E}\geq\mathrm{C}} - \pi_{\mathrm{E}\geq\mathrm{C}}^{UL(0)}\right)^2}{\hat{V}_*(\pi_{\mathrm{E}\geq\mathrm{C}}^{UL(0)})} \text{ and } PS^{UL(1)} = \frac{\left(\hat{\pi}_{\mathrm{E}\geq\mathrm{C}} - \pi_{\mathrm{E}\geq\mathrm{C}}^{UL(1)}\right)^2}{\hat{V}_*(\pi_{\mathrm{E}\geq\mathrm{C}}^{UL(1)})}$$

where $\hat{V}_*(\pi_{\mathrm{E}\geq\mathrm{C}}^{UL(0)})$ and $\hat{V}_*(\pi_{\mathrm{E}\geq\mathrm{C}}^{UL(0)})$ are calculated with $\boldsymbol{p}^{\mathrm{E}}$ $(\pi_{\mathrm{E}\geq\mathrm{C}}^{UL(0)})$ and $\boldsymbol{p}^{C}$ $(\pi_{\mathrm{E}\geq\mathrm{C}}^{UL(0)})$, and $\boldsymbol{p}^{\mathrm{E}}$ $(\pi_{\mathrm{E}\geq\mathrm{C}}^{UL(1)})$ and $\boldsymbol{p}^{C}$ $(\pi_{\mathrm{E}\geq\mathrm{C}}^{UL(1)})$), which are estimated by Newton–Raphson algorithm without constrains (Ryu 2007; Ryu and Agresti 2008) or the modified Newton–Raphson algorithm with constraints based on the homogeneous linear predictor models in Lang (2004, 2005). $\hat{V}_*$ is the variance corresponding to Wald-type CI, Halperin et al. (1989)'s CI, and $\tanh^{-1}$ transformation-based CI respectively.

Step 2: Update the value of $\pi_{\mathrm{E}\geq\mathrm{C}}^{UL}$ using the following equation

$$\pi_{\mathrm{E}\geq\mathrm{C}}^{UL(j+1)} = \frac{\pi_{\mathrm{E}\geq\mathrm{C}}^{UL(j-1)}(PS^{UL(j)} - \chi_{1-\alpha}^2(1)) - \pi_{\mathrm{E}\geq\mathrm{C}}^{UL(j)}(PS^{UL(j-1)} - \chi_{1-\alpha}^2(1))}{PS^{UL(j)} - PS^{UL(j-1)}}.$$

where

$$PS^{UL(j-1)} = \frac{\left(\hat{\pi}_{\mathrm{E}\geq\mathrm{C}} - \pi_{\mathrm{E}\geq\mathrm{C}}^{UL(j-1)}\right)^2}{\hat{V}_*(\pi_{\mathrm{E}\geq\mathrm{C}}^{UL(j-1)})} \text{ and } PS^{UL(j)} = \frac{\left(\hat{\pi}_{\mathrm{E}\geq\mathrm{C}} - \pi_{\mathrm{E}\geq\mathrm{C}}^{UL(j)}\right)^2}{\hat{V}_*(\pi_{\mathrm{E}\geq\mathrm{C}}^{UL(j)})}$$

If $\pi_{\mathrm{E}\geq\mathrm{C}}^{UL(j+1)}$ falls outside the range $[\hat{\pi}_{\mathrm{E}\geq\mathrm{C}}, 1]$, the value should be replaced by any value within the range.

Step 3: If $|\pi_{\mathrm{E}\geq\mathrm{C}}^{UL(j+1)} - \pi_{\mathrm{E}\geq\mathrm{C}}^{UL(j)}| < \varepsilon$, then the iteration stops with $\pi_{\mathrm{E}\geq\mathrm{C}}^{UL(j+1)}$ as the final value, where ε is the convergence tolerance. If not, then go back to **Step 2**. If the algorithm does not converge within the maximum allowed iterations, the value that yields the closest calculated statistic function to $\chi_{1-\alpha}^2(1)$ is chosen as the final value.

Lower limit

The lower limit (LL) can be determined in the same manner, with the two initial values $\pi_{\mathrm{E}\geq\mathrm{C}}^{LL(0)}$ and $\pi_{\mathrm{E}\geq\mathrm{C}}^{LL(1)}$ for the lower limit. The value is updated by

$$\pi_{E\geq C}^{LL(j+1)} = \frac{\pi_{E\geq C}^{LL(j-1)}(PS^{LL(j)} - \chi_{1-\alpha}^2(1)) - \pi_{E\geq C}^{LL(j)}(PS^{LL(j-1)} - \chi_{1-\alpha}^2(1))}{PS^{LL(j)} - PS^{LL(j-1)}}$$

with

$$PS^{LL(j-1)} = \frac{\left(\hat{\pi}_{\mathrm{E}\geq\mathrm{C}} - \pi_{\mathrm{E}\geq\mathrm{C}}^{LL(j-1)}\right)^2}{\hat{V}_*(\pi_{\mathrm{E}\geq\mathrm{C}}^{LL(j-1)})} \text{ and } PS^{UL(j)} = \frac{\left(\hat{\pi}_{\mathrm{E}\geq\mathrm{C}} - \pi_{\mathrm{E}\geq\mathrm{C}}^{LL(j)}\right)^2}{\hat{V}_*(\pi_{\mathrm{E}\geq\mathrm{C}}^{LL(j-1)})}.$$

If $\pi_{\mathrm{E}\geq\mathrm{C}}^{LL(j+1)}$ falls outside the range $[0, \hat{\pi}_{\mathrm{E}\geq\mathrm{C}}]$, the value should be replaced by any value within the range.

$$\hat{V}_{\log\varphi^0_{E\geq C}}(\pi^0_{\mathrm{E\geq C}}) \doteqdot \frac{\hat{V}(\pi^0_{\mathrm{E\geq C}})}{\pi^0_{\mathrm{E\geq C}}(1-\pi^0_{\mathrm{E\geq C}})}.$$

For calculating $\hat{V}(\pi^0_{\mathrm{E\geq C}})$ or $\hat{V}_*(\pi^0_{\mathrm{E\geq C}})$, the restricted maximum likelihood estimates of the category proportions $\boldsymbol{P}^{\mathrm{E}}$ and $\boldsymbol{P}^{\mathrm{C}}$ under $\pi_{\mathrm{E\geq C}} = \pi^0_{\mathrm{E\geq C}}$ for all possible $\pi^0_{\mathrm{E\geq C}}$, $\boldsymbol{p}^{\mathrm{E}}\ (\pi^0_{\mathrm{E\geq C}}) = (p_1^{\mathrm{E}}\ (\pi^0_{\mathrm{E\geq C}}), \dots, p_K^{\mathrm{E}}\ (\pi^0_{\mathrm{E\geq C}}))$ and $\boldsymbol{p}^{C}\ (\pi^0_{\mathrm{E\geq C}}) = \left(p_1^{\mathrm{C}}\ (\pi^0_{\mathrm{E\geq C}}), \dots, p_K^{\mathrm{C}}\ (\pi^0_{\mathrm{E\geq C}})\right)$ are required. The modified Newton–Raphson algorithm with constraints based on the homogeneous linear predictor models in Lang (2004, 2005), can be used to find restricted maximum likelihood estimates of category proportions given a value of $\pi^0_{\mathrm{E\geq C}}$. The former is faster, though may produce estimates outside of the permissible range of the proportions. Therefore, the latter method was used for the pseudo-score CIs above.

A two-step iterative procedure is required to find the lower and upper limits of $100(1-\alpha)\%$ pseudo-score type two-sided CIs: First for fixed $\pi^0_{\mathrm{E\geq C}}$, the restricted maximum likelihood estimates of the category proportions, $\boldsymbol{p}^{\mathrm{E}}$ and $\boldsymbol{p}^{\mathrm{C}}$ are obtained by the method in Lang (2004, 2005); then the estimates are plugged into the chi-squared statistic above to evaluate if they satisfy the inequality. In Ryu (2007), the grid search was adapted. Although it is simple and easy to implement, the grid search often takes considerable computing time. We consider a simple linear interpolation algorithm to find the upper and lower limit of the pseudo-score CI shown in Table 3. For the initial values $\pi^{(0)}_{\mathrm{E\geq C}}$ and $\pi^{(1)}_{\mathrm{E\geq C}}$, the lower and upper limits of the Halperin et al. (1989)'s CI will be used for pseudo-score Halperin et al. (1989)'s CI. Similarly, the lower and upper limits of the Wald-type CI and tahn^{-1} transformation-based CI will be used for the pseudo-score Wald-type CI and pseudo-score tahn^{-1} transformation-based CI, respectively. In our experience with real data and simulation, in general, the algorithm completes 3 to 5 iterations to find the limits (Hamasaki et al., 2025).

Similar to the Wald-type CIs, if there is no overlap in the DOOR outcome distribution between groups, or if all patients in both groups fall into only one DOOR category, the pseudo-score statistics cannot be calculated and thus the CI cannot be obtained.

Other CIs

Score test-based and Likelihood ratio (LR) test-based CIs are the alternatives (Ryu and Agresti, 2008). Similar to the pseudo-score CIs described above, these CIs require restricted maximum likelihood estimates of the proportions for each category p_k^g, and the iterative procedure to find the set of $\pi^0_{\mathrm{E\geq C}}$ that satisfies the score statistic or LR statistic, where the score statistic is given by

$$S^2(\pi^0_{\mathrm{E\geq C}}) = \Sigma_{k=1}^K \left[\frac{\left(n_k^{\mathrm{E}} - n^{\mathrm{E}} p_k^{\mathrm{E}}\ (\pi^0_{\mathrm{E\geq C}})\right)^2}{n^{\mathrm{E}} p_k^{\mathrm{E}}\ (\pi^0_{\mathrm{E\geq C}})} + \frac{\left(n_k^{\mathrm{C}} - n^{\mathrm{C}} p_k^{\mathrm{C}}\ (\pi^0_{\mathrm{E\geq C}})\right)^2}{n^{\mathrm{C}} p_k^{\mathrm{C}}\ (\pi^0_{\mathrm{E\geq C}})}\right],$$

and LR statistic for $\mathrm{H}_0\colon \pi_{\mathrm{E\geq C}} = \pi^0_{\mathrm{E\geq C}}$ is given by

$$LR^2(\pi^0_{\mathrm{E\geq C}}) = 2\left(\Sigma_{k=1}^K n_k^{\mathrm{E}}(\log\hat{p}_k^{\mathrm{E}} - \log p_k^{\mathrm{E}}\ (\pi^0_{\mathrm{E\geq C}}) + \Sigma_{k=1}^K n_k^{\mathrm{C}}(\log\hat{p}_k^{\mathrm{C}} - \log p_k^{\mathrm{C}}\ (\pi^0_{\mathrm{E\geq C}})\right).$$

See Ryu and Agresti (2008) for more details. The algorithm shown in Table 3 can be used for score test-based and LR test-based CIs, where the pseudo-score statistics are replaced by score statistic and LR statistic (Ryu and Agresti, 2008), respectively. The calculation of score and LR statistics fails when the maximum likelihood or restricted maximum likelihood estimates of the proportions are zero. Following Ryu and Agresti (2008), cell proportions estimated to be zero are

Table 4. The data generation steps and the performance measure calculation

**Step1**: Generate two sets of independent pseudo random numbers $(p_{1r}^{\mathrm{E}}, \dots, p_{Kr}^{\mathrm{E}})$ and $\left(p_{1r}^{\mathrm{C}}, \dots, p_{Kr}^{\mathrm{C}}\right)$ from multinomial distributions with n^{E} and $(P_1^{\mathrm{E}}, \dots, P_K^{\mathrm{E}})$, and n^{C} and $\left(P_1^{\mathrm{C}}, \dots, P_K^{\mathrm{C}}\right)$, with sizes of $n^{\mathrm{E}} = a^{\mathrm{E}}n$ and $n^{\mathrm{C}} = a^{\mathrm{C}}n$, given category proportions in Table A1 in Online Supplementary Document, where $r = 1, \dots, R$, and R is the number of replications.
**Step 2**: Calculate the two-sided CIs, with the data generated in Step 1, and then evaluate whether each CI includes the true value of $\pi_{\mathrm{E}\geq\mathrm{C}}$.
**Step 3**: Repeat Steps 1 and 2 with R.
**Step 4**: Calculate the coverage probability of each CI.

excluded from the calculation of the Score and LR statistics. Also, similar to the Wald-type CIs, if there is no overlap in the DOOR outcome distribution between groups, or if all patients in both groups fall into only one DOOR category, the score and LR statistics cannot be calculated and thus the CIs cannot be obtained.

The bootstrap percentile CI can be used to construct a CI estimate of the DOOR probability (van Duin et al., 2018), although it requires extensive computational resource. Brunner and Munzel (2000) discussed a generalization of the Behrens-Fisher problem for two samples in a nonparametric setting and suggested that their statistic could be used to construct a CI for the WMW parameter. However, as noted in Ryu and Agresti (2008), their suggested CI is symmetric, centered at $\hat{\pi}_{\mathrm{E}\geq\mathrm{C}}$, and thus shares the same disadvantages of the Wald-type CIs.

Simulation study: Objectives and design

A simulation study was conducted to evaluate the performance of the CI estimation methods for the DOOR probability described in the previous section. Through our investigation, we found that: (1) no improvement in liberalness is achieved by using the exact variance for the Wald-type CI, compared to the first-order approximation of the variance, (2) the arcsine square root transformation-based CI works well but no better than the $\tanh^{-1}$ transformation-based CI, (3) the bootstrap percentile CI is generally liberal and performs better than Wald-type CIs, but fails to produce CIs when the sample size and number of categories are small, and (4) Brunner and Munzel (2000)'s CI is liberal and performs better than Wald-type CIs, but is not necessarily superior to other types of CIs (Ryu and Agresti, 2008). Based on these findings, the following eight CI methods were selected for evaluation in the simulation study.

C1: Wald-type CI (with $\hat{V}$)

C2: Halperin et al. (1989)'s CI

C3: tahn^{-1} transformation-based CI

C4: Pseudo-score Wald-type CI (with $\hat{V}$)

C5: Pseudo-score Halperin et al. (1989)'s CI

C6: Pseudo-score tahn^{-1} transformation-based CI

C7: Score test-based CI

C8: LR test-based CI

The performance measure calculated for the CI methods was the coverage probabilities of the two-sided 95% CI (The lower one-sided 97.5% CI, and the upper one-sided 97.5% CI were also calculated and the result was provided in Appendix). The number of categories, the shape of the DOOR outcome distribution, the sample size, and the sample size distribution were considered as factors affecting performance. The values for each of the factors are as follows.

- The number of categories: $K = 3$, 4, 5, and 9
- The shape of the DOOR outcome distribution: ten scenarios each for the number of categories shown in Table A1 in Appendix
- Total sample size: $n = 20$, 30, 50, 100, 150, 200, 300, 400, and 500
- Sample size allocation: $a^{\mathrm{E}} = 0.5$, 0.7, and 0.8 (i.e., 1:1, 7:3, and 4:1 allocation ratios)

The data generation steps and the performance measure calculation are shown in Table 4. A value of 100,000 was selected for the number of replications R. This number produces a two-sided 95% CI with a width equal to 0.27% when the true proportion is 95%. This was sufficient to evaluate whether the coverage probability was close enough to the nominal level (i.e., 95%), controlling simulation errors appropriately. For the methods that require the iterative procedure to find the lower and upper limit of the CI, the maximum number of iterations and the absolute iteration convergence tolerance are set to 100 and 10^{-4}, respectively, in the simulation. If the iteration did not end with these criteria, the values that minimized the statistic function were treated as if they were the final values.

Simulation study: Results

All simulation results are provided in Figure A1 to Figure A24 in Appendix. Here we summarize only the result for the two-sided CIs with $K = 3$ (the ten scenarios S1 to S10) as there is no major difference in the performance of CIs depending on the number of DOOR categories and whether they are two-sided or one-sided.

In equally-sized groups, Halperin et al. (1989)'s CI (C2), $\tanh^{-1}$ transformation-based CI (C3), pseudo-score Wald-type CI (C4), score test-based CI (C7), and LR test-based CI (C8) maintain good control at the nominal level, even with small sample sizes: the coverage probabilities remain within $\pm 0.1\%$ of the nominal level for sample sizes of $n = 20$, 30, and 50. Pseudo-score Halperin et al. (1989)'s CI (C5) maintains good control at the nominal level, showing a slight conservative tendency in small sample sizes: on average, the coverage probabilities are 0.2%, 0.1%, and <0.1% higher than the nominal level for sample sizes of $n =$ 20, 30, and 50. Wald-type CI (C1) and Pseudo-score $\tanh^{-1}$ transformation-based CI (C6) behave similarly, showing a slight liberal tendency, especially in small sample sizes: On average, for sample sizes of $n = 20$, 30, and 50, the coverage probabilities are 0.4%, 0.2%, and 0.1% lower than the nominal level for C1, and 0.6%, 0.1%, and 0.1% lower for C6. Also, C6 occasionally fails to find the confidence limits, particularly in small sample sizes such as $n = 20$, 30, and 50.

In moderately unequally-sized groups with a 7:3 sample size allocation, similar to those seen in the equally-sized group setting, C2, C3, C4, C7, and C8 maintain still good control at the nominal level, even with small sample sizes: the coverage probabilities remain within $\pm 0.2\%$ of

the nominal level for sample sizes of $n = 20$, 30, and 50; in small sample sizes, C2, C3, C4, and C8 show a slight liberal tendency in small sample sizes, C7 shows a slight conservative tendency. Also C5 maintains good control at the nominal level, exhibiting a slight conservative tendency in small sample sizes, with coverage probabilities remaining within $\pm 0.2\%$ of the nominal level for sample sizes of $n = 20$, 30, and 50. C1 and C6 behave similarly, showing a liberal tendency, especially particularly in small sample sizes: On average, for sample sizes of $n = 20$, 30, and 50, the coverage probabilities are 0.6%, 0.4%, and 0.2% lower than the nominal level for C1, and 1.0%, 0.2%, and 0.2% lower for C6.

In extremely unequal groups with a 4:1 sample size allocation, C7 and C8 maintain still good control at the nominal level, even with small sample sizes: the coverage probabilities remain within $\pm 0.2\%$ of the nominal level for sample sizes of n =20, 30, and 50; in small sample sizes. C2, C3, C4 and C5 also maintains good control at the nominal level, but C2, C3, and C4 show a slight liberal tendency in small sample sizes while C5 exhibits a conservative tendency: for sample sizes of $n = 20$, 30, and 50, on average, the coverage probabilities are 0.4%, 0.2%, and 0.1% lower than the nominal level for C2, 0.6%, 0.3% and 0.2% lower for C3, and 0.9%, <0.1% and <0.1% lower for C4, and 0.6%, 0.3% and 0.2% higher for C5. C1 and C6 behave similarly, showing a liberal tendency, especially particularly in small sample sizes: for sample sizes of $n = 20$, 30, and 50, on average the coverage probabilities are 1.1%, 0.7%, and 0.4% lower than the nominal level for C1, and 2.0%, 0.4%, and 0.1% lower for C6.

The findings from the simulation study are as follows.

- The Wald-type CI does not properly control the coverage probability at the nominal level unless the total sample size is quite large. The coverage probability is below the nominal level.
- The Halperin et al. (1989)'s CI and the $\tanh^{-1}$ transformation-based CI work well in most situations, controlling the coverage probability at the nominal level adequately, even with small sample sizes, except for situations where the total sample size is smaller than 100 and the group sample size is extremely imbalanced (4:1 or greater sample size allocation). In such situations, both coverage probabilities are below the nominal level and thus the CIs are liberal.
- The use of the pseudo-score approach helps to improve the liberalness of the Wald-type CI and Halperin et al. (1989)'s CI. The pseudo-score Wald-type CI work well, controlling the coverage probability at the nominal level even when the sample size is small and the group sample size is extremely imbalanced. The pseudo-score Halperin et al. (1989)'s CI works well although it tends to be conservative with smaller sample sizes. On the other hand, the pseudo-score approach does not help to improve the liberalness of the $\tanh^{-1}$ transformation-based CI; it makes it worse. The coverage probability for the $\tanh^{-1}$ transformation-based CI is smaller than the $\tanh^{-1}$ transformation-based CI. Also, pseudo-score CIs occasionally fail to find the confidence limits, particularly in small sample sizes such as $n = 20$, 30, and 50.
- The score-test CI works well, controlling the coverage probability at the nominal level even when the sample size is small and the group sample size is extremely imbalanced. Also, it occasionally fails to find the confidence limits, particularly in small sample sizes such as $n = 20$, 30, and 50.

- The LR test-based CI works well in most situations, controlling the coverage probability at the nominal level, except for situations where the sample size is small and the group sample size is imbalanced. Also, it occasionally fails to find the confidence limits, particularly in small sample sizes such as $n = 20$, 30, and 50.

2.3 Hypothesis tests

The hypothesis testing for the DOOR probability and respective p-value calculation are reviewed. As the DOOR probability estimate is equivalent to the WMW statistic, it is natural to implement the WMW test to test the DOOR probability.

WMW test

The one-sided hypothesis for the DOOR probability is $\mathrm{H}_0: \pi_{\mathrm{E}\geq\mathrm{C}} \leq \delta_0$ vs. $\mathrm{H}_A: \pi_{\mathrm{E}\geq\mathrm{C}} > \delta_0$ and the two-sided hypothesis is $\mathrm{H}_0: \pi_{\mathrm{E}\geq\mathrm{C}} = \delta_0$ vs. $\mathrm{H}_A: \pi_{\mathrm{E}\geq\mathrm{C}} \neq \delta_0$, where the value of δ_0 is generally chosen as 0.5. Under H_0, the estimate of $\mathrm{var}[\hat{\pi}_{\mathrm{E}\geq\mathrm{C}}]$ is given by

$$\hat{V}_0 = \mathrm{var}[\hat{\pi}_{\mathrm{E}\geq\mathrm{C}}|\mathrm{H}_0] = \frac{n}{12(n-1)a^{\mathrm{E}}a^{\mathrm{C}}}[1 - \Sigma_{k=1}^{K}\bar{p}_k^3],$$

where $\bar{p}_k = a^{\mathrm{E}}p_k^{\mathrm{E}} + a^{\mathrm{C}}p_k^{\mathrm{C}}$ (Lehman 1975). Therefore, the test statistic is given by

$$z_{\mathrm{WMW}} = \frac{\hat{\pi}_{\mathrm{E}\geq\mathrm{C}} - 0.5}{\sqrt{\hat{V}_0/n}}$$

It is well known that z_{DOOR} tends to be distributed as $z_{\mathrm{DOOR}} \sim \mathrm{N}(0,1)$ as n^{E} and n^{C} get large (Lehman 1975). Therefore, the p-value for one-sided or two-sided WMW test can be simply calculated using the normal approximation. The p-values for one-sided and two-sided WMW tests are

$$p_{1\mathrm{sided}}(z_{\mathrm{WMW}}) = \begin{cases} \Pr[Z > z_{\mathrm{WMW}}], & z_{\mathrm{WMW}} > 0, \\ \Pr[Z < z_{\mathrm{WMW}}], & z_{\mathrm{WMW}} < 0, \end{cases}$$

and

$$p_{2\mathrm{sided}}(z_{\mathrm{WMW}}) = \Pr[|Z| > |z_{\mathrm{WMW}}|],$$

respectively. If the p-value is smaller than the prespecified significance level of α (e.g., 5%), we are able to reject H_0.

Continuity correction and t-approximation

Several authors have raised a concern that the normal approximation to the WMW statistic may often be inaccurate when the outcomes are heavily tied. The continuity correction (CC) has been proposed to improve the normal approximation (Lehman, 1975; Klotz, 1966). The p-values from the statistics with CC for one-sided and two-sided WMW are

$$p'_{1\mathrm{sided}}(z_{\mathrm{WMW}}) = \begin{cases} \Pr\left[Z > z_{\mathrm{WMW}} - \dfrac{1}{2n^{\mathrm{E}}n^{\mathrm{C}}\sqrt{\hat{V}_0}}\right], & z_0 > 0, \\ \Pr\left[Z < z_{\mathrm{WMW}} + \dfrac{1}{2n^{\mathrm{E}}n^{\mathrm{C}}\sqrt{\hat{V}_0}}\right], & z_0 < 0, \end{cases}$$

and

$$p'_{\text{2sided}}(z_{\text{WMW}}) = \Pr\left[|Z| > |z_{\text{WMW}}| - \frac{1}{2n^{\text{E}}n^{\text{C}}\sqrt{\hat{V}_0}}\right],$$

respectively. Instead of the normal approximation, some statistical analysis software has a function to implement the t-approximation for the p-value calculation, where the WMW statistic z_{DOOR} is assumed to follow t-distribution with $n-1$ degrees of freedom. For example, PROC NPAR1WAY in SAS® Version 9.4 uses CC by default for both normal and t-approximations.

Transformation-based methods

Similar to the CI estimates discussed in Section 2.2, the $\tanh^{-1}$transformation-based method can be simply used to test hypotheses about the DOOR probability. The one-sided hypothesis is H_0: $\log\varphi_{\text{E}\geq\text{C}} \leq 0$ vs H_A: $\log\varphi_{\text{E}\geq\text{C}} > 0$, and the two-sided hypothesis is H_0: $\log\varphi_{\text{E}\geq\text{C}} = 0$ vs. H_A: $\log\varphi_{\text{E}\geq\text{C}} \neq 0$, where $\varphi_{\text{E}\geq\text{C}} = \pi_{\text{E}\geq\text{C}}/(1-\pi_{\text{E}\geq\text{C}})$. Using the variance approximation to $\mathbb{V}\text{ar}[\log\hat{\varphi}_{\text{E}\geq\text{C}}]$ described in Section 2, under the null, the $\tanh^{-1}$ transformation-based statistic is

$$z_{\tanh^{-1}} = \frac{\log\hat{\varphi}_{\text{E}\geq\text{C}}}{4\sqrt{\hat{V}_0/n}}.$$

O'Brien and Castelloe (2006) discussed the power and sample size calculation based on $\log\varphi_{\text{E}\geq\text{C}}$ and the method has been implemented in PROC POWER in SAS ® Version 9.4. Although their statistic form is the same as above, $\hat{V}_0$ is calculated by $\hat{V}$ with $p_k^{\text{E}} = p_k^{\text{C}} = \bar{p}_k$. Therefore, the statistic is

$$z_{\text{OC}} = \frac{\log\hat{\varphi}_{\text{E}\geq\text{C}}}{4\sqrt{\hat{V}(\bar{p}_k)/n}}.$$

Similar to the WMW test above, the p-value for one-sided or two-sided test based on these statistics can be calculated using the normal approximation.

Simulation study: Objectives and design

A simulation study was conducted to evaluate the performance of the following six tests for the DOOR probability.

T1: WMW test/the normal approximation without CC

T2: WMW test/the normal approximation with CC

T3 WMW test/the t-approximation without CC

T4: WMW test/the t-approximation with CC

T5: $\tanh^{-1}$ transformation-based test

T6: O'Brien and Castelloe (2006)'s test

The performance measure calculated for the tests methods was the actual size (the Type I error probability) of the two-sided test at 5% significance level. Also, the upper size of one-sided test at 2.5% significance level were calculated. Similar to the CI method evaluation, the number of categories, the shape of the DOOR outcome distribution, the sample size, and the sample size distribution were considered as factors potentially affecting the performance. The values for each of the factors and the data generation steps are the same as in the CI method evaluation shown in

Table 4 except for the shape of the DOOR outcome distribution. To evaluate the size of test, for the shape of the DOOR outcome distribution, the four null hypothesis scenarios that the DOOR outcome distribution is identical between groups, were considered, as shown in Table A1 in Appendix (i.e., S1-4, S11-14, S21-S24, and S31-S34). For the assessment of the size of test, the number of replicates was chosen to be 1,000,000, which produces a two-sided 95% CI with a width of 0.09% when the true proportion is 5%.

Simulation study: Results

All simulation results are summarized in Figure A25 to Figure A32. Here we mainly summarize the result for the two-sided tests with $K = 3$ as there is no major difference in the tendency of the performance of the tests depending on the number of DOOR categories and whether they are two-sided or one-sided.

Regardless of whether the group size is equal or unequal, in four scenarios, the size of the test for the WMW test using normal approximation without CC (T1) and with CC (T2) behave similarly especially when the total sample size is larger than 150, although the size of test for T2 is always slightly smaller than that for T1. Both sizes of test are around the nominal level, except when the total sample size is less than 100 to 150. When the sample size is less than 100 to 150, their sizes of the test are slightly below the nominal level. For example, $n = 50$, on average, the size of test is 0.12% lower than the nominal level for T1 and 0.24% lower for T2.

The size of the test for the WMW test using t-approximation without CC (T3) and with CC (T4) behave similarly, especially when the total sample size is less than 100 to 150, although the size of test for T4 is always slightly smaller than that for T3. Both sizes of test are mostly below the nominal level, especially when the sample size is small. For example, $n = 50$, on average, the size of test is 0.65% lower than the nominal level for T3 and 0.79% lower for T4.

The size of the test for the $\tanh^{-1}$ transformation-based test (T5) and the O'Brien and Castelloe (2006)'s test (T6) behaves similarly, always above the nominal level. Especially when the sample size is small, this behavior is severe. For example, $n = 50$, on average, the size of test is 0.75% larger than the nominal level for T3 and 0.96% larger for T4.

The findings from the simulation study are as follows.

- The WMW test using the normal approximation without CC works well, controlling the size of test at the nominal level adequately, except when the total sample size is smaller than 100 to 150. When the total sample size is smaller than 100 to 150, the size is slightly below the nominal level and thus the test is slightly conservative.
- The WMW test using the normal approximation with CC behaves similarly to the WMW test using the normal approximation without CC, especially when the total sample size is larger than 150, there is no major difference in the size of test between the two tests. The size of test for The WMW test using the normal approximation with CC is always smaller than that for the WMW test using the normal approximation without CC, and thus the WMW test using the normal approximation with CC is more conservative than the WMW test using the normal approximation without CC. The WMW test using the t-approximation without CC or with CC, behaves similarly. Their sizes of test are always below the nominal level, and smaller than those for the WMW test using the normal

approximation with/without CC. From above, use of CC and/or t-approximation makes WMW test just conservative.

- The $\tanh^{-1}$ transformation-based test and the O'Brien and Castelloe (2006)'s test cannot control the size of test at the nominal level adequately, even with large sample sizes. Their sizes of test are above the nominal level.

2.4 Recommendations

Table 5 summarizes the strengths and limitations of the eight CI estimation methods. Given the results of the simulation and numerical examples, the guidelines for using the CI estimation methods are as follows.

- We recommend using the Halperin et al. (1989)'s CI because: (1) it works well to control the confidence level at the nominal level, (2) it is easy to implement as it has a closed form for the calculation that does not require an iterative procedure to find the upper and lower limits, and (3) the lower and upper limits are always available even if the other CIs that require an iterative procedure fail to find them. On the other hand, the Halperin et al. (1989)'s CI is liberal when the total sample size is smaller than 300 and the group sample size is extremely imbalanced (4:1 or greater sample size allocation) although this is a very uncommon situation in clinical trials. In such a situation, to minimize the risk of incorrectly inferring a statistically significant result when using the CI instead of the hypothesis test, alternatively the pseudo-score Halperin et al. (1989)'s CI is recommended although this is slightly conservative.
- Alternatively, the tahn^{-1} transformation-based CI may be used as it has the same the strengths as the Halperin et al. (1989)'s CI. However, similar to the Halperin et al. (1989)'s CI, it is liberal when the total sample size is smaller than 300 and the group sample size is extremely imbalanced. Unlike the Halperin et al. (1989)'s CI, the pseudo-score approach does not help to improve the liberalness of the tahn^{-1} transformation-based CI.
- The score test-based CI and the pseudo score Wald-type CI may be another alternative as they work well to control the confidence level at the nominal level even when the total sample size is smaller than 300 and the group sample size is extremely imbalanced. However, their calculations are more complicated than the CI's of Halperin et al. (1989). Both require an iterative procedure to find the limits and often fail to find them in small, imbalanced group sample sizes.
- We do not recommend using the Wald-type CIs for the DOOR probability: the Wald-type CIs are liberal and do not adequately control the coverage probability at the nominal level, even with large sample sizes. We do not recommend using the LR-test CI for the DOOR probability: the LR test-based CI is liberal in general, and the lower and upper limits cannot often be found.

The guidelines for using the hypothesis test methods are as follows.

- We recommend using the WMW test using the normal approximation without CC since it simply works well to control the size of test at the nominal level in practical situations although it tends to be slightly conservative when the total sample size is smaller than 100 to 150.

Table 5. Strengths and limitations of CI methods

CI Method	Strength	Limitation
Wald	• Has a closed form to calculate the CI estimate and is easy to use	• Produces a liberal CI generally; does not control the coverage probability at the nominal level; the coverage probability is always below the nominal level, even with large sample sizes<br>• Estimates of the lower or upper bound of the DOOR probability may be outside the DOOR probability range of 0 to 100%, with small sample sizes and/or when the DOOR probability is close to 0 or 100%<br>• Fails to estimate the lower or upper bound of the DOOR probability if there is no overlap in the DOOR outcome distribution between groups, or if all patients in both groups fall into only one DOOR category
Halperin et al. (1989)	• Has a closed form to calculate the CI estimate and is easy to use<br>• Estimates of the lower or upper bound of the DOOR probability are within 0-100%<br>• Controls the coverage probability at the nominal level well in most practical situations, even with small sample sizes, except for extremely imbalanced group sizes	• Tends to be liberal with small, extremely imbalanced group sizes
$\tanh^{-1}$ transformation	• Has a closed form to calculate the CI estimate and is easy to use<br>• Estimates of the lower or upper bound of the DOOR probability are within 0-100%<br>• Controls the coverage probability at the nominal level well in most practical situations, even with small sample sizes, except for extremely imbalanced group sizes	• Tends to be liberal with small, extremely imbalanced group sizes<br>• Fails to estimate the lower or upper bound of the DOOR probability if there is no overlap in the DOOR outcome distribution between groups, or if all patients in both groups fall into only one DOOR category

CI Method	Strength	Limitation
Pseudo-score Wald	• Estimates of the lower or upper bound of the DOOR probability are within 0-100%<br>• Controls the coverage probability at the nominal level even in small, imbalanced group sizes	• Has no closed form to estimate the CI<br>• Requires an iterative procedure to estimate the CI
Pseudo-score Halperin et al. (1989)	• Estimates of the lower or upper bound of the DOOR probability are within 0-100%<br>• Controls the coverage probability at the nominal level except for small sample sizes	• Has no closed form to estimate the CI<br>• Requires an iterative procedure to estimate the CI<br>• Produces a conservative CI generally when the sample size is small
Pseudo-score $\tanh^{-1}$ transformation	• Estimates of the lower or upper bound of the DOOR probability are within 0-100%<br>• Controls the coverage probability at the nominal level well in most practical situations, even with small sample sizes, except for extremely imbalanced group sizes	• Has no closed form to estimate the CI<br>• Requires an iterative procedure to estimate the CI<br>• Tends to be liberal with small, extremely imbalanced group sizes
Score test	• Estimates of the lower or upper bound of the DOOR probability are within 0-100%<br>• Controls the coverage probability at the nominal level even in small, imbalanced group sizes	• Has no closed form to estimate the CI<br>• Requires an iterative procedure to estimate the CI
LR test	• Estimates of the lower or upper bound of the DOOR probability are within 0-100%<br>• Controls the coverage probability at the nominal level well in most practical situations, even with small sample sizes, except for extremely imbalanced group sizes	• Has no closed form to estimate the CI<br>• Requires an iterative procedure to estimate the CI<br>• Tends to be liberal with small, extremely imbalanced group sizes

- We do not recommend the use of CC, t-approximation, or their combination by default. They make the WMW test more conservative, i.e., the WMW test is less powerful and

requires a larger sample size to detect an important difference in the benefit and risk of interventions.

- We don't recommend using either of the $\tanh^{-1}$ transformation-based test or the O'Brien and Castelloe (2006)'s test. Both cannot control the size of test and their sizes of test are inflated above the nominal level even with large sample sizes. The inflation in both tests is caused by the biased estimate of $\log\varphi_{\mathrm{E\geq C}}$. The expectation of $\log\hat{\varphi}_{\mathrm{E\geq C}}$ is approximately given by

$$\mathbb{E}[\log\hat{\varphi}_{\mathrm{E\geq C}}] \fallingdotseq \log\varphi_{\mathrm{E\geq C}} + \frac{(2\pi_{\mathrm{E\geq C}} - 1)V}{2\pi_{\mathrm{E\geq C}}(1-\pi_{\mathrm{E\geq C}})^2}$$

where the second term is the bias to test $\mathrm{H}_0: \log\varphi_{\mathrm{E\geq C}}$. The effect of this bias on the tests becomes smaller as the DOOR probability is closer to 50% and/or the sample size is larger. The bias correction will be required when using the $\tanh^{-1}$ transformation-based test and the O'Brien and Castelloe (2006)'s test.

3. GRADE-BASED ANALYSES: PARTIAL CREDITS

The DOOR probability-based method discussed in Section 2 has several advantages. One major advantage is that, unlike the alternative, it does not rely on distributional or other assumptions. However, a potential concern is that the DOOR probability may not provide the appropriate amount of influence to each specific rank category. When calculating the DOOR probability, in all possible pairwise comparisons of patients' DOOR outcomes between the test and control intervention groups, an individual pairwise comparison is labeled a "more desirable", "less desirable", or "tie", depending on whether the experimental group patient had a more desirable, less desirable, or tied DOOR outcome than the control group patient, regardless of clinical importance of each category (e.g., a more desirable outcome of "Alive with no non-fatal SAEs" vs. "Alive with non-fatal SAEs" is treated the same as a more desirable outcome of "Alive with no non-fatal SAEs" vs. "Death").

To address this concern, the partial credit analysis has been proposed (Evans and Follmann, 2016; Evans, 2022). The partial credit analysis assigns the relative importance to each category directly. For example, there is a DOOR outcome consisting of the three categories, Alive with no non-fatal SAEs (most desirable), Alive with at least one non-fatal SAEs, and Death (least desirable). Envision scoring the three categories of the ordinal outcome as if one were scoring an academic test. If a patient is alive without non-fatal SAEs, then a score of 100% is assigned. If the patient dies, then a score of zero is assigned. If the patient survives with a major AE, then partial credit is given, such as a score of 60, which ranges from 0 to 100. Once all scores have been assigned, the analyses consist of estimating the between-group difference in mean scores on a 100-point scale, using t-statistic-based methods, as if the outcome were continuous. Given assigned grades, the DOOR probability-based analysis can be also implemented. Assigning the same partial credit grade to different adjacent rank categories will result in the combination of those categories to be combined into a single category in the rank-based method.

The natural question is how to determine partial credit. Partial credit may be informed from: (1) patients using quality-of-life instruments, or (2) from expert clinicians using surveys. Treatment comparisons are made by comparing mean partial credit scores between treatments.

Table 6. Guiding principles for maximizing replicability, robustness, objectivity, transparency, and pragmatism

• Analyze the patient story (patient-centricity)
• Robustness: avoid/minimize reliance upon assumptions e.g., proportional odds, prior distribution of treatment effects, specification of a model form, for analysis validity
• Objectivity: free from investigator beliefs
• Clearly defined estimands and populations from which to estimate parameters
• Prioritize error control consistent with the evidentiary standard for confirmatory evidence
➢ Unbiased estimates of treatment effects
➢ Correct coverage probability for confidence interval estimation
• Implement best practices for composite endpoints including integrated analyses of components
• Implement best practices for multi-outcome benefit:risk analyses to aid comprehensive assessment
➢ Analyses based on the absolute (vs. relative) risk scale consistent with providing a common scale for interpretation of multiple outcomes simultaneously
• Evaluate robustness of grade-based analyses
• Recognize rank-based and grade-based dimensions of treatment contrast
• Implement best practices for ordinal outcomes including cumulative analyses
• Intuitive interpretation e.g., DOOR probability (vs. odds ratio)
• Care with technical fundamentals including appropriate handling of ties for rank-based analyses utilizing pair-wise comparisons

The advantages of the partial credit analysis are that it strategically scores the DOOR categories to account for non-uniform steps between categories and it has an intuitive interpretation given the 100-point scale. Partial credit grading can be pre-specified during clinical trial design for transparency and serve as a foundation for statistical error control. During analyses the treatment contrast can be displayed as partial credit assignment varies allowing (1) for thorough robustness analyses, and (2) researchers, clinicians, and patients the freedom to evaluate the interventions' effects based on personalized perspectives regarding the desirability of the DOOR categories. This allows them to see how the outcome changes on their own preference and helps them choose a treatment for individual patient. Plotting the differences in mean partial credit between interventions against the corresponding DOOR probabilities provides a good visual tool for evaluating (1) and (2).

4. GUIDING PRINCIPLES, RECOMMENDED STATISTICAL ANALYSIS PLAN, AND ONLINE DOOR ANALYSIS TOOLS

DOOR analyses are shaped by guiding principles for maximizing replicability, robustness, and rigor shown in Table 6. The recommended statistical analysis plan (SAP) for DOOR and a

Table 7. Recommended statistical analysis plan and associated DOOR apps output

- Table: DOOR Outcome Distribution by Treatment
  - The table includes a standardized expected gain/loss summary. The expected DOOR distribution for the control therapy standardized per a specified number of user-defined assigned patients is shown. The summary provides the number of patients that would be gained or lost in each DOOR level if treated with the investigational therapy relative to the control. The cumulative gain/loss is provided.
- Table: Distribution of Component Outcomes by Treatment
- Figure: Bar Chart of DOOR Outcome Distribution by Treatment
- Figure: Bar Chart of Component Outcomes Distributions by Treatment
- Figure: Forest Plot of Confidence Interval Estimates for the DOOR Probability for the DOOR Outcome and Respective Components
  - The DOOR outcome is a composite. A fundamental tenet of analyses of composites is incorporation of analysis of each of the components to ensure a thorough understanding of the manifestation of effects, e.g., whether effects on components go in similar or opposing directions, or if there are specific components driving the observed effect on the composite. Comprehensive analyses include estimation of the DOOR probability for individual components such as mortality or the occurrence of an SAE, using the same methods. An advantage of the DOOR probability metric is that it is an absolute metric that provides for a common scale for all outcomes, allowing for simultaneous interpretation of multiple outcomes, in contrast to relative measures that do not have a common scale due to different baseline rates. Resulting analyses includes confidence interval estimates of a more desirable result with respect to the DOOR outcome and each of its components.
  - The app includes options to prioritize efficacy or safety components when estimating the DOOR probability by using pairwise comparisons of surviving patients with events.
- Figure: Forest Plot of the Confidence Interval Estimates for the Cumulative DOOR Probability Based on Sequential Dichotomization of the DOOR Outcome
- Table: Partial Credit Analyses Summary for Selected Grading Keys
- Figure: Plot of the Difference in Means vs. DOOR Probability Based upon Selected Grading Keys
  - The plot includes vertical and horizontal lines for each bivariate estimate displaying grading key-specific confidence interval estimates for the difference in means and the DOOR probability.
- Figure: Anthology of Patient Stories
  - Clarification: The plot displays the percentage of patients that experience each possible combination of component outcomes by treatment.
- Figure: For DOOR outcomes with three levels, a plot of the difference in means and associated point-wise confidence band, as the partial credit assigned to the middle DOOR outcome category varies from 0-100%, is provided. For DOOR outcomes with four levels, a contour plot of the difference in means as the partial credit assigned to the middle two DOOR outcome categories vary from 0-100%, is provided. Color provides information regarding statistical significance e.g., areas shaded in green identify grading keys that result in p-values less than 0.05

recommended analyses are available. A summary of app output based on the recommended SAP for DOOR is displayed in Table 7. Resulting tables and figures can be saved for manuscripts, presentations, and reports.

Two app editions are available for the analyses. The standard edition requires summary level data input, i.e., a summary table of the DOOR outcome distributions (frequencies or proportions), and DOOR component outcomes by the two intervention groups. The app generates the recommended components of comprehensive DOOR analyses, including distribution summary of DOOR outcome and respective components, forest plot displays of the estimates of the DOOR probabilities for the DOOR outcome and respective components, and partial credit analysis. It is important to have a better understanding of how each component and each DOOR category may contribute to the overall composite outcome as the DOOR outcome is a composite ordinal endpoint. One forest plot summarizes the treatment effect on DOOR and its components together using the same contrast measure, such as the DOOR probability with corresponding CIs. Another forest plot characterizes how the two DOOR outcome distributions differ, by plotting the difference between the two cumulative DOOR outcome distributions using DOOR probabilities.

The professional edition requires individual-level data input. In addition to the analyses provided in the standard edition, the professional edition includes additional functionalities and informed visualizations, including the anthology of patient stories (APS) plot, inverse probability weighted (IPW) analysis, and tie-breaker analyses. The APS plot can visually tell a story of what events each patient experienced and how each patient's outcome contributes to the overall DOOR. The IPW analysis can be used to account for missing data in clinical trials and biases in observational studies. Although not discussed in this chapter, once the weights are calculated and then the weighted estimates of category proportions are obtained, the IPW analysis can be performed simply using the methods discussed in the previous sections. Tie-breaker analyses adjust for differences of patients in the same DOOR category level by stratifying patients into new levels based on a lower priority outcome.

The apps provide comprehensive tools for clinical researchers to implement the DOOR methodology for their studies. Both editions provide user functions to customize labels and parameters for the analyses. Analyses output can be saved, with tables in csv format and figures in png format. The feature of each edition is summarized in Table A2 in Appendix.

5. ILLUSTRATIONS WITH CLINICAL STUDIES

In this section, we illustrate the statistical analyses for DOOR outcomes described in Section 4 with three clinical studies.

5.1 DORI-05

DORI-05 was a randomized double-blind clinical trial that evaluated whether intravenous (IV) administration of doripenem, a carbapenem with bactericidal activity against gram-negative and gram-positive uropathogens, was inferior to IV administration of levofloxacin in patients with cUTI (Naber et al., 2009). Analyses are illustrated using a DOOR outcome developed by the ARLG (Howard-Anderson et al., 2023). The DOOR outcome incorporates mortality and non-fatal deleterious events: absence of clinical success, non-fatal SAEs (including *Clostridium difficile*), and infectious complications. This results in a DOOR outcome with five ranked

Table 8. The DOOR outcome for cUTI

Desirability	DOOR outcome category
Most desirable	Alive with no events (DOOR1)
	Alive with 1 event (DOOR2)
	Alive with 2 events (DOOR3)
	Alive with 3 events (DOOR4)
Least desirable	Death (DOOR5)

categories that recognizes death as the worst outcome and distinct from other deleterious events, and the cumulative nature of the non-fatal deleterious events shown in Table 8. We display the recommended DOOR analyses (except for the APS plot), parts of which were described in Howard-Anderson et al. (2023), using the standard edition of the app.

Table A3 and Figure A33 in Appendix display the distribution of DOOR outcome and respective components by intervention group. Doripenem had 70.3% of patients with the most desirable result (alive with none of the three events) vs. 67.6% for levofloxacin. One patient died in the doripenem group vs. 0 for levofloxacin. If 1,000 patients were assigned to be treated with doripenem instead of assigning them to be treated with levofloxacin, there would comparatively be an expected gain of 27 patients as alive with zero events, 86 fewer clinical failures, 48 more infectious complications, 29 more non-fatal SAEs, and 3 more deaths.

Figure A34 in Appendix displays the difference of DOOR outcome and respective components between the two intervention groups. The DOOR probability for doripenem for the DOOR outcome is estimated 51.0% (95% CI: 47.6%, 54.3%), counterbalancing a benefit:risk tradeoff with respect to the absence of clinical success (DOOR probability estimate: 54.3% (95% CI: 51.1%, 57.4%)) and infectious complications (DOOR probability estimate: 47.6% (95% CI: 46.2%, 49.0%)). The estimate of the cumulative probability of DOOR, based on sequential dichotomization of the DOOR outcome is 51.3%, favoring doripenem over levofloxacin in (DOOR 1, DOORs 2 to 5), but it is 48.9% favoring levofloxacin over doripenem in (DOORs 1 and 2, DOORs 3 to 5).

Table A4, Figure A35, and Figure A36 in Appendix present partial credit analyses for four grading keys. These grading keys were selected strategically for illustration purposes. Grading Key A (100;100;100;100;0) defines a binary survival endpoint (alive or dead) and assigns full credit for survival (DOORs 1 to 4) and no credit for mortality (DOOR 5), regardless of the number of events experienced by patients. This grading key results in a difference of −0.3 (95% CI: −0.8, 0.3; P = 0.32). Grading Key B (100;100;100;0;0), representing a binary endpoint of "alive with two or fewer events" or "alive with more than two events or death", assigns full credit for survival with two or fewer events (DOORs 1 to 3) and no credit for survival with more than two events or death (DOORs 4 and 5). This key results in a difference of −0.2 (95% CI: −1.2, 0.6; P = 0.56). Grading Key C (100;100;0;0;0), representing a binary endpoint of "alive with no or one events" or "alive with more than one event or death", assigns full credit for survival with no events or one event (DOORs 1 and 2), and no credit for survival with more than one event or death (DOORs 3 to 5). This grading key results in a difference of −2.1 (95% CI: −4.9, 0.6; P = 0.12). Grading Key D (100;0;0;0;0), equivalent to a binary clinical success endpoint of "alive with no events" or "alive with at least one event or death", assigns full credit for survival with no events (DOOR 1) and no credit for alive with at least one event or death

Table 9. The DOOR outcome for COVID-19

Desirability	DOOR outcome category
Most desirable	Alive with no events (DOOR1)
	Alive with 1 event (DOOR2)
	Alive with 2 events (DOOR3)
Least desirable	Death (DOOR4)

(DOORs 2 to 5). This grading key results in a difference of 2.7 (95% CI: −4.0, 9.3; P = 0.43). The varying results across grading keys demonstrate that the choice of key influences the outcome. Specifically, while Grading Key D favors doripenem over levofloxacin in both partial credit and DOOR probability-based analyses (The DOOR probability estimate: 51.3% (95% CI: 48.0%, 54.6%)), Grading Key C favors levofloxacin over doripenem in both analyses (The DOOR probability estimate: 48.9% (95% CI: 47.5%, 50.3%)). These findings suggest that the preference between doripenem and levofloxacin may depend on individual perspectives regarding the desirability of the DOOR categories.

5.2 ACTT-1

The Adaptive Covid-19 Treatment Trial (ACTT-1) was a randomized, double-blind, placebo-controlled trial of intravenous remdesivir in adults who were hospitalized with Covid-19 and had evidence of lower respiratory tract infection. Patients were randomly assigned to receive remdesivir or placebo for up to 10 days (Beigel et al., 2020).

We illustrate post-hoc DOOR analyses (except for the APS plot) from ACTT-1 with a DOOR outcome developed by trial investigators, using the standard edition of the app. The DOOR outcome incorporates mortality and non-fatal deleterious events: hospitalization with invasive mechanical ventilation (IMV) /extracorporeal membrane oxygenation (ECMO), and non-fatal SAEs. This results in a DOOR outcome with four ranked categories that recognize death as the worst outcome, and the cumulative nature of the non-fatal deleterious events shown in Table 9.

Table A5 and Figure A37 display the distribution of the DOOR outcome and respective components by treatment. Remdesivir had 80.0% of patients with the most desirable result (alive with no events) vs. 73.3% for placebo. Placebo at 14.6% of patients with the least desirable outcome of death vs. 10.7% for remdesivir. If 1,000 patients were assigned to be treated with remdesivir instead of assigning them to placebo, there would comparatively be an expected gain of 67 patients as alive with zero events, 22 fewer hospitalizations with IMV/ECMO, 5 fewer non-fatal SAEs, and 39 fewer deaths.

Figure A38 displays the difference of DOOR outcome and respective components between the two intervention groups. The DOOR probability for remdesivir for the DOOR outcome is estimated 53.3% (95% CI: 50.8%, 55.9%) indicating an overall remdesivir benefit. The DOOR probability for the hospitalization with IMV/ECMO is estimated 51.1% (95% CI: 49.4%, 52.9%), for non-fatal SAEs is 50.2% (95% CI: 49.3%, 51.1%), and for death is 51.9% (95% CI: 49.9%, 53.9%). The estimates of the cumulative probability of DOOR based on sequential dichotomization of the DOOR outcome are greater than 50%, consistently favoring remdesivir over placebo in all sequential dichotomization comparisons.

Table 10. The DOOR outcome for CRE infections

Desirability	DOOR outcome category
Most desirable	Alive with no events (DOOR1)
	Alive with 1 event (DOOR2)
	Alive with 2 events (DOOR3)
Least desirable	Death (DOOR4)

Table A6, Figure A39, and Figure A40 present partial credit analyses for four grading keys. As in the first example (DORI-05), the grading keys were strategically selected for illustration. Grading Key A (100;100;100;0) defines a binary survival endpoint (alive or dead) and assigns full credit for survival (DOORs 1 to 3) and no credit for mortality (DOOR 4), regardless of the number of non-fatal deleterious events experienced by patients. This grading key results in a difference of 3.9 (95% CI: −0.1, 7.9; P = 0.06). Grading Key B (100;0;0;0), equivalent to a binary clinical success endpoint of "alive with no non-fatal deleterious events" or "alive with at least one non-fatal deleterious event or death", assigns full credit for survival with no events (DOOR 1) and no credit for alive with at least one event or death (DOORs 2 to 4). This grading key results in a difference of 6.7 (95% CI: 1.6, 11.8; P = 0.01). Grading Key C (100;100;0;0), equivalent to a binary endpoint of "alive with zero or one non-fatal deleterious event" or "alive with two non-fatal deleterious events or death", assigns full credit for survival with no events or one event (DOORs 1 and 2) and no credit for alive with at least one event or death (DOORs 3 to 4). This grading key results in a difference of 3.5 (95% CI: −0.6, 7.7; P = 0.10). Grading Key D (100;80;60;0) represents a layered compromise, assigning 80% credit for DOOR outcome level two (alive with one deleterious event) and 60% credit for DOOR outcome level three (alive with both deleterious events), resulting in a difference of 4.3 (95% CI: 0.4, 8.4; P = 0.03). Different keys yielded the same result, favoring remdesivir over placebo in both the partial credit and DOOR probability-based analyses (the DOOR probability estimate ranges from 51.8% to 53.4% with these grading keys, favoring remdesivir over placebo). Figure A41 displays a contour plot of the between-group difference in means as the partial credits assigned to DOOR 2 and DOOR 3 vary. Green areas indicate grading key combinations that result in p-values less than 0.05, and positive numbers favor remdesivir and negative numbers favor placebo. The figure shows a result favoring remdesivir over placebo.

5.3 CRACKLE

CRACKLE is a prospective, multicenter, observational study, where patients initially treated with either ceftazidime-avibactam or colistin for Carbapenem-resistant *Enterobacterales* (CRE) infections were selected from the Consortium on Resistance Against Carbapenems in *Klebsiella* and Other *Enterobacteriaceae* (CRACKLE) (van Duin et al., 2018), and the benefit:risk of ceftazidime-avibactam first vs. colistin first was evaluated using the DOOR methodology, where the DOOR outcome consisted of the four ordinal categories shown in Table 10.

We conduct the recommended DOOR analyses, part of which was displayed in van Duin et al. (2018), using the professional edition of the app, highlighting the IPW-based analyses compared to naïve (unweighted) analyses. The propensity score was calculated using the baseline Pitt score and type of infection (van Duin et al., 2018). Note that the analyses in van

Duin et al. (2018) used the percentile bootstrap method for the CI estimation of the DOOR probability, while the app utilizes the method described by Halperin et al. (1989).

Table A7, Figure A42, and Figure A43 display the unadjusted and IPW distribution of the DOOR outcome and respective components by treatment. In the adjusted analysis, ceftazidime-avibactam first had 23.1% of patients with the most desirable result (discharged home) vs. 8.7% for colistin first. Colistin first at 26.1% of patients with the least desirable outcome of hospital death vs. 7.7% for ceftazidime-avibactam first. In the IPW analysis, ceftazidime-avibactam first had 20.5% of patients with the most desirable result (discharged home) vs. 7.8% for colistin first. Colistin first at 25.0% of patients with the least desirable outcome of hospital death vs. 9.1% for ceftazidime-avibactam first. Figure A44 displays an anthology of patient stories (APS) plot the percentage of patients that experience each possible combination of component outcomes by treatment.

Figure A45 and Figure A46 display the adjusted and IPW difference of DOOR outcome and respective components between the two intervention groups. In the naïve analysis, the DOOR probability for ceftazidime-avibactam first is 66.2% (95%CI: 54.4%, 76.3%), indicating an overall benefit of ceftazidime-avibactam first over colistin first. Similar to the naïve analysis, the IPW analysis also shows an overall benefit of ceftazidime-avibactam first with a DOOR probability of 64.3% (95% CI: 52.4%, 74.7%). In both naïve and IPW analyses, the estimates of the cumulative probability of DOOR based on sequential dichotomization of the DOOR outcome are greater than 50%, consistently favoring ceftazidime-avibactam first over colistin first in all sequential dichotomization comparisons.

Table A8, Figure A47, Figure A48, Figure A49, and Figure A50 present unadjusted and IPW partial credit analyses for four grading keys, which were the same in ACTT-1. For the unadjusted analysis, Grading Key A (100;100;100;0) defines a binary survival endpoint (alive or dead) and assigns full credit for survival (DOORs 1 to 3) and no credit for mortality (DOOR 4). This grading key results in a difference of 18.4 (95% CI: 1.6, 35.2; P = 0.03). Grading Key B (100;0;0;0), equivalent to a binary clinical success endpoint of “discharged home” or “alive but not discharged home or death”, assigns full credit for discharged home (DOOR 1) and no credit for alive but discharged not to home with/without renal failure incident or death (DOORs 2 to 4). This grading key results in a difference of 14.4 (95% CI: -4.7, 33.4; P = 0.14). Grading Key C (100;100;0;0), equivalent to a binary endpoint of “alive with no renal failure incident” or “alive with renal failure incident or death”, assigns full credit for alive with no renal failure incident (DOORs 1 and 2) and no credit for alive with renal failure incident or death (DOORs 3 and 4). This grading key results in a difference of 25.5 (95% CI: 6.2, 44.6; P = 0.01). Grading Key D (100;80;60;0) represents a layered compromise, assigning 80% credit for DOOR 2 (alive in hospital or discharged not to home, no renal failure incident) and 60% credit for DOOR 3 (Alive in hospital or discharged not to home, renal failure incident), resulting in a difference of 19.0 (95% CI: 4.5, 33.5; P = 0.01). Different keys yielded the same result, favoring ceftazidime-avibactam first over colistin first in both the partial credit and DOOR probability-based analyses (the DOOR probability estimate ranges from 57.2% to 66.2% with these grading keys, favoring ceftazidime-avibactam first over colistin first). The IPW analysis similarly favored ceftazidime-avibactam first over colistin first: 15.9 (-1.3, 33.2; P = 0.07) for Grading Key A; 12.6 (-5.6, 30.9; P = 0.17) for Grading Key B; 22.1 (2.2, 42.1; P = 0.03) for Grading Key C; 16.5 (1.8, 31.2; P =

0.03) for Grading Key D; the DOOR probability estimates range from 56.3% to 64.3% with these grading keys.

Figure A51 and Figure A52 display a contour plot of the unadjusted and IPW between-group difference in means as the partial credits assigned to DOOR 2 and DOOR 3 vary. Green areas indicate grading key combinations that result in p-values less than 0.05, and positive numbers ceftazidime-avibactam first and negative numbers favor colistin first. The figures show a result favoring ceftazidime-avibactam first over colistin first.

6. RELATED SUMMARY MEASURES, PARADIGMS, AND METHODS

6.1 Related summary measures

The DOOR probability is related to other measures used in statistics. When restricted to a $2\times K$ table, Somers' d (Somers, 1962), defined by $d = \Pr[Y^{\mathrm{E}} > Y^{\mathrm{C}}] - \Pr[Y^{\mathrm{E}} < Y^{\mathrm{C}}]$, is equivalent to the DOOR probability as

$$d = \Pr[Y^{\mathrm{E}} > Y^{\mathrm{C}}] + \frac{1}{2}\Pr[Y^{\mathrm{E}} = Y^{\mathrm{C}}] - \left(\Pr[Y^{\mathrm{E}} < Y^{\mathrm{C}}] + \frac{1}{2}\Pr[Y^{\mathrm{E}} = Y^{\mathrm{C}}]\right) = \pi_{\mathrm{E}\geq\mathrm{C}} - (1 - \pi_{\mathrm{E}\geq\mathrm{C}})$$
$$= 2\pi_{\mathrm{E}\geq\mathrm{C}} - 1.$$

(Goodman and Kruskal, 1963). Somers' d is a measure of ordinal association between two possibly dependent random variables. It maps linearly the unit interval $[0,1]$ of $\pi_{\mathrm{E}\leq\mathrm{C}}$ onto $[-1,1]$, with

$$\phi\left(Y_i^{\mathrm{E}}, Y_j^{\mathrm{C}}\right) = \begin{cases} 1, & \text{if } Y_i^{\mathrm{E}} > Y_j^{\mathrm{C}}, \\ 0, & \text{if } Y_i^{\mathrm{E}} = Y_j^{\mathrm{C}} \\ -1, & \text{if } Y_i^{\mathrm{E}} < Y_j^{\mathrm{C}}. \end{cases}$$

Hochberg (1981) discussed estimation of the variance of d when comparing ordinal outcomes in two groups, constructing estimators of the variance of $\hat{d}$ by the delta method. Halperin et al. (1989) reported that the transformed version of their CI using the relationship $d = 2\pi_{\mathrm{E}\geq\mathrm{C}} - 1$ is superior to that by in Hochberg (1981). Buyse (2010) revisited this summary measure for pairwise comparisons of prioritized outcomes, termed the "net-treatment-benefit (NTB)", "proportion in favor of treatment" or "win difference". The NTB for each hierarchical is calculated $2\pi_{\mathrm{E}\geq\mathrm{C}} - 1$, which makes it closely related—but not exactly identical—to the DOOR probability, since the overall NTB is computed conditional on the previous ordered comparisons and combined accordingly. Furthermore, the DOOR probability is related to other summary measures commonly used in medical and clinical research. As described in Ryu and Agresti (2008), when $K = 2$, $\hat{\pi}_{\mathrm{E}\geq\mathrm{C}}$ is given by

$$\hat{\pi}_{\mathrm{E}\geq\mathrm{C}} = p_1^{\mathrm{E}} p_2^{\mathrm{C}} + \frac{1}{2}\left(p_1^{\mathrm{E}} p_1^{\mathrm{C}} + p_2^{\mathrm{E}} p_2^{\mathrm{C}}\right) = \frac{1}{2} + \frac{1}{2}\left(p_1^{\mathrm{E}} - p_1^{\mathrm{C}}\right),$$

and thus, it is equivalent to the difference in proportions $p_1^{\mathrm{E}} - p_1^{\mathrm{C}}$. The DOOR probability is equivalent to the area under the curve (AUC) for receiver operating characteristic (ROC) curves (Bamber, 1975) for binary classification or prediction of binary outcomes.

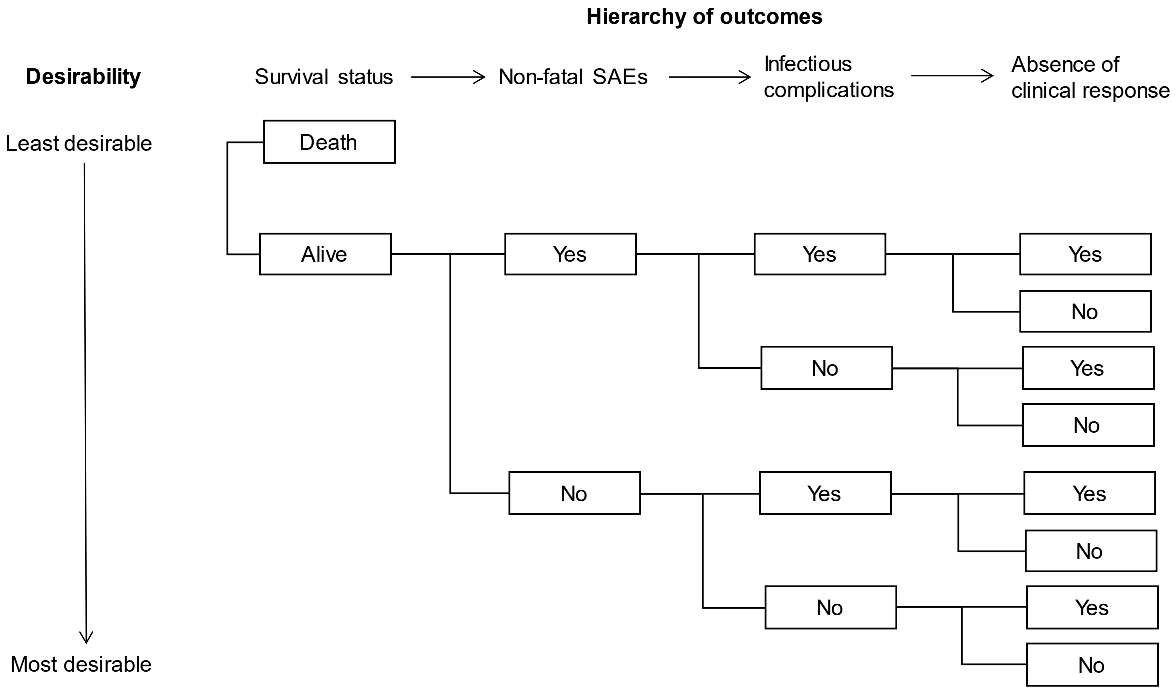

Figure 1. A DOOR outcome: survival status, and non-fatal SAEs, infectious complications, and absence of clinical response as tie-breaker variables, prioritizing safety over efficacy

6.2 Related paradigms and methods

The win ratio proposed by Pocock et al. (2012) is a paradigm that is related to the rank-based analyses in the DOOR paradigm. The DOOR and the win ratio both deal with multiple outcomes, but they differ in their composition strategy. The win ratio first establishes a prioritization of the multiple outcomes, called a "hierarchical composite outcome", where outcomes are ranked by clinical importance. For example, the hierarchy might be: survival status > non-fatal SAEs > infectious complications > absence of clinical response.

On the other hand, the DOOR offers more flexibility in the construction of the outcome, tailored to the disease of interest and the research question of a clinical trial. For example, the DOOR outcome by the ARLG developed for antibacterial-resistance treatments (Howard-Anderson et al., 2023) in Section 5, treats all components of the DOOR as equally important, with the exception of patient survival status, based on research by Doernberg at al. (2019) showing that clinician experts perceive the importance of such non-fatal events to be similar. Compositions in other settings may view the importance of events as dissimilar. Short-Course Outpatient Therapy of Community Acquired Pneumonia (SCOUT-CAP) was a multicenter randomized double-blind placebo-controlled superiority clinical trial evaluating a short-course (5 days) vs standard-course (10 days) strategy of oral β-lactam therapy for outpatient treatment of pediatric CAP (Williams et al., 2022). SCOUT-CAP developed the DOOR outcome with 8 ranked categories, including combination of three components and their status, where the components are adequate clinical response (absence of a medically attended visit, surgical procedure, or receipt of non-study antibiotics for persistent or worsening pneumonia after randomization,), resolution of symptoms, and the presence and severity (mild, moderate, and

Table 11. Two examples of the outcome distributions (%) for Treatments E and C

Survival >	Non-fatal SAEs	Case 1		Case 2	
		Treatment E	Treatment C	Treatment E	Treatment C
Dead		0.0	10.0	0.0	5.0
Alive	Yes	5.0	0.0	15.0	22.5
	No	95.0	90.0	85.0	72.5
$\widehat{\Pr[Y^{E} > Y^{C}]}$ (%)		10.0		24.1	
$\widehat{\Pr[Y^{E} < Y^{C}]}$ (%)		4.5		10.9	
$\widehat{\Pr[Y^{E} = Y^{C}]}$ (%)		85.5		65.0	
$\hat{\varphi}_{E\geq C}$		2.22		2.22	
$\hat{\pi}_{E\geq C}$ (%)		52.8		56.6	

severe) of antibiotic-associated adverse effects. The Dalbavancin as an Option for Treatment of S. aureus bacteremia (DOTS) clinical trial (Turner et al., 2022, 2025) adapted the DOOR methodology for design and analysis and developed the DOOR outcome, including the ARLG Bloodstream Infection QoL measure as one of the components. This measure was developed to assess QoL in patients with *S. aureus* and gram-negative bacterial bloodstream infections (King et al., 2021), where the QoL is used as a tie-breaker that adjusts for differences between patients in the same DOOR category level by stratifying patients into new levels based on a lower priority outcome.

The hierarchical composite outcome can be considered a special case of the DOOR framework when the DOOR outcome includes multiple tie-breaker variables (Evans et al., 2015; Turner et al., 2022, 2025), or when the outcome prioritizes either efficacy or safety (Howard-Anderson et al., 2023). A tie-breaker variable is used when comparing two patients who achieve the same primary DOOR outcome, allowing for a more nuanced distinction based on additional criteria. For instance, when prioritizing efficacy, the DOOR methodology places greater importance on the absence of clinical response than on non-fatal SAEs or infectious complications. Conversely, when prioritizing safety, the focus shifts toward avoiding non-fatal SAEs and infectious complications over ensuring clinical response. Figure 1 illustrates a DOOR outcome with 9-ranked categories, where patient survival status is considered first, and then if both patients are alive, tie-breakers such as fatal SAEs, infectious complications, and absence of clinical response are then applied, with safety prioritized over efficacy. Note that the evaluation of treatment for antimicrobial resistance is generally short-term and death is the worst outcome, so a tiebreaker is not used to compare the outcome if a patient dies. This avoids the problem of semi-competing risks; if the patient dies, the death event censors other clinical events.

The win ratio uses a "win ratio" as summary contrast statistic, which is the total number of patients having a more desirable outcome divided by the total numbers of patients having a less desirable outcome, i.e.,

$$\varphi_{E\geq C} = \frac{\Pr[Y^{E} > Y^{C}]}{\Pr[Y^{E} < Y^{C}]}.$$

When all components are time-to-event outcomes, under the proportional hazards assumption, the win ratio is equivalent to the inverse of the hazard ratio (Oakes, 2016). There is concern about the plausibility of the way it handles ties, similar to McNamar test because the win ratio only considers pairwise comparisons of two patients with a difference, and ignores pairwise comparisons of two patients with the same events, so it does not represent the chances of winning in the entire study population (Butler, Stockbridge and Packer, 2024).

Table 11 highlights a key issue with using the win ratio as a summary measure when the outcome includes both survival status and non-fatal SAEs, with survival status given higher priority. Although the outcome distributions for Treatments E and C differ significantly between Case 1 and Case 2, the win ratio remains consistent at approximately 2.22 in both cases. In contrast, the DOOR probability captures this difference, with an estimated probability of 52.2% for Case 1 and 56.6% for Case 2. This discrepancy highlights the potential limitations of the win ratio. As Butler et al. (2024) point out, the win ratio can overestimate the effects of interventions, as illustrated in the EMPULSE trial (Voors et al., 2022). As discussed in Section 1, absolute risks (e.g., the DOOR probability) summaries are more appropriate when synthesizing the result of multiple endpoints.

Dong et al. (2020) introduced the "win odds", a modified version of the win ratio that accounts for ties. The win odds is defined as

$$\psi_{\mathrm{E}\geq\mathrm{C}} = \frac{\pi_{\mathrm{E}\geq\mathrm{C}}}{\pi_{\mathrm{E}\leq\mathrm{C}}} = \frac{\pi_{\mathrm{E}\geq\mathrm{C}}}{1-\pi_{\mathrm{E}\geq\mathrm{C}}}.$$

The win odds is same as the WMWodds discussed in Agresti (1980). Calculations of the point estimate and the variance estimate for the Win Odds and the stratified Win Odds are discussed in Dong et al. (2020). As described in Section 2, the estimate of the win odds is biased. This bias is severe when the sample size is small, which means that a large sample size is required when using the win odds as a summary contrast measure. The relationship of the DOOR probability and other summary measures is summarized in Figure 2.

In practice, ordinal outcomes are often analyzed using the approaches below when comparing interventions.

- **Responder analysis**: An ordinal outcome is dichotomized into a binary outcome, i.e., responder ("alive with no events") and non-responder ("alive with at least one event" or "death"). A frequentist or Bayesian logistic regression is then used to estimate the odds ratio as a measure of the treatment effect, which is calculated by dividing the odds of the responders in the experimental group by the odds of the responders in the control group. The odds ratio gives an estimate of the relative risk if the responder is rare.
- **Regression analysis**: A ordinal outcome is analyzed using a frequentist or Bayesian proportional-odds regression method to estimate the odds ratio and associated CIs across all the categories (common odds ratio) as a summary contrast measure.

These approaches are suboptimal. The responder analysis is inefficient from the statistical perspective due to the loss of information from ignoring finer but important gradations of patient status. This may lead to decreased power or a necessary sample size increase to maintain power. Although the odds ratio and the common odds ratio are widely used measures of association for binary or ordinal outcomes, their interpretations are not intuitive nor particularly helpful for

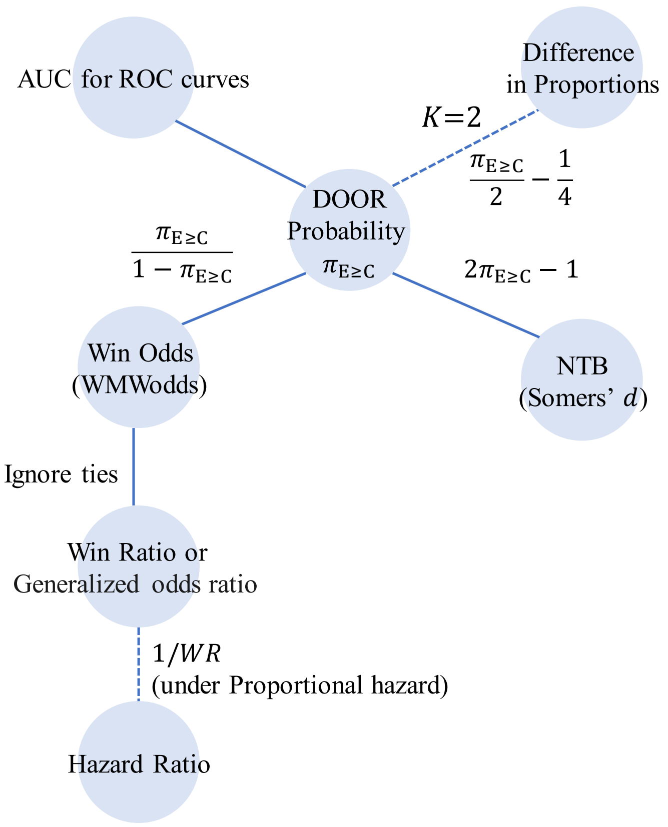

Figure 2. The relationship of the DOOR probability and other summary measures

clinical decision-making. Both measures can be obtained using frequentist or Bayesian regression models, which require assumptions, sometimes strong assumptions to hold in order for model-based inferences to be valid. For example, the proportional odds regression assumes "proportional odds", i.e., the intervention effects are consistent or proportional across categories of the ordinal outcome. If the model assumption does not hold, the estimates of the treatment effects may be biased. Meanwhile, Bayesian approaches concede: (i) robustness via greater reliance upon assumptions, (ii) objectivity via the incorporation of beliefs, (iii) transparency relenting to black box approaches, and (iv) the theoretical foundation for statistical inference. There is no ideal way to choose a prior and perspectives on priors can vary across clinicians, researchers, patients, regulators, and payors. There are challenges in interpreting the posterior distribution given that it incorporates a researchers' prior beliefs. These make results less accessible to researchers and the medical community. We recommend using the more robust approaches discussed in Sections 2 and 3 than modeling approaches that require assumptions for validity.

Table A9 summarizes qualifications for guiding principles criteria for various paradigms and analysis methods above.

1. **Paradigm or analysis method:** Both the DOOR and the win ratio are paradigm. Both aim to guide medical decision-making by providing a systematic way to evaluate effects of treatments or treatment strategies. Statistical methods, such as frequentist or Bayesian ordinal logistic regression, are specific techniques used to analyze ordinal outcomes and

to derive insights from it. These methods are often used within the DOOR paradigm to assess the DOOR outcomes.

2. **Outcome composition strategy:** Both the DOOR and the win ratio combine multiple outcomes into a single measure. The DOOR uses an ordinal composite outcome, which captures the trade-offs between component outcomes and reflect the cumulative nature of both benefits and harms for patients. In contrast, the win ratio utilizes a hierarchical composite outcome, where component outcomes are ranked by clinical importance. However, as described above, this hierarchical composite outcome can be seen as a special case of the DOOR framework when the composite outcome includes multiple tie-breaker variables or when there is a prioritization of either efficacy or safety. The DOOR offers more flexibility in constructing the outcome, allowing it to be tailored to the specific disease and research question of a clinical trial.

3. **Patient-centricity and pragmatism:** Both the DOOR and the win ratio analyze the patient's journey, acknowledging the cumulative impact of effects and identifying key gradations in patient response (3a and b). Especially the DOOR offers an APS plot which can visually tell a story of what events each patient experienced and how each patient's outcome contributes to the overall DOOR.

4. **Best practices:** The DOOR outcomes and hierarchical composite outcomes consist of multiple clinical outcomes, making it crucial to understand how each component and DOOR category contributes to the overall composite result. To facilitate this, the DOOR provides two forest plots. One forest plot summarizes the treatment effect on the DOOR and its components, using a consistent contrast measure, such as the DOOR probability with corresponding CIs (4a). The second forest plot illustrates the differences between the two DOOR outcome distributions, plotting the difference between the cumulative DOOR outcome distributions using DOOR probabilities (4c).

   In contrast, win ratio results may be presented through a generalized pairwise comparisons (GPC) flowchart (e.g., as shown in Verbeeck et al. (2023)), which highlights the cumulative effect of treatments (4c). While the DOOR provides an analysis based on the absolute scale, offering a common framework for interpreting multiple outcomes simultaneously, the win ratio uses relative risk, excluding ties, as previously discussed (4b). This creates challenges in interpreting the results. These same challenges can arise when conducting subgroup analyses.

5. **Statistical integrity:** Both the DOOR and win ratio methods employ distribution-free techniques to estimate their primary parameters of interest (i.e., the DOOR probability and win ratio), along with the associated confidence intervals (CIs) and hypothesis tests. The distribution-free techniques aim to minimize assumptions, providing robust statistical estimates (5a). For the DOOR, as discussed in Section 2, an unbiased estimate of the DOOR probability is derived from the estimated proportions of each category. The corresponding CI, discussed by Halperin et al. (1989), and the WMW test for the DOOR probability, are implemented in our online DOOR analysis tools. These methods control the coverage probability or Type I error probability at the nominal level adequately (5f). Although the hypothesis tests and CIs for the win ratio have been extensively discussed in the literature (e.g., Bebu and Lachin (2016)), it remains unclear whether they reliably

control errors as no extensive simulation evaluation, such as the one presented in this technical report, has been conducted.

Both can handle competing risks through the construction of composite outcomes (5b). In addition, both aim to perform ITT analyses. The DOOR calculates the DOOR probability based on all pairwise comparisons, whereas the win ratio considers only pairwise comparisons between two patients with a difference, ignoring ties (5d). This raises concerns about the generalizability of the results and over or underestimate the treatment effects (5c and e). Although the win odds accounts for ties, the estimate tends to be biased—particularly when the sample size is small. This bias can be quite substantial, indicating that a large sample size is necessary for the reliable use of the win odds as a summary measure.

As discussed in Section 3, the relative importance of each category can not be accounted for when calculating the DOOR probability. The partial credit analysis addresses this issue. The robustness of the results from the DOOR-probability-based analysis can be evaluated using the partial credit analysis (5h and 5i). In contrast, the win odds do not have a similar analysis tool.

Regression models for ordinal outcomes, such as proportional odds regression, can be used to analyze DOOR outcomes and summarize treatment effects through odds ratios (Jacobs et al., 2023). However, these models rely on assumptions, which may be strong and difficult to verify (5a and f). As noted earlier, applying Bayesian approaches introduces additional challenges, particularly due to their inherent subjectivity (5d). Using odds ratio create challenges in interpretation of the result (5e): it can be difficult to interpret, especially when they are far from 1. For example, an odds ratio of 0.1 or 10 does not have an easily understood direct relationship with the magnitude of treatment effect in terms of absolute risk. If the event of interest is rare, the odds ratio can become misleading. In such cases, the odds ratio tends to exaggerate the treatment effect, especially when events are infrequent in both the treatment and control groups. The odds ratio may not accurately reflect the true risk difference between groups when the outcome is rare. The odds ratio assumes a consistent relationship between exposure and outcome, but the effect size may vary across different levels of treatment or population subgroups. This non-linearity is not captured well by a single common odds ratio, leading to potential misrepresentation of the treatment effect.

7. SUMMARY AND FURTHER DEVELOPMENTS

Thorough understanding of the benefits and harms of interventions is essential for clinical decision-making. Traditional approaches to benefit:risk assessment do not directly provide the evidence needed to make medical decisions as they often rely on combining separate marginal analyses of each endpoint. This approach fails to incorporate associations between or the cumulative nature of multiple outcomes in individual patients, suffers from competing risk complexities during interpretation of individual outcomes, fails to recognize important gradations of patient responses, and since efficacy and safety analyses are often conducted on different populations, benefit:risk generalizability is unclear. The DOOR, a paradigm for the design, monitoring, analysis, and interpretation of clinical trials and other research studies based on patient-centered risk-benefit assessment, is designed to address these issues and provide

useful information for medical decision-making (Evans et al., 2015; Evans and Follmann, 2016). To evaluate the benefits and risks of interventions, the DOOR adopts an ordinal outcome with gradations of patient response to account for important differences in the ultimate response to a therapeutic intervention. Interventions are compared by estimating the DOOR probability, i.e., the probability of a participant assigned to one intervention having a more desirable outcome than a participant assigned to the other intervention.

In this report, we described statistical issues in the implementation of the DOOR methodology, in particular two statistical methods for the analysis of clinical trials and other clinical research using the DOOR methodology, the rank-based approach and the grade-based analysis. We provided the recommended statistical analysis plan for DOOR methodology and illustrated the statistical analyses for DOOR outcomes with three clinical studies.

The determination of sample size and the evaluation of power are fundamental and critical elements in the design of a clinical trial. If a sample size is too small, important effects may not be detected; if it is too large, it is a waste of resources and unethically puts many participants at unnecessary risk. As the DOOR probability is estimated using the WMW statistic for ordinal outcomes, it is natural to implement a sample-size calculation using the WMW test-based method (e.g., see Noether (1987), Zhao, Rahardja and Qu (2008), Tang (2011)). Some software packages (e.g., East® (Cytel), nQuery (Statistical Solutions), SAS® (SAS Institute)) have functions to implement the sample-size calculation. See Hamasaki et al. (2024) for more details.

Ongoing work on DOOR methodology includes: (1) subgroup analyses as subgroups should be identified and evaluated based on patient-centric benefit:risk rather than naïve evaluation of a single variable; (2) longitudinal evaluation of DOOR as a dynamic patient state, recognizing the importance of knowing not only whether events occurred, but when, for how long, and whether they resolved or relapsed (Follmann et al., 2020; Shu et al., 2024; Shu et al., 2025); (3) integrated benefit:risk analysis of multiple trials using DOOR outcomes using stratified-analysis or meta-analysis approach, and (4) group-sequential and adaptive designs for monitoring trials using DOOR outcomes.

ACKNOWLEDGEMENTS

This work was supported by the National Institute of Allergy and Infectious Diseases (NIAID) of the National Institutes of Health (NIH) (award number UM1AI104681). The content is solely the responsibility of the authors and does not necessarily represent the official views of the NIH.

REFERENCES

Bamber D. The area above the ordinal dominance graph and the area below the receiver operating characteristic graph. Journal of Mathematical Psychology 1975; 12:387-415.

Bartlett MS. The use of transformations. Biometrics 1947; 3:39–52.

Bebu I, Lachin JM. Large sample inference for a win ratio analysis of a composite outcome based on prioritized components. Biostatistics. 2016; 17:178-187.

Beigel JH, Tomashek KM, Dodd LE, Mehta AK, Zingman BS, Kalil AC, Hohmann E, Chu HY, Luetkemeyer A, Kline S, Lopez de Castilla D, Finberg RW, Dierberg K, Tapson V, Hsieh L,

Patterson TF, Paredes R, Sweeney DA, Short WR, Touloumi G, Lye DC, Ohmagari N, Oh MD, Ruiz-Palacios GM, Benfield T, Fätkenheuer G, Kortepeter MG, Atmar RL, Creech CB, Lundgren J, Babiker AG, Pett S, Neaton JD, Burgess TH, Bonnett T, Green M, Makowski M, Osinusi A, Nayak S, Lane HC; ACTT-1 Study Group Members. Remdesivir for the Treatment of Covid-19 - Final Report. New England Journal of Medicine 2020; 383:1813-1826.

Brunner E, Munzel, U. The nonparametric Behrens-Fisher problem: Asymptotic theory and a small-sample approximation. Biometrical Journal. 2000; 42:17–25

Butler J, Stockbridge N, Packer M. Win ratio: A seductive but potentially misleading method for evaluating evidence from clinical trials. Circulation. 2024; 149:1546-1548

Buyse M. Generalized pairwise comparisons of prioritized outcomes in the two-sample problem. Statistics in Medicine 2010; 29:3245- 3257.

Chamberlain JM, Kapur J, Silbergleit RS, Elm JJ, Rosenthal ES, Bleck TP, Shinnar S, Zetabchi S, Evans SR. Desirability of outcome ranking for status epilepticus: A benefit-risk approach to design and analyses of clinical SE trials. Neurology 2023; 101:e1633-e1639.

Chao A, Cheng K F. Interval estimation of reliability for highly reliable stress-strength models. Chinese Journal of Mathematics 1985; 13:131-136.

DeMets DL, Califf RM. A historical perspective on clinical trials innovation and leadership: where have the academics gone? JAMA 2011; 305:713-714.

Dong G, Hoaglin DC, Qiu J, Matsouaka RA, Chang YW, Wang J, Vandemeulebroecke M. The win ratio: On interpretation and handling of ties. Statistics in Biopharmaceutical Research 2020; 12:99-106.

Doernberg SB, Tran TTT, Tong SYC, Paul M, Yahav D, Davis JS, Leibovici L, Boucher HW, Corey GR, Cosgrove SE, Chambers HF, Fowler VG, Evans SR, Holland TL; Antibacterial Resistance Leadership Group. Good studies evaluate the disease while great studies evaluate the patient: Development and application of a desirability of outcome ranking endpoint for staphylococcus aureus bloodstream infection. Clinical Infectious Disease 2019; 68:1691-1698.

Edwardes MD. A confidence interval for $\Pr(X < Y) - \Pr(X > Y)$ estimated from simple cluster samples. Biometrics 1995; 51:571-578.

Evans SR. Our most important discovery: The question. Statistics in Biopharmaceutical Research 2022; 14:398-407.

Evans SR, Follmann D. Using outcomes to analyze patients rather than patients to analyze outcomes: A step toward pragmatism in benefit:risk evaluation. Statistics in Biopharmaceutical Research 2016; 8:386-393.

Evans SR, Knutsson M, Amarenco P, Albers GW, Bath PM, Denison H, Ladenvall P, Jonasson J, Easton JD, Minematsu K, Molina CA, Wang Y, Wong KL, Johnston SC. Methodologies for pragmatic and efficient assessment of benefits and harms: Application to the SOCRATES trial. Clin Trials 2020; 17:617-626.

Evans SR, Rubin D, Follmann D, Pennello G, Huskins WC, Powers JH, Schoenfeld D, Chuang-Stein C, Cosgrove SE, Fowler VG Jr, Lautenbach E, Chambers HF. Desirability of Outcome

Ranking (DOOR) and Response Adjusted for Duration of Antibiotic Risk (RADAR). Clinical Infectious Disease 2015; 61:800-806.

Follmann D, Fay MP, Hamasaki T, Evans S. Analysis of ordered composite endpoints. Statistics in Medicine 2020; 39:602-616.

Gewandter JS, Brell J, Cavaletti G, Dougherty PM, Evans S, Howie L, McDermott MP, O'Mara A, Smith AG, Dastros-Pitei D, Gauthier LR, Haroutounian S, Jarpe M, Katz NP, Loprinzi C, Richardson P, Lavoie-Smith EM, Wen PY, Turk DC, Dworkin RH, Freeman R. Trial designs for chemotherapy-induced peripheral neuropathy prevention: ACTTION recommendations. Neurology 2018; 91:403-413.

Goodman LA, Kruskal WH. Measures of association for cross classifications III: Approximate sampling theory. Journal of the American Statistical Association 1963; 58:310–364.

Halperin M, Hamdy MI, Thall PF. Distribution-free confidence intervals for a parameter of Wilcoxon-Mann-Whitney type for ordered categories and progressive censoring. Biometrics 1989; 45:509–521.

Hamasaki T, Evans SR. The Disability of Outcome ranking (DOOR). In: Buyse M, Verbeeck J, De Backer M, Deltuvaite-Thomas V, Saad ED, Molenberghs G, eds. Handbook of Generalized Pairwise Comparisons: Methods for Patient-Centric Analysis, Boca Raton, Chapman & Hall/CRC; 2025:280-295.

Hamasaki T, He Y, Wu Q, Evans SR. Design of clinical trials with the desirability of outcome ranking methodology. In Chen DG, ed. Biostatistics in Biopharmaceutical Research and Development, Vol. 1, Cham: Springer; 2024, 137-159.

Hochberg Y. On the variance estimate of a Wilcoxon-Mann Whitney statistic for group ordered data. Communication in Statistics- Theory and Methods 1981; A10:1719-1732.

Howard-Anderson J, Hamasaki T, Dai W, Collyar D, Rubin D, Nambiar S, Kinamon T, Hill C, Gelone SP, Mariano D, Baba T, Holland TL, Doernberg SB, Chambers HF, Fowler VG, Evans SR, Boucher HW. Improving traditional registrational trial end points: Development and application of a desirability ofoutcome ranking end point for complicated urinary tract infection clinical trials. Clinical Infectious Diseases 2023; 76:e1157-e1165.

Jacobs MA, Schmidt S, Hall DE, Stitzenberg KB, Kao LS, Wang CP, Manuel LS, Shireman PK. Differentiating urgent from elective cases matters in minority populations: Developing an ordinal “Desirability of Outcome Ranking” to increase granularity and sensitivity of surgical outcomes assessment. Journal of the American College of Surgery 2023; 237:545-555.

Johnston SC, Amarenco P, Denison H, Evans SR, Himmelmann A, James S, Knutsson M, Ladenvall P, Molina CA, Wang Y; THALES Investigators. Ticagrelor and Aspirin or Aspirin Alone in Acute Ischemic Stroke or TIA. The New England Journal of Medicine 2020; 383:207-217.

Kinamon T, Gopinath R, Waack U, Needles M, Rubin D, Collyar D, Doernberg SB, Evans S, Hamasaki T, Holland TL, Howard-Anderson J, Chambers H, Fowler VG, Nambiar S, Kim P, Boucher HW. Exploration of a potential desirability of outcome ranking endpoint for complicated intra-abdominal infections using 9 registrational trials for antibacterial drugs. Clinical Infectious Diseases 2023; 77:649-656.

Kleykamp BA, Dworkin RH, Turk DC, Bhagwagar Z, Cowan P, Eccleston C, Ellenberg SS, Evans SR, Farrar JT, Freeman RL, Garrison LP, Gewandter JS, Goli V, Iyengar S, Jadad AR, Jensen MP, Junor R, Katz NP, Kesslak JP, Kopecky EA, Lissin D, Markman JD, McDermott MP, Mease PJ, O'Connor AB, Patel KV, Raja SN, Rowbotham MC, Sampaio C, Singh JA, Steigerwald I, Strand V, Tive LA, Tobias J, Wasan AD, Wilson HD. Benefit-risk assessment and reporting in clinical trials of chronic pain treatments: IMMPACT recommendations. Pain 2022;163:1006-1018.

Klotz JH. The Wilcoxon, ties, and the computer. Journal of the American Statistical Association 1966; 61:772–787.

Lang JB. Multinomial-Poisson homogeneous models for contingency tables. The Annals of Statistics 2004; 32:340–383.

Lang JB. Homogeneous linear predictor models for contingency tables. Journal of the American Statistical Association 2005; 100: 121-134.

Lehmann EL. Non-Parametric: Statistical Methods Based on Ranks. New York: Springer, 1975.

Lodise TP, Min J, Nathanson BH, Yücel E. Comparison of early treatment with ceftolozane/tazobactam versus polymyxin-based therapy of pneumonia due to MDR Pseudomonasaeruginosa (PUMA). Antimicrob Agents Chemotherapy 2025; e0056925. doi: 10.1128/aac.00569-25.

Lu Y, Zhao Q, Zou J, Yan S, Tamaresis JS, Nelson L, Tu XM, Chen J, Tian L. A composite endpoint for treatment benefit according to patient preference. Statistics in Biopharmaceutical Research 2022;14:408-422.

Mann HB, Whitney DR. On a test of whether one of two random variables is stochastically larger than the other. The Annals of Mathematical Statistics 1947; 18:50–60.

Naber KG, Llorens L, Kaniga K, Kotey P, Hedrich D, Redman R. Intravenous doripenem at 500 milligrams versus levofloxacin at 250 milligrams, with an option to switch to oral therapy, for treatment of complicated lower urinary tract infection and pyelonephritis. Antimicrobial Agents and Chemotherapy 2009; 53:3782-3792.

Newcombe RG. Confidence intervals for an effect size measure based on the Mann–Whitney statistic. Part 2: asymptotic methods and evaluation. Statistics in Medicine 2006; 25:559–573.

Noether GE. Sample-size determination for some common nonparametric tests. Journal of the American Statistical Association 1987; 82:645–647.

Oakes D. On the win-ratio statistic in clinical trials with multiple types of event. Biometrika 2016; 103:742–745.

O'Brien RG, Castelloe JM. Exploiting the link between the Wilcoxon-Mann-Whitney test and a simple odds statistic. Proceedings of the 31st Annual SAS Users Group International Conference, SAS Institute Inc: Cary, NC, 2006; 209–231.

Pocock SJ, Ariti CA, Collier TJ, Wang D. The win ratio: a new approach to the analysis of composite endpoints in clinical trials based on clinical priorities. European Heart Journal 33; 2012:176–182.

Tang Y. Size and power estimation for the Wilcoxon-Mann-Whitney test for ordered categorical data. Statistics in Medicine 2011; 30:3461-3470.

Ryu E. Modeling and Inference for an Ordinal Effect Size Measure. Ph.D. Dissertation, University of Florida, 2007.

Ryu E, Agresti A. Modeling and Inference for an Ordinal Effect Size Measure. Statistics in Medicine. 2008; 27:1703-1717.

Wilcoxon F. Individual comparisons by ranking methods. Biometrics Bulletin 1945; 1:80–83.

Sandoval GJ, Grobman WA, Evans SR, Rice MM, Clifton RG, Chauhan SP, Costantine MM, Gibson KS, Longo M, Metz TD, Miller ES, Parry S, Reddy UM, Rouse DJ, Simhan HN, Thorp JM Jr, Tita ATN, Saade GR; Eunice Kennedy Shriver National Institute of Child Health and Human Development Maternal-Fetal Medicine Units (MFMU) Network. Desirability of outcome ranking for obstetrical trials: Illustration and application to the ARRIVE trial. American Journal of Obstetrics and Gynecology. 2024; 230:370.e1-370.e12.

Simonoff JS, Hochberg Y, Reiser B. A confidence interval for $\Pr(X < Y) - \Pr(X > Y)$ estimated from simple cluster samples. Biometrics 1986; 42:895-907. Erratum in: Biometrics 1989; 45:701.

Simonoff JS, Hochberg Y, Reiser B. Response to C. Brownie's reader reaction. Biometrics 1988; 44:621.

Somers RH. A new asymmetric measure of association for ordinal variables American Sociological Review 1962; 27:799-811.

Shu S, Diao G, Hamasaki T, Scott SR. Longitudinal benefit:risk analysis through the desirability of outcome ranking (DOOR) with Application to ACTT-1 Trial. Statistics in Biopharmaceutical Research 2025; 17:488–495.

Shu S, Diao G, Hamasaki T, Scott SR. Desirability of outcome ranking (DOOR) analysis for multivariate survival outcomes with application to ACTT-1 trial. Clinical Trials 2025 (Accepted on 12 August 2025)

Turner NA, Hamasaki T, Doernberg SB, Lodise TP, King HA, Ghazaryan V, Cosgrove SE, Jenkins TC, Liu C, Sharma S, Zaharoff S, Wahid L, Renard VJ, Cook P, Raad I, Hachem R, Chaftari AM, Sims M, DeMarco C, Miller LG, McCarthy MW, Morse CG, Lucasti C, Forrest GN, Cherabuddi K, Polk C, Fazili T, Rupp ME, Thompson GR 3rd, Kim K, Strnad L, Schnee AE, McKinnell JA, Ramesh M, Silveira FP, McCarty TP, Lee TC, McDonald EG, Paolino K, Wiegand K, Wall A, Riccobene T, Patel R, Rappo U, Evans S, Chambers HF, Fowler VG Jr, Holland TL; Antibacterial Resistance Leadership Group. Dalbavancin for treatment of *Staphylococcus aureus* bacteremia: The DOTS randomized clinical trial. JAMA. 2025; 334:866-877.

Turner NA, Zaharoff S, King H, Evans S, Hamasaki T, Lodise T, Ghazaryan V, Beresnev T, Riccobene T, Patel R, Doernberg SB, Rappo U, Fowler VG Jr, Holland TL; Antibacterial Resistance Leadership Group (ARLG). Dalbavancin as an option for treatment of *S. aureus* bacteremia (DOTS): study protocol for a phase 2b, multicenter, randomized, open-label clinical trial. Trials 2022; 23:407.

van Duin D, Lok JJ, Earley M, Cober E, Richter SS, Perez F, Salata RA, Kalayjian RC, Watkins RR, Doi Y, Kaye KS, Fowler VG Jr, Paterson DL, Bonomo RA, Evans S, Antibacterial Resistance Leadership Group. Colistin versus Ceftazidime-Avibactam in the treatment of infections Due to carbapenem-Resistant enterobacteriaceae. Clinical Infection Disease 2018; 66:163-171.

Verbeeck J, De Backer M, Verwerft J, Salvaggio S, Valgimigli M, Vranckx P, Buyse M, Brunner E. Generalized pairwise comparisons to assess treatment tffects: JACC review topic of the week. Journal of the American College of Cardiology 2023; 82:1360-1372.

Voors AA, Angermann CE, Teerlink JR, Collins SP, Kosiborod M, Biegus J, Ferreira JP, Nassif ME, Psotka MA, Tromp J, Borleffs CJW, Ma C, Comin-Colet J, Fu M, Janssens SP, Kiss RG, Mentz RJ, Sakata Y, Schirmer H, Schou M, Schulze PC, Spinarova L, Volterrani M, Wranicz JK, Zeymer U, Zieroth S, Brueckmann M, Blatchford JP, Salsali A, Ponikowski P. The SGLT2 inhibitor empagliflozin in patients hospitalized for acute heart failure: a multinational randomized trial. Nature Medicine 2022; 28:568-574.

Wilcoxon F. Individual comparisons by ranking methods. Biometrics Bulletin 1945; 1:80.

Williams DJ, Creech CB, Walter EB, Martin JM, Gerber JS, Newland JG, Howard L, Hofto ME, Staat MA, Oler RE, Tuyishimire B, Conrad TM, Lee MS, Ghazaryan V, Pettigrew MM, Fowler VG Jr, Chambers HF, Zaoutis TE, Evans S, Huskins WC; The DMID 14-0079 Study Team. Short- vs Standard-Course Outpatient Antibiotic Therapy for Community-Acquired Pneumonia in Children: The SCOUT-CAP Randomized Clinical Trial. JAMA Pediatric. 2022; 176:253-261.

Zhao YD, Rahardja D, Qu Y. Sample size calculation for the Wilcoxon-Mann-Whitney test adjusting for ties. Statistics in Medicine 2008; 27:462–468.

APPENDIX: TABLES AND FIGURES

Tables

Table A1. DOOR category proportions (%) and true value of $\pi_{E \geq C}$ (%). Simulation settings for evaluating the actual coverage probabilities of the confidence intervals. A smaller K represents a more desirable outcome.

		Experimental $P_1^E, \ldots, P_K^E$				Control $P_1^C, \ldots, P_K^C$				True value
#	K	1	2	3		1	2	3		$\pi_{E \geq C}$
S1	3	60	30	10		60	30	10		50.0
S2		20	60	20		20	60	20		50.0
S3		10	30	60		10	30	60		50.0
S4		40	20	40		40	20	40		50.0
S5		60	30	10		50	40	10		54.5
S6		60	30	10		40	50	10		59.0
S7		60	30	10		30	60	10		63.5
S8		20	60	20		20	50	30		54.0
S9		20	60	20		20	40	40		58.0
S10		20	60	20		20	30	50		62.0
#	K	1	2	3	4	1	2	3	4	$\pi_{E \geq C}$
S11	4	40	30	20	10	40	30	20	10	50.0
S12		10	20	30	40	10	20	30	40	50.0
S13		10	40	40	10	10	40	40	10	50.0

S14		40	10	10	40	40	10	10	40	50.0
S15		40	30	20	10	30	40	20	10	53.5
S16		40	30	20	10	20	50	20	10	57.0
S17		40	30	20	10	20	40	30	10	59.5
S18		20	50	20	10	10	60	20	10	53.5
S19		20	50	20	10	10	50	30	10	57.0
S20		20	50	20	10	10	40	40	10	60.5

#	K	1	2	3	4	5	1	2	3	4	5	$\pi_{E \geq C}$
S21	5	60	20	10	5	5	60	20	10	5	5	50.0
S22		5	5	10	20	60	5	5	10	20	60	50.0
S23		5	20	50	20	5	5	20	50	20	5	50.0
S24		30	15	10	15	30	30	15	10	15	30	50.0
S25		60	20	10	5	5	50	30	10	5	5	54.0
S26		60	20	10	5	5	40	40	10	5	5	58.0
S27		60	20	10	5	5	30	50	10	5	5	62.0
S28		20	50	20	5	5	20	40	30	5	5	53.5
S29		20	50	20	5	5	20	30	40	5	5	57.0
S30		20	50	20	5	5	20	20	50	5	5	60.5

#	K	1	2	3	4	5	6	7	8	9	1	2	3	4	5	6	7	8	9	$\pi_{E \geq C}$
S31	9	30	20	15	10	10	5	5	3	2	30	20	15	10	10	5	5	3	2	50.0
S32		2	3	5	5	10	10	15	20	30	2	3	5	5	10	10	15	20	30	50.0
S33		2	3	10	20	30	20	10	3	2	2	3	10	20	30	20	10	3	2	50.0
S34		20	15	10	4	2	4	10	15	20	20	15	10	4	2	4	10	15	20	50.0
S35		30	20	15	10	10	5	5	3	2	20	30	15	10	10	5	5	3	2	52.5
S36		30	20	15	10	10	5	5	3	2	10	40	15	10	10	5	5	3	2	55.0
S37		30	20	15	10	10	5	5	3	2	10	30	25	10	10	5	5	3	2	56.8
S38		20	30	15	10	10	5	5	3	2	10	40	15	10	10	5	5	3	2	52.5
S39		20	30	15	10	10	5	5	3	2	10	30	25	10	10	5	5	3	2	54.8
S40		20	30	15	10	10	5	5	3	2	5	35	25	10	10	5	5	3	2	56.0

Table A2. Features of the DOOR app: Standard and professional editions

	Standard edition	Professional edition
**Data Input**	A summary table of DOOR outcome distributions and DOOR components by 2 intervention groups	Individual patient-level data
**Analysis Functions**		
**1. Descriptive Analysis**		
Summary table of DOOR outcome distributions by 2 intervention groups	✓	✓
Bar chart of DOOR outcome distributions by 2 intervention groups	✓	✓
Summary table of DOOR components by 2 intervention groups	✓	✓

Bar chart of DOOR components by 2 intervention groups	✓	✓
Anthology of patient stories plot		✓
**2. Rank-based Analysis**		
DOOR probability forest plot including DOOR and components and their confidence intervals (the method by Halperin et al. (1989) and its improved method using pseudo-score approach), and p-value from normal approximation WMW test without continuity correction	✓	✓
DOOR probability cumulative forest plot	✓	✓
**3. Grade-based Analysis**		
Partial credits summary	✓	✓
Bivariate plot of difference in partial credit means vs. DOOR probability	✓	✓
**4. Tie-breaker Analysis**		✓
**5. Inverse Propensity Weighting**		✓
**Other Functions**		
Label customization	✓	✓
Save data	✓	✓

Table A3. DORI-05: The distribution of DOOR outcome and respective components by intervention group

	Doripenem (DOR)				Levofloxacin (LEV)				DOR - LEV	
			Cum.				Cum.		Expected gain or loss	
**DOOR category/components**	**N**	**(%)**	**N**	**(%)**	**N**	**(%)**	**N**	**(%)**	**Gain or loss**	**Cum.**
Alive with no events	263	70.3	263	70.3	253	67.6	253	67.6	27	27
Alive with 1 event	93	24.9	356	95.2	111	29.7	364	97.3	-48	-21
Alive with 2 events	16	4.3	372	99.5	9	2.4	373	99.7	19	-3
Alive with 3 events	1	0.3	373	99.7	1	0.3	374	100.0	0	-3
Death	1	0.3	374	100.0	0	0.0	374	100.0	3	0
Absence of clinical success	81	21.7			113	30.2			-86	
Infectious complications	23	6.1			5	1.3			48	
Non-fatal SAEs	25	6.7			14	3.7			29	
Death	1	0.3			0	0.0			3	

Table A4. DORI-05: Partial credit analyses summary

**DOOR category**[$]	**Grading Key**							
	**A**		**B**		**C**		**D**	
DOOR 1	100		100		100		100	
DOOR 2	100		100		100		0	
DOOR 3	100		100		0		0	
DOOR 4	100		0		0		0	
DOOR 5	0		0		0		0	
**DORI-05**	**DOR**	**LEV**	**DOR**	**LEV**	**DOR**	**LEV**	**DOR**	**LEV**
Mean (SD)	99.7 (5.2)	100.0 (0.0)	99.5 (7.3)	99.7 (5.2)	95.2 (21.4)	97.3 (16.2)	70.3 (45.7)	67.6 (46.8)
Diff. in means (95% CI)	-0.3 (-0.8, 0.3)		-0.2 (-1.2, 0.6)		-2.1 (-4.9, 0.6)		2.7 (-4.0, 9.3)	
P-value*	0.3180		0.5635		0.1237		0.4299	
DOOR prob. (%) (95% CI)	49.9 (49.5, 50.2)		49.9 (49.3, 50.4)		48.9 (47.5, 50.3)		51.3 (48.0, 54.6)	
P-value**	0.3173		0.5632		0.1236		0.4296	

DOR: Doripenem; LEV: Levofloxacin

[$] DOOR 1: Alive with no events; DOOR 2: Alive with 1 event; DOOR 3: Alive with 2 events; DOOR 4: Alive with 3 events; DOOR 5: Death

* P-value from Welch t-test for difference in partial credit means between the groups; ** P-value from WMW test (normal approximation without continuity correction) for the DOOR probability

Table A5. ACTT-1: The distribution of DOOR outcome and respective components by intervention group

	Remdesivir (RDV)				Placebo (PLB)				RDV - PLB	
			Cum.				Cum.		Expected gain or loss	
**DOOR category/components**	**N**	**(%)**	**N**	**(%)**	**N**	**(%)**	**N**	**(%)**	**Gain or loss**	**Cum.**
Alive with no events	433	80.0	433	80.0	382	73.3	382	73.3	67	67
Alive with 1 event	42	7.8	475	87.8	57	10.9	439	84.3	-32	35
Alive with 2 events	8	1.5	483	89.3	6	1.2	445	85.4	3	39
Death	58	10.7	541	100.0	76	14.6	521	100.0	-39	0
Hosp. with IMV/ECMO	45	8.3			55	10.6			-22	
Non-fatal SAEs	11	2.0			13	2.5			-5	
Death	58	10.7			76	14.6			-39	

Table A6. ACTT-1: Partial credit analyses summary

DOOR category[$]	Grading Key							
	A		B		C		D	
DOOR 1	100		100		100		100	
DOOR 2	100		0		100		80	
DOOR 3	100		0		0		60	
DOOR 4	0		0		0		0	
**ACTT-1**	**RDV**	**PLB**	**RDV**	**PLB**	**RDV**	**PLB**	**RDV**	**PLB**
Mean (SD)	89.3 (31.0)	85.4 (35.3)	80.0 (40.0)	73.3 (44.3)	87.8 (32.8)	84.3 (36.5)	87.1 (31.0)	82.8 (35.0)
Diff. in means (95% CI)	3.9 (-0.1, 7.9)		6.7 (1.6, 11.8)		3.5 (-0.6, 7.7)		4.3 (0.4, 8.4)	
P-value*	0.0585		0.0097		0.0968		0.0318	
DOOR prob. (%) (95% CI)	51.9 (49.9, 53.9)		53.4 (50.8, 55.9)		51.8 (49.7, 53.9)		53.3 (50.8, 55.9)	
P-value**	0.0579		0.0096		0.0961		0.0105	

RDV: Remdesivir; PLB: Placebo

$ DOOR 1: Alive with no events; DOOR 2: Alive with 1 event; DOOR 3: Alive with 2 events; DOOR 4: Death

* P-value from Welch t-test for difference in partial credit means between the groups; ** P-value from WMM test (normal approximation without continuity correction) for the DOOR probability

Table A7. CRACKLE: The distribution of DOOR outcome and respective components by intervention group

	Ceftazidime-avibactam (CAZ-AVI)				Colistin (CST)				CAZ-AVI - CST	
**DOOR category/components**	**N**	**(%)**	**Cum. N**	**(%)**	**N**	**(%)**	**Cum. N**	**(%)**	**Expected gain or loss Gain or loss**	**Cum.**
**a. Naïve (Unadjusted)**										
Discharged home	6	23.1	6	23.1	4	8.7	4	8.7	144	144
Alive in hospital or discharged not to home, no renal failure incident	17	65.4	23	88.5	25	54.3	29	63.0	110	254
Alive in hospital or discharged not to home, renal failure incident	1	3.8	24	92.3	5	10.9	34	73.9	-70	184
Hospital death	2	7.7	26	100.0	12	26.1	46	100.0	-184	0
Renal failure	1	3.8			7	15.2			-114	
Not discharged	20	76.9			42	91.3			-144	
Death	2	7.7			12	26.1			-184	
**b. IPW-adjusted**										
Discharged home		20.5		20.5		7.8		7.8	126	126
Alive in hospital or discharged not to home, no renal failure incident		65.4		85.9		55.9		63.7	95	221
Alive in hospital or discharged not to home, renal failure incident		5.1		90.9		11.3		75.0	-62	159
Hospital death		9.1		100.0		25.0		100.0	-159	0
Renal failure		5.1				14.9			-98	
Not discharged		79.5				92.2			-126	
Death		9.1				25.0			-159	

Table A8. CRACKLE: Partial credit analyses summary

DOOR category[$]	Grading Key							
	A		B		C		D	
DOOR 1	100		100		100		100	
DOOR 2	100		0		100		80	
DOOR 3	100		0		0		60	
DOOR 4	0		0		0		0	
**a. CRACKLE (naïve)**	**CAZ-AVI**	**CST**	**CAZ-AVI**	**CST**	**CAZ-AVI**	**CST**	**CAZ-AVI**	**CST**
Mean (SD)	92.3 (27.2)	73.9 (44.4)	23.1 (43.0)	8.7 (28.5)	88.5 (32.6)	63.0 (48.8)	77.7 (24.9)	58.7 (36.4)
Diff. in means (95% CI)	18.4 (1.6, 35.2)		14.4 (-4.7, 33.4)		25.5 (6.2, 44.6)		19.0 (4.5, 33.5)	
P-value[*]	0.0327		0.1350		0.0102		0.0108	
DOOR prob. (%) (95% CI)	59.2 (50.5, 67.4)		57.2 (47.6, 66.3)		62.7 (52.7, 71.7)		66.2 (54.4, 76.3)	
P-value[**]	0.0600		0.0924		0.0216		0.0106	
**b. CRACKLE (adjusted)**	**CAZ-AVI**	**CST**	**CAZ-AVI**	**CST**	**CAZ-AVI**	**CST**	**CAZ-AVI**	**CST**
Mean (SD)	90.9 (29.3)	75.0 (43.8)	20.5 (41.1)	7.8 (27.2)	85.9 (35.5)	63.7 (48.6)	75.8 (26.3)	59.3 (35.7)
Diff. in means (95% CI)	15.9 (-1.3, 33.2)		12.6 (-5.6, 30.9)		22.1 (2.2, 42.1)		16.5 (1.8, 31.2)	
P-value[*]	0.0695		0.1696		0.0303		0.0286	
DOOR prob. (%) (95% CI)	58.0 (49.1, 66.4)		56.3 (47.1, 65.1)		61.1 (50.8, 70.4)		64.3 (52.4, 74.7)	
P-value[**]	0.1019		0.1213		0.0466		0.0229	

CAZ-AVI: Ceftazidime-avibactam; CST: Colistin

$ DOOR 1: Discharged home; DOOR 2: Alive in hospital or discharged not to home, no renal failure incident; DOOR 3: Alive in hospital or discharged not to home, renal failure incident; DOOR 4: Hospital death

* P-value from Welch t-test for difference in partial credit means between the groups; ** P-value from WMM test (normal approximation without continuity correction) for the DOOR probability

Table A9. Comparison of qualifications for guiding principles criteria for various paradigms and analysis methodologies

Guiding principle for maximizing pragmatism, robustness, replicability, objectivity, and transparency	DOOR	Win Ratio	Frequentist or Bayesian Ordinal Logistic Regression
**1. Paradigm or analysis method**	Paradigm	Paradigm	Analysis method
**2. Outcome composition strategy**	Ordinal outcome	Hierarchical outcome	N/A
**3. Patient-centricity and pragmatism**			
3a. Analyze the patient story/journey; recognize the cumulative nature of effects	✓	✓	N/A
3b. Distinguish important gradations of patient response	✓	✓	N/A
**4. Best practices**			
4a. Composite endpoints (integrated presentation of component analyses)	✓		N/A
4b. Multi-outcomes/benefit:risk analyses (analyses based on the absolute risk scale providing a common scale for simultaneous interpretation of multiple outcomes)	✓		N/A
4c. Ordinal outcomes (cumulative analyses)	✓	Generalized pairwise comparisons flowchart (e.g., see Verbeeck et al. (2023))	N/A
**5. Statistical integrity**			
5a. Robustness: avoid/minimize reliance upon assumptions e.g., common odds, distribution of treatment effects, specification of model form, for analysis validity	✓	✓	
5b. Incorporate competing risks	✓	✓	N/A
5c. Intention-to-treat principle with full analysis set for all outcomes (clarity of generalizability; known applicability at the time of treatment initiation)	✓	?	N/A

Guiding principle for maximizing pragmatism, robustness, replicability, objectivity, and transparency	DOOR	Win Ratio	Frequentist or Bayesian Ordinal Logistic Regression
5d. Objectivity: free from subjective beliefs	✓	✓	✓<br>Bayesian methods: noninformative prior only
5e. Defined population parameters and estimands	✓	?	
5f. Theoretical foundation for the confirmatory evidence standard<br>• Unbiased estimates of treatment effects<br>• Correct coverage probability for confidence interval estimation<br>• Error control in hypothesis testing	✓	✓<br>Conditional upon: large sample size	✓<br>Frequentist methods: conditional upon model correctness<br><br>Bayesian methods: conditional upon (i) model correctness, and (ii) noninformative prior
5g. Incorporation of ties into rank-based statistics utilizing pair-wise comparisons	✓		NA
5h. Implementation of rank-based and grade-based analyses of treatment contrast	✓		
5i. Evaluation of robustness of grade-based analyses	✓	N/A	N/A

N/A: Not Applicable; ?: Questionable

Figures

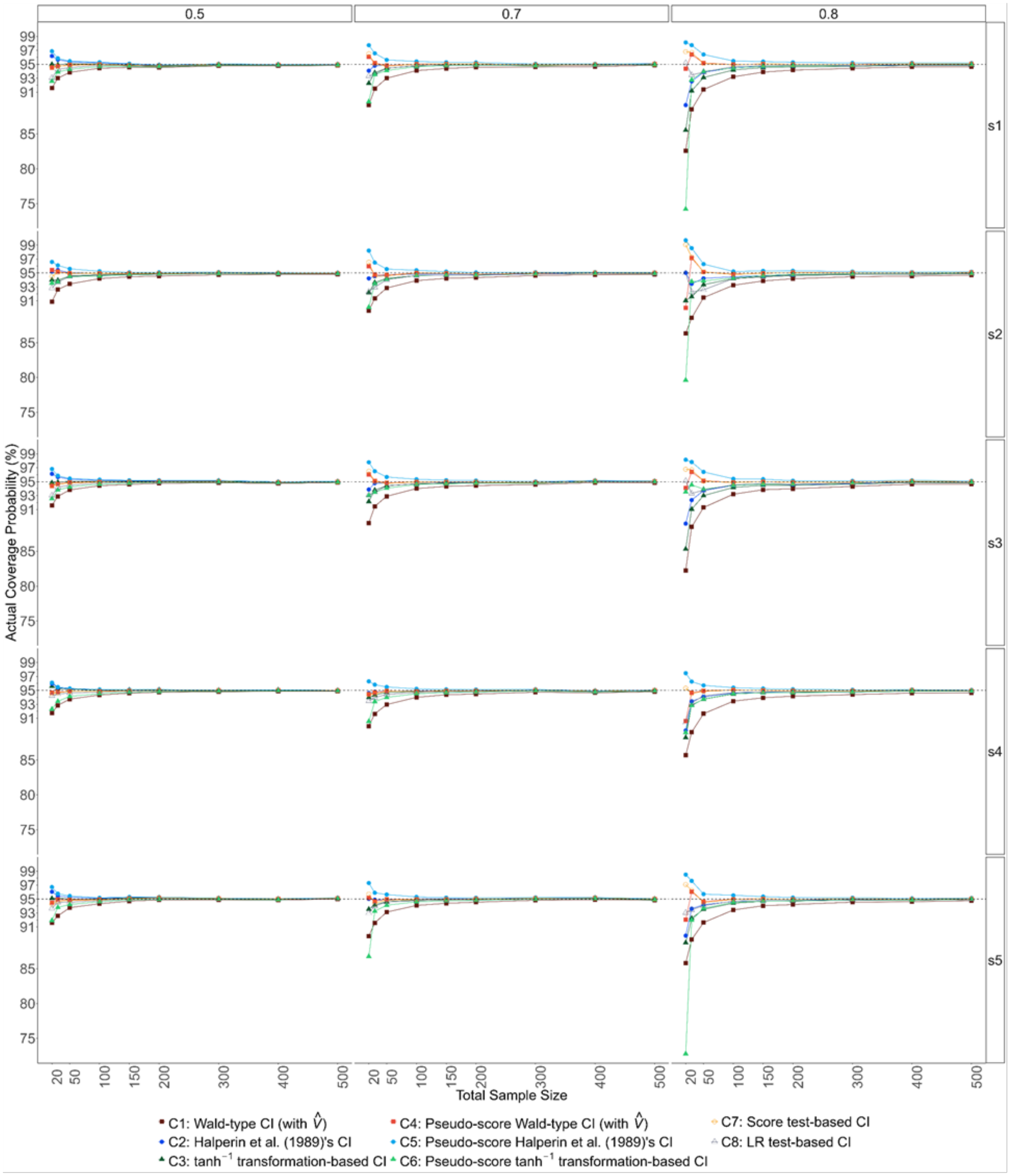

Figure A1. Behavior of the actual coverage probability of the two-sided 95% CI estimate with varying sample sizes, sample size allocation, and the shape of the DOOR outcome distribution ($K = 3$; S1-S5)

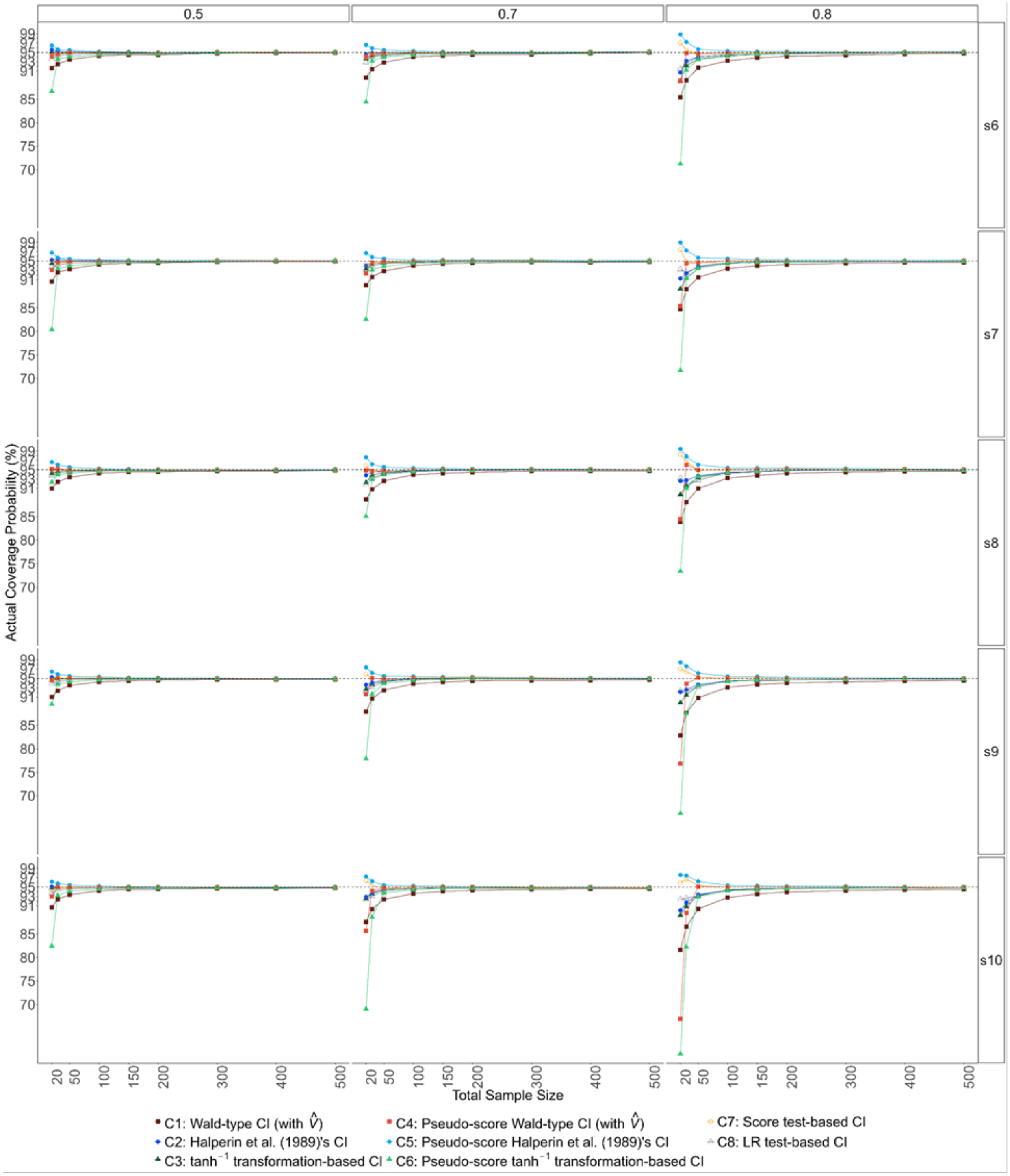

Figure A2. Behavior of the actual coverage probability of the two-sided 95% CI estimate with varying sample sizes, sample size allocation, and the shape of the DOOR outcome distribution ($K = 3$; S6-S10)

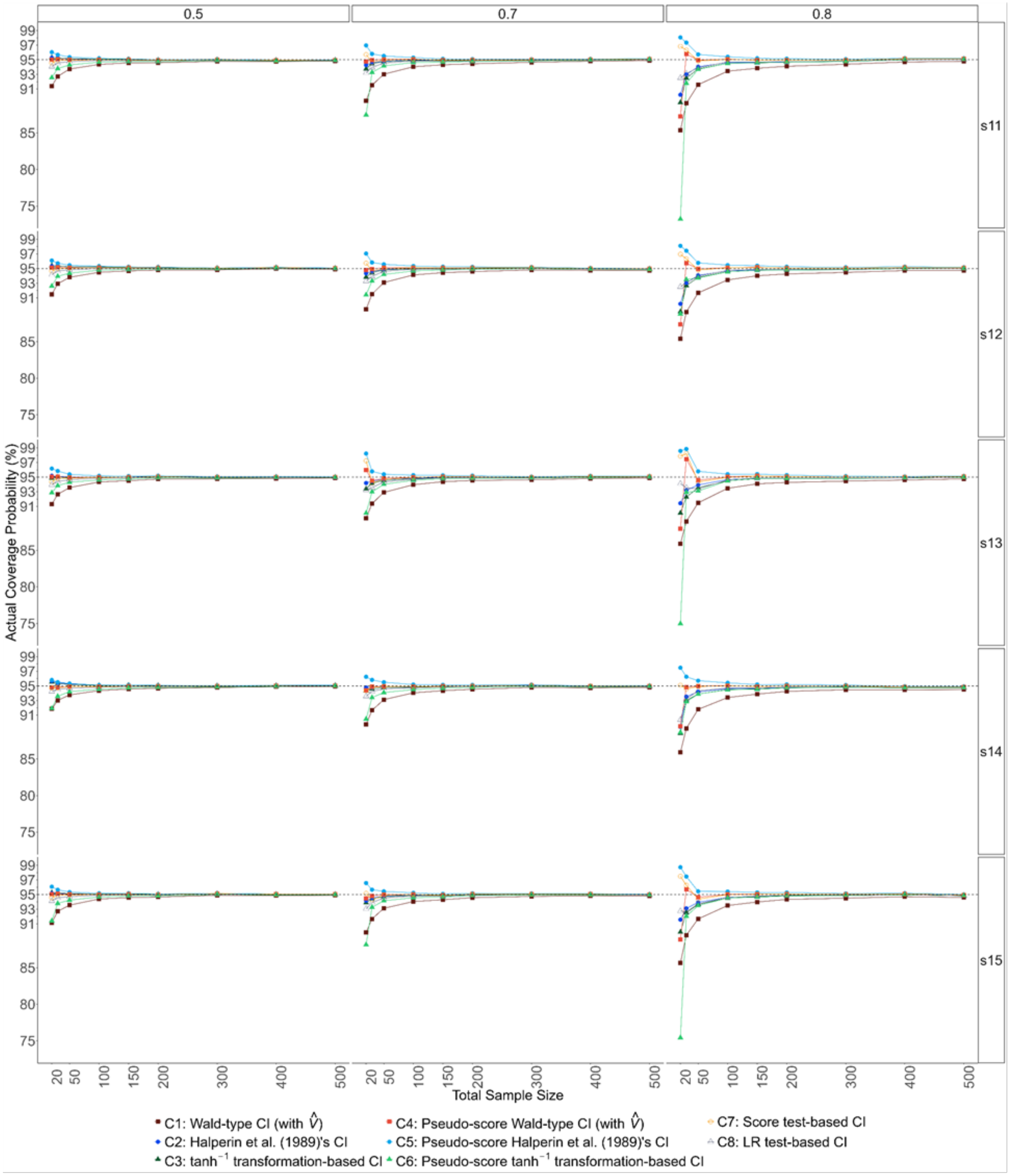

Figure A3. Behavior of the actual coverage probability of the two-sided 95% CI estimate with varying sample sizes, sample size allocation, and the shape of the DOOR outcome distribution ($K = 4$; S11 – S15)

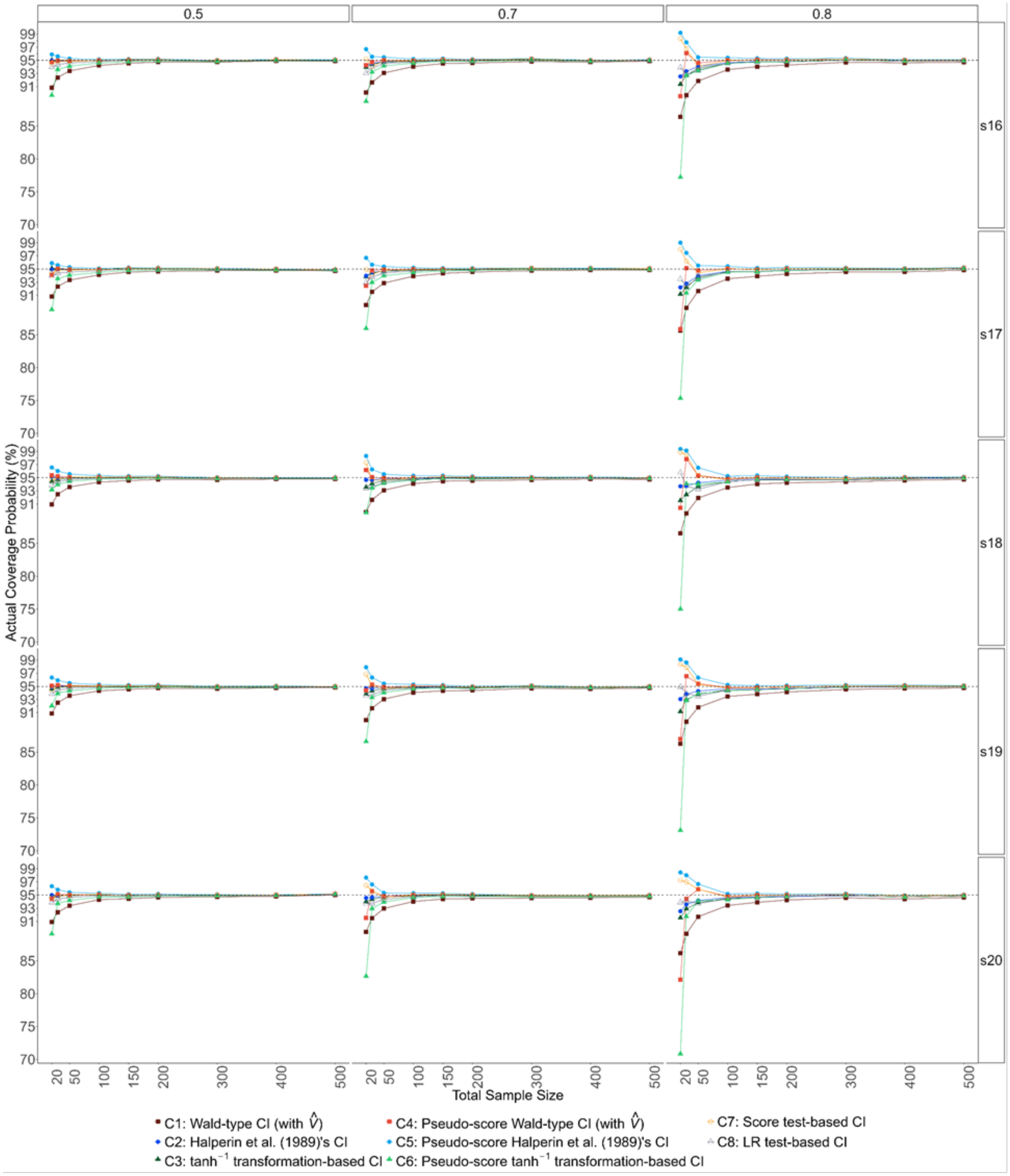

Figure A4. Behavior of the actual coverage probability of the two-sided 95% CI estimate with varying sample sizes, sample size allocation, and the shape of the DOOR outcome distribution ($K = 4$; S16 – S20)

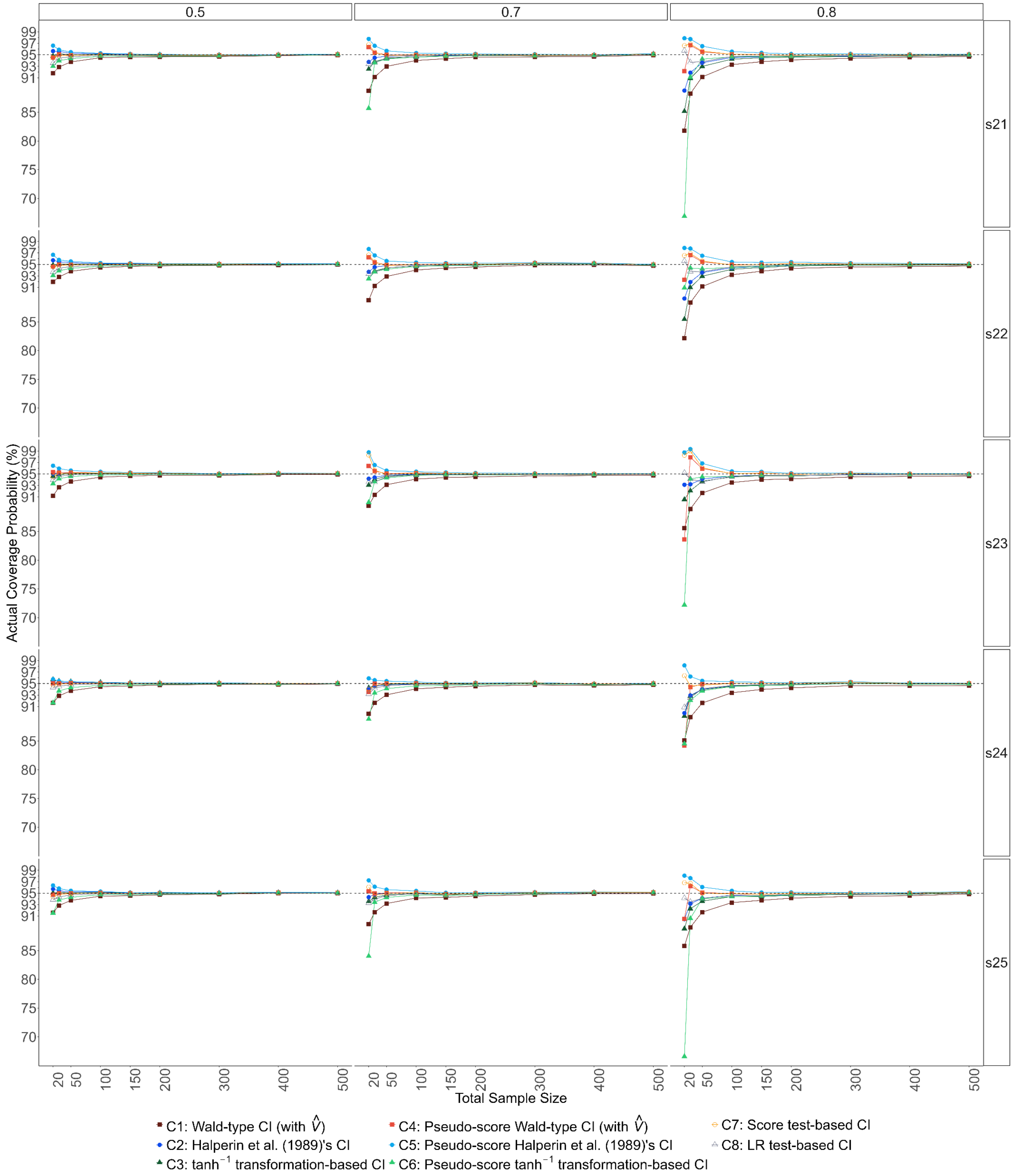

Figure A5. Behavior of the actual coverage probability of the two-sided 95% CI estimate with varying sample sizes, sample size allocation, and the shape of the DOOR outcome distribution ($K = 5$; S21 – S25)

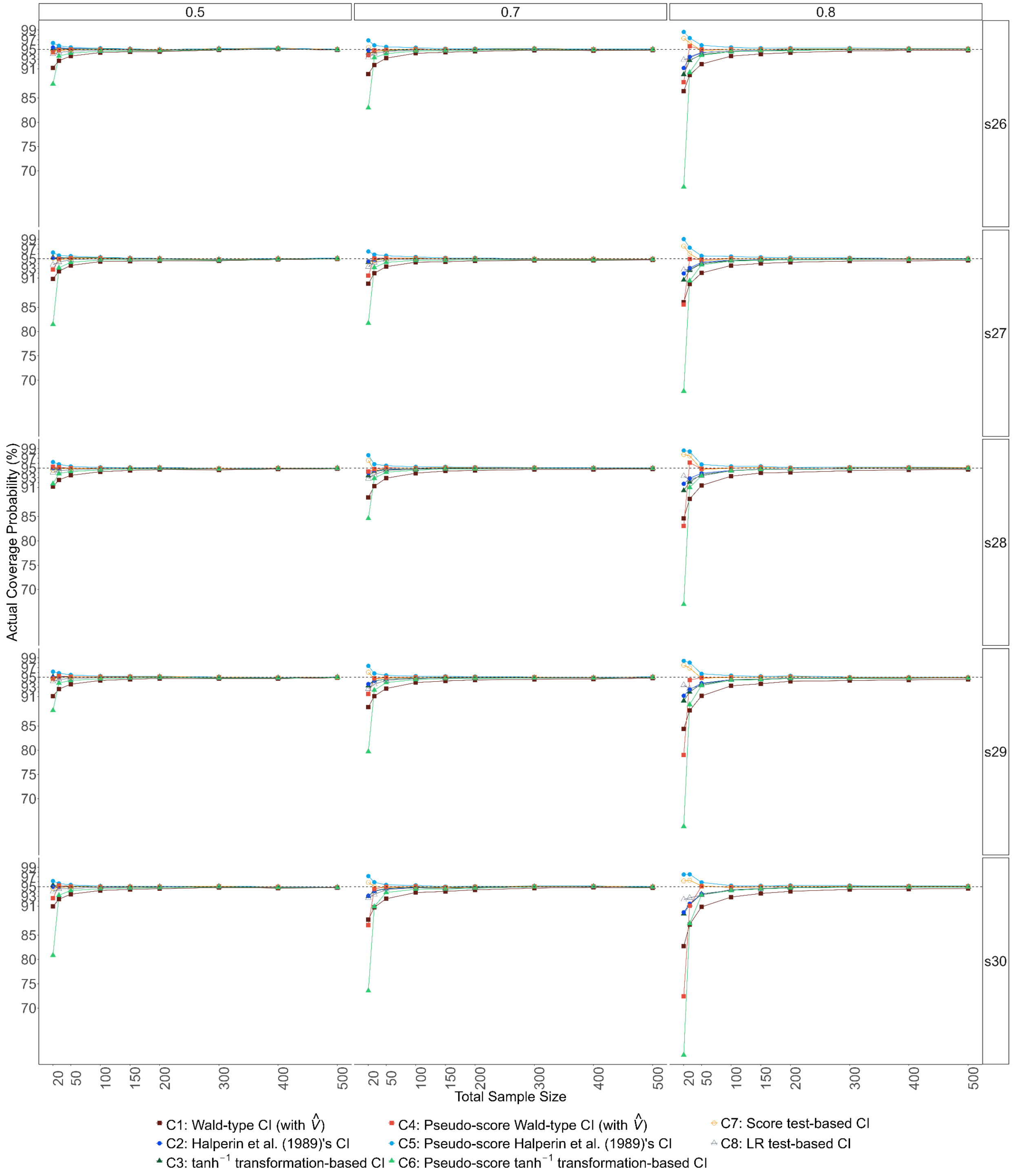

Figure A6. Behavior of the actual coverage probability of the two-sided 95% CI estimate with varying sample sizes, sample size allocation, and the shape of the DOOR outcome distribution ($K = 5$; S26 – S30)

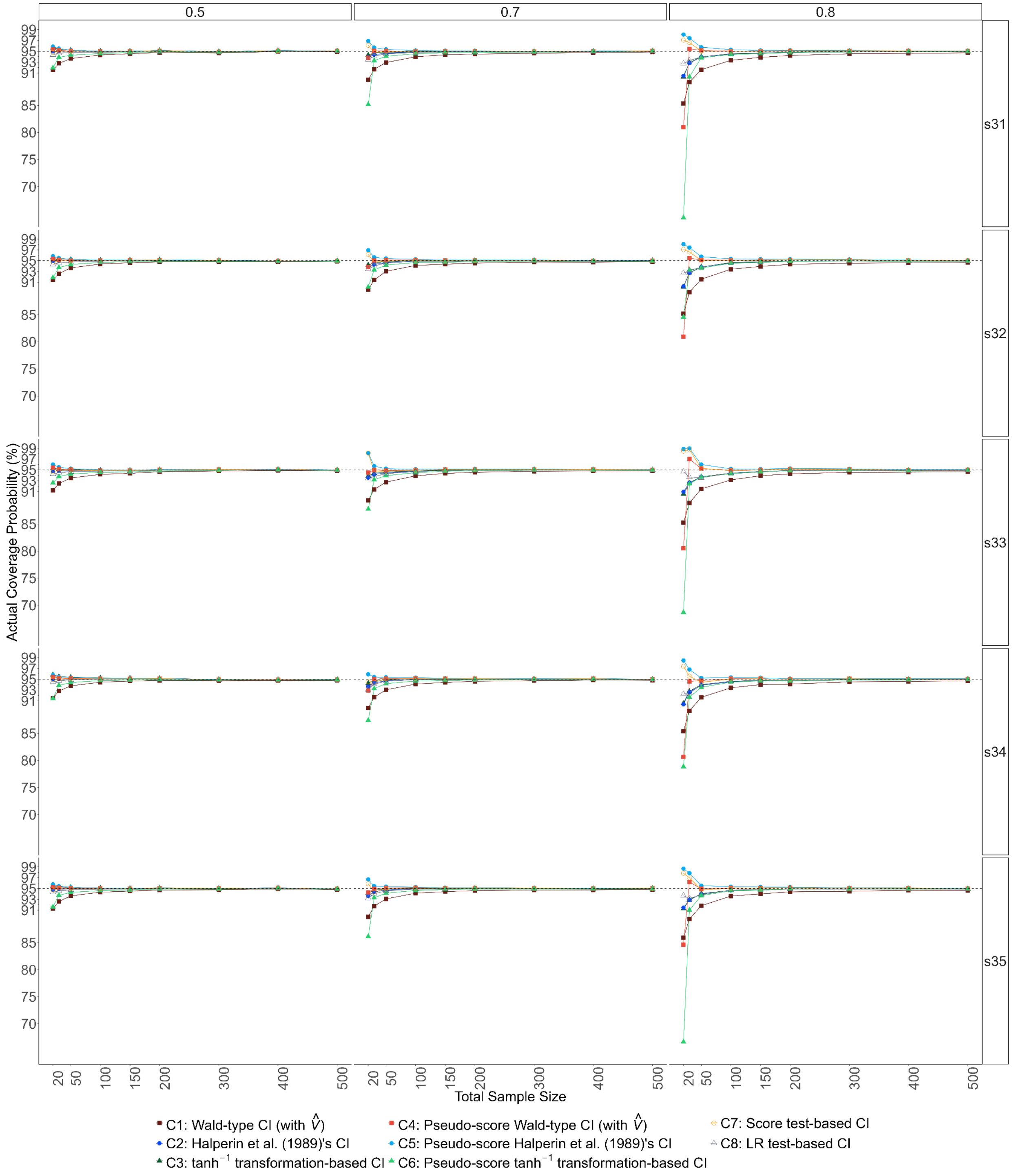

Figure A7. Behavior of the actual coverage probability of the two-sided 95% CI estimate with varying sample sizes, sample size allocation, and the shape of the DOOR outcome distribution ($K = 9$; S31 – S35)

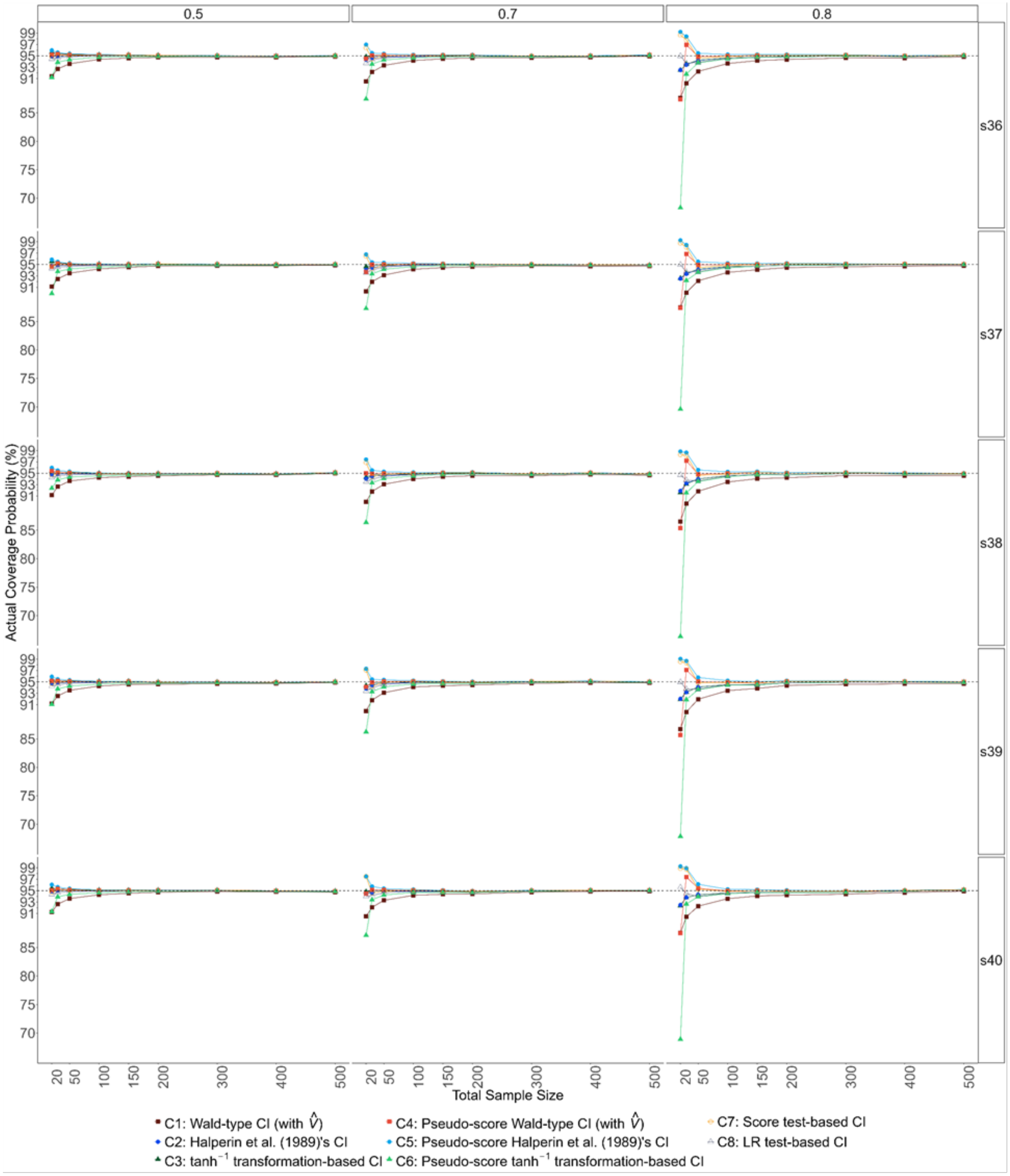

Figure A8. Behavior of the actual coverage probability of the two-sided 95% CI estimate with varying sample sizes, sample size allocation, and the shape of the DOOR outcome distribution ($K = 9$; S36 – S40)

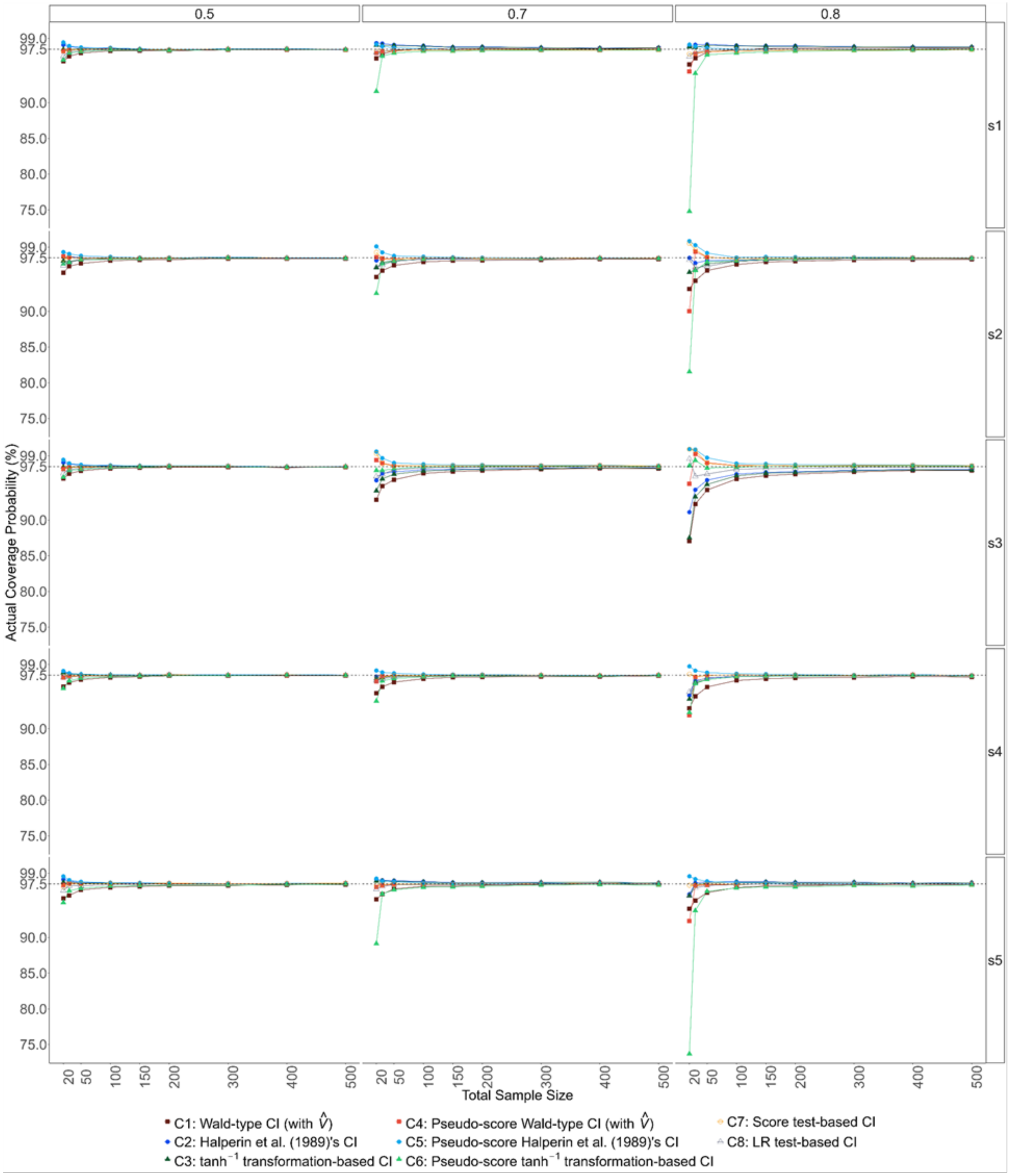

Figure A9. Behavior of the actual coverage probability of the one-sided 97.5% lower CI estimate with varying sample sizes, sample size allocation, and the shape of the DOOR outcome distribution ($K = 3$; S1–S5)

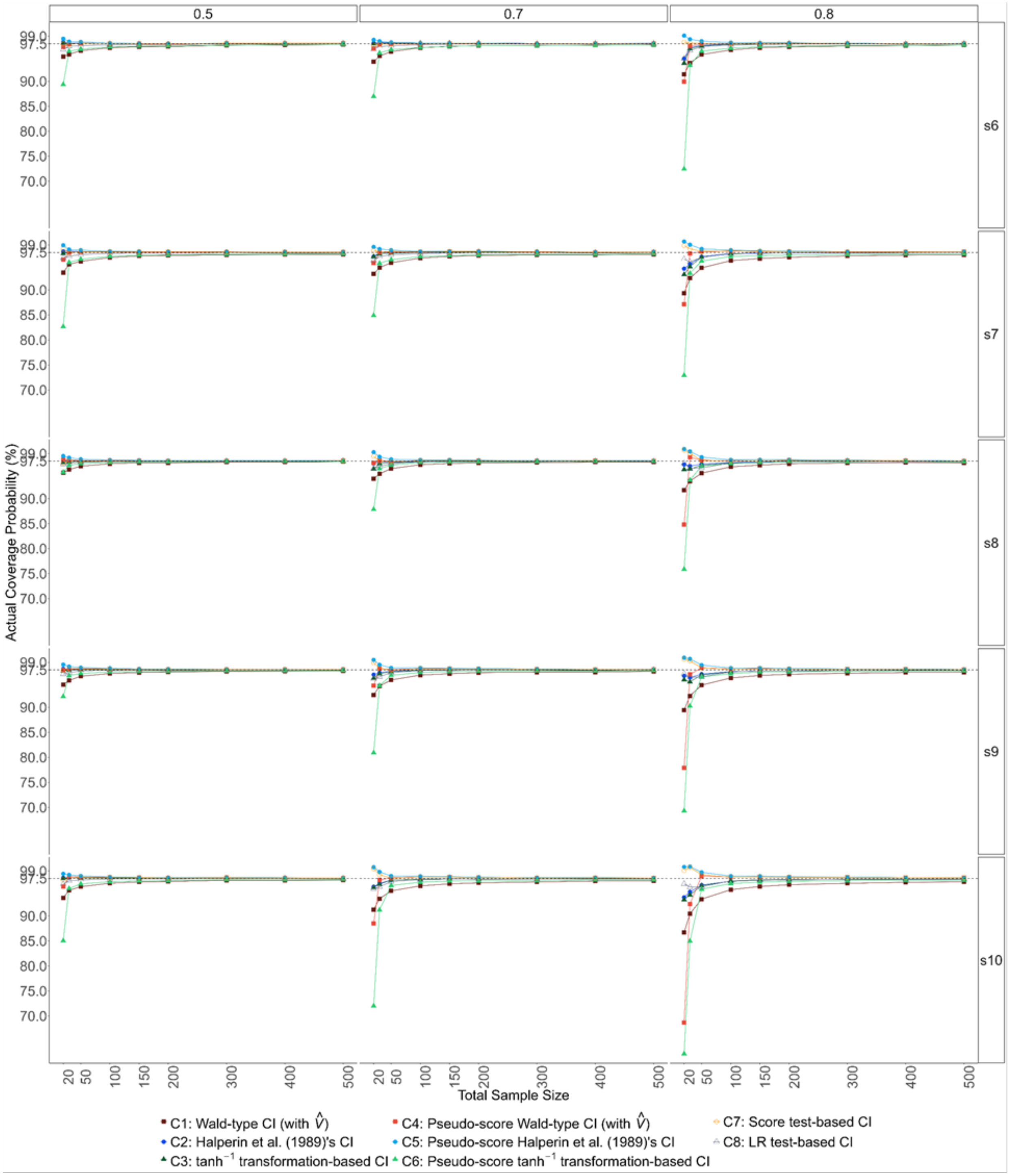

Figure A10. Behavior of the actual coverage probability of the one-sided 97.5% lower CI estimate with varying sample sizes, sample size allocation, and the shape of the DOOR outcome distribution ($K = 3$; S6–S10)

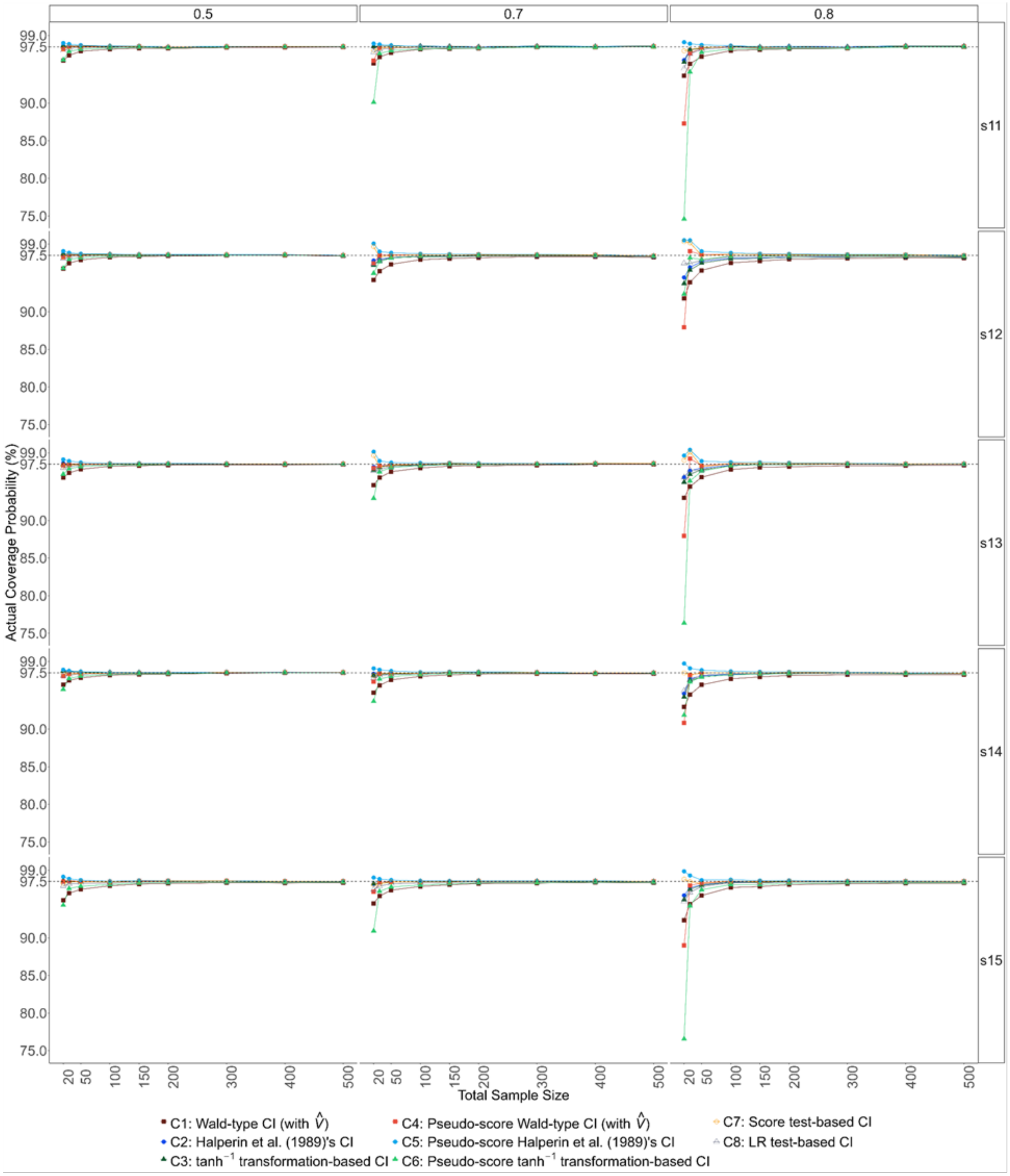

Figure A11. Behavior of the actual coverage probability of the one-sided 97.5% lower CI estimate with varying sample sizes, sample size allocation, and the shape of the DOOR outcome distribution ($K = 4$; S11–S15)

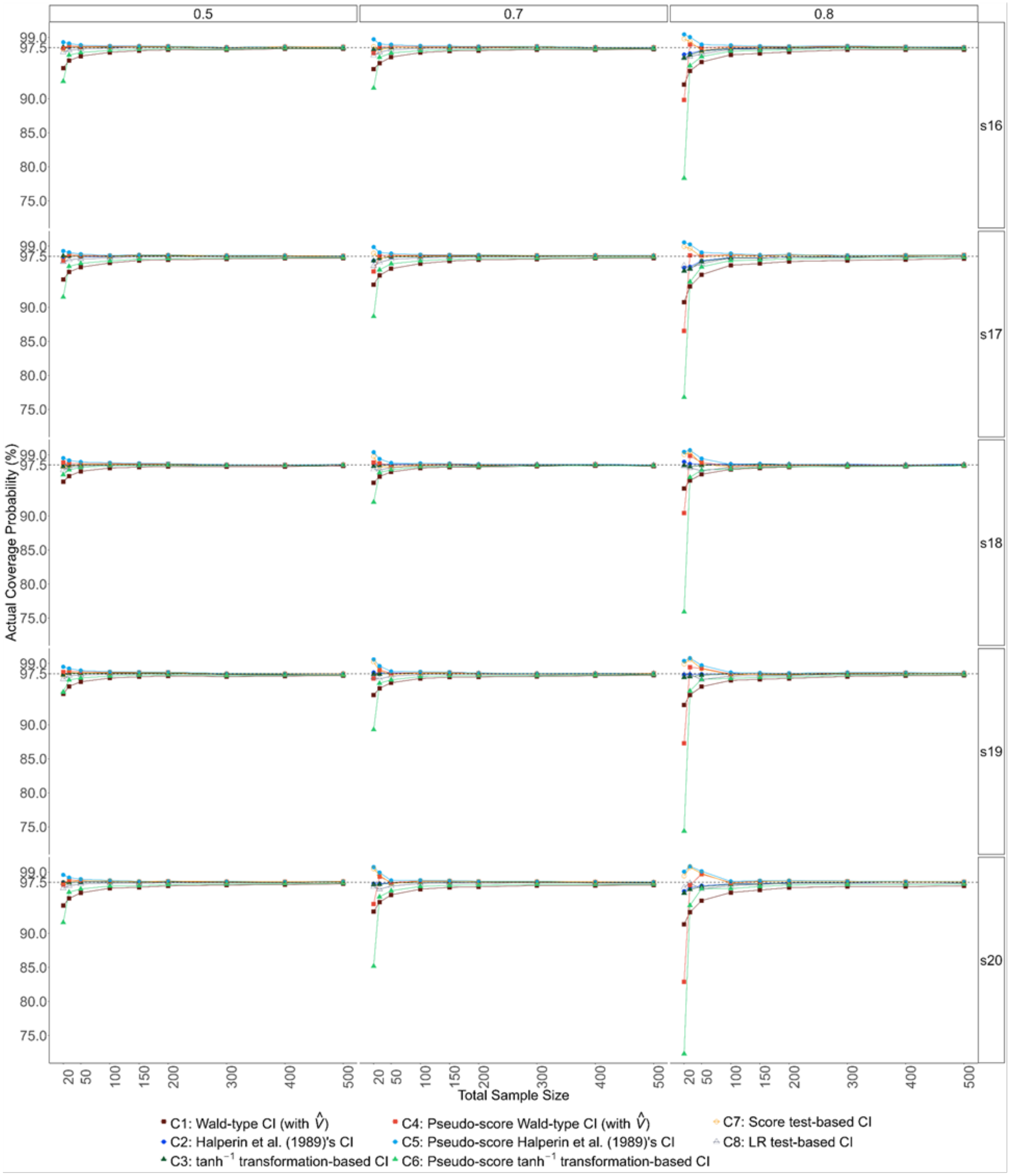

Figure A12. Behavior of the actual coverage probability of the one-sided 97.5% lower CI estimate with varying sample sizes, sample size allocation, and the shape of the DOOR outcome distribution ($K = 4$; S16–S20)

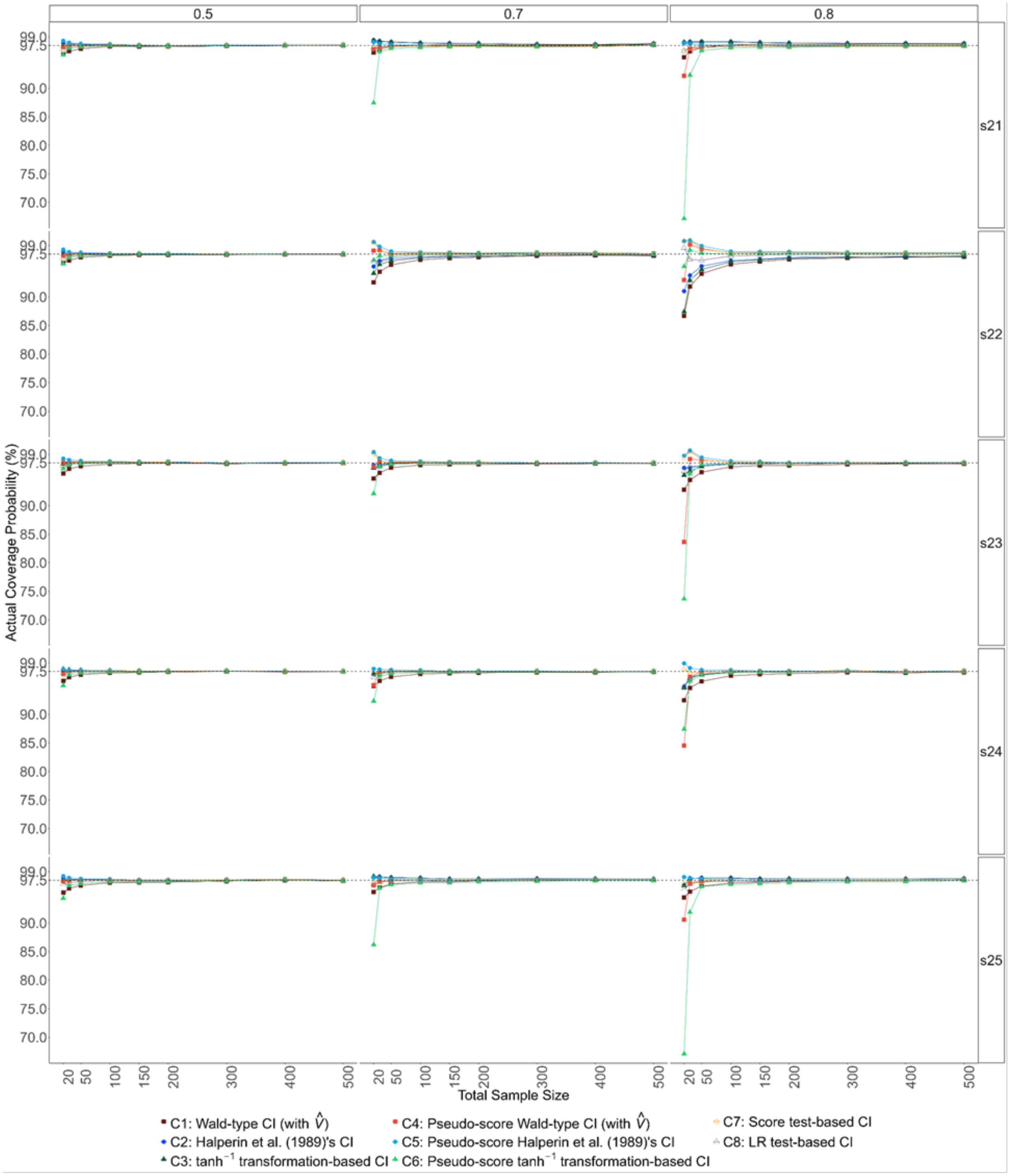

Figure A13. Behavior of the actual coverage probability of the one-sided 97.5% lower CI estimate with varying sample sizes, sample size allocation, and the shape of the DOOR outcome distribution ($K = 5$; S21–S25)

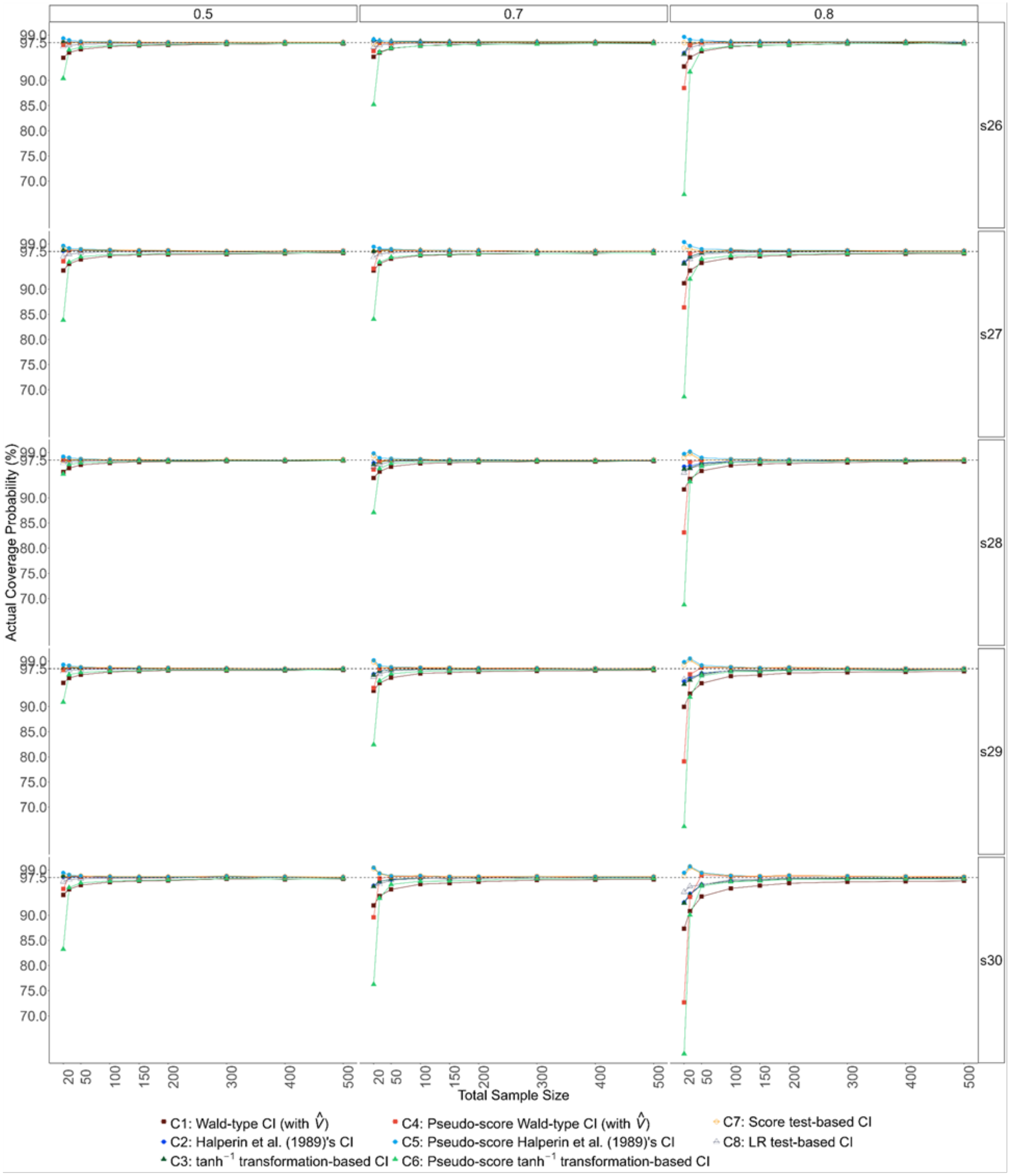

Figure A14. Behavior of the actual coverage probability of the one-sided 97.5% lower CI estimate with varying sample sizes, sample size allocation, and the shape of the DOOR outcome distribution ($K = 5$; S26–S30)

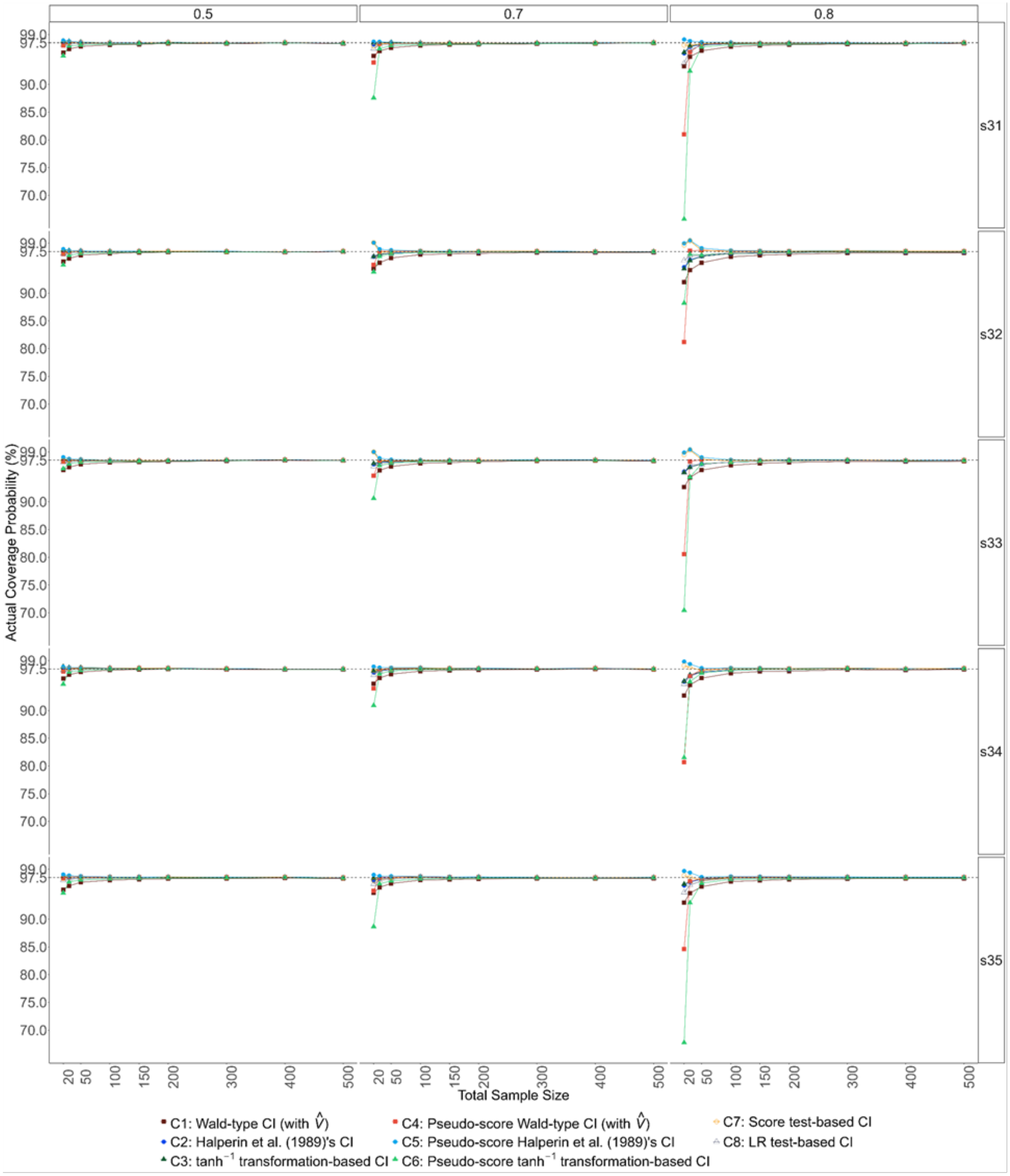

Figure A15. Behavior of the actual coverage probability of the one-sided 97.5% lower CI estimate with varying sample sizes, sample size allocation, and the shape of the DOOR outcome distribution ($K = 9$; S31 – S35)

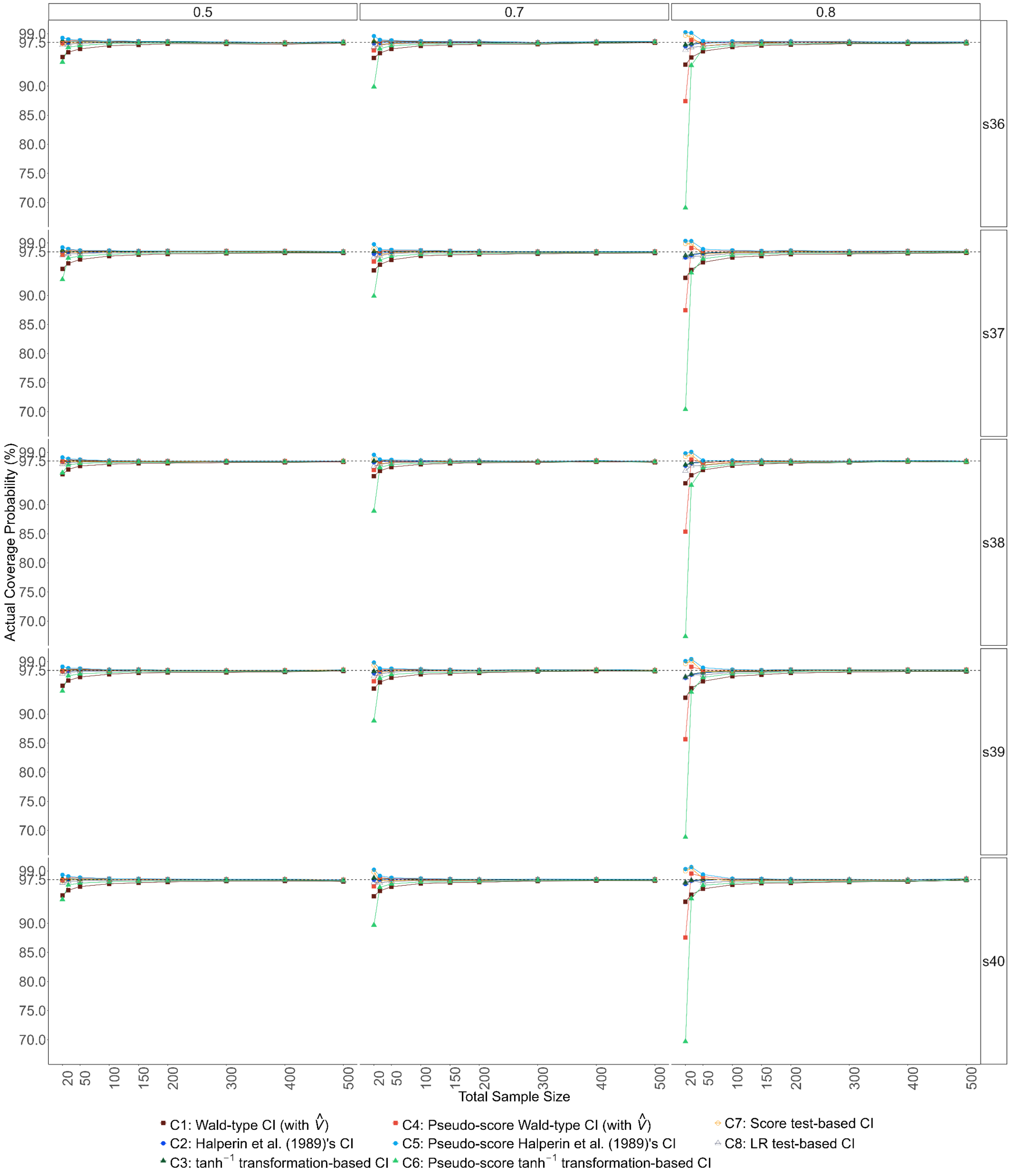

Figure A16. Behavior of the actual coverage probability of the one-sided 97.5% lower CI estimate with varying sample sizes, sample size allocation, and the shape of the DOOR outcome distribution ($K = 9$; S36– S40)

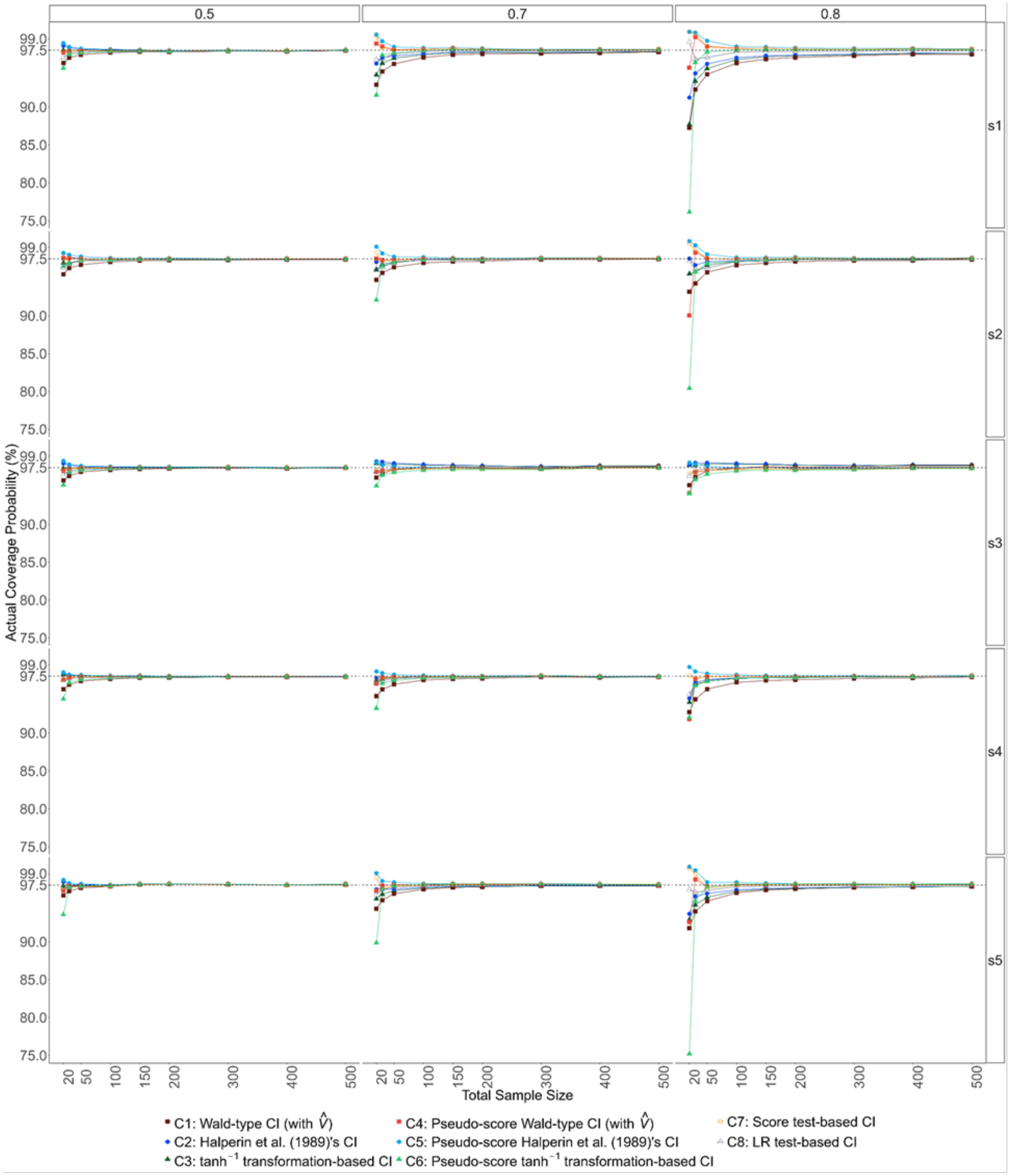

Figure A17. Behavior of the actual coverage probability of the one-sided 97.5% upper CI estimate with varying sample sizes, sample size allocation, and the shape of the DOOR outcome distribution ($K = 3$; S1–S5)

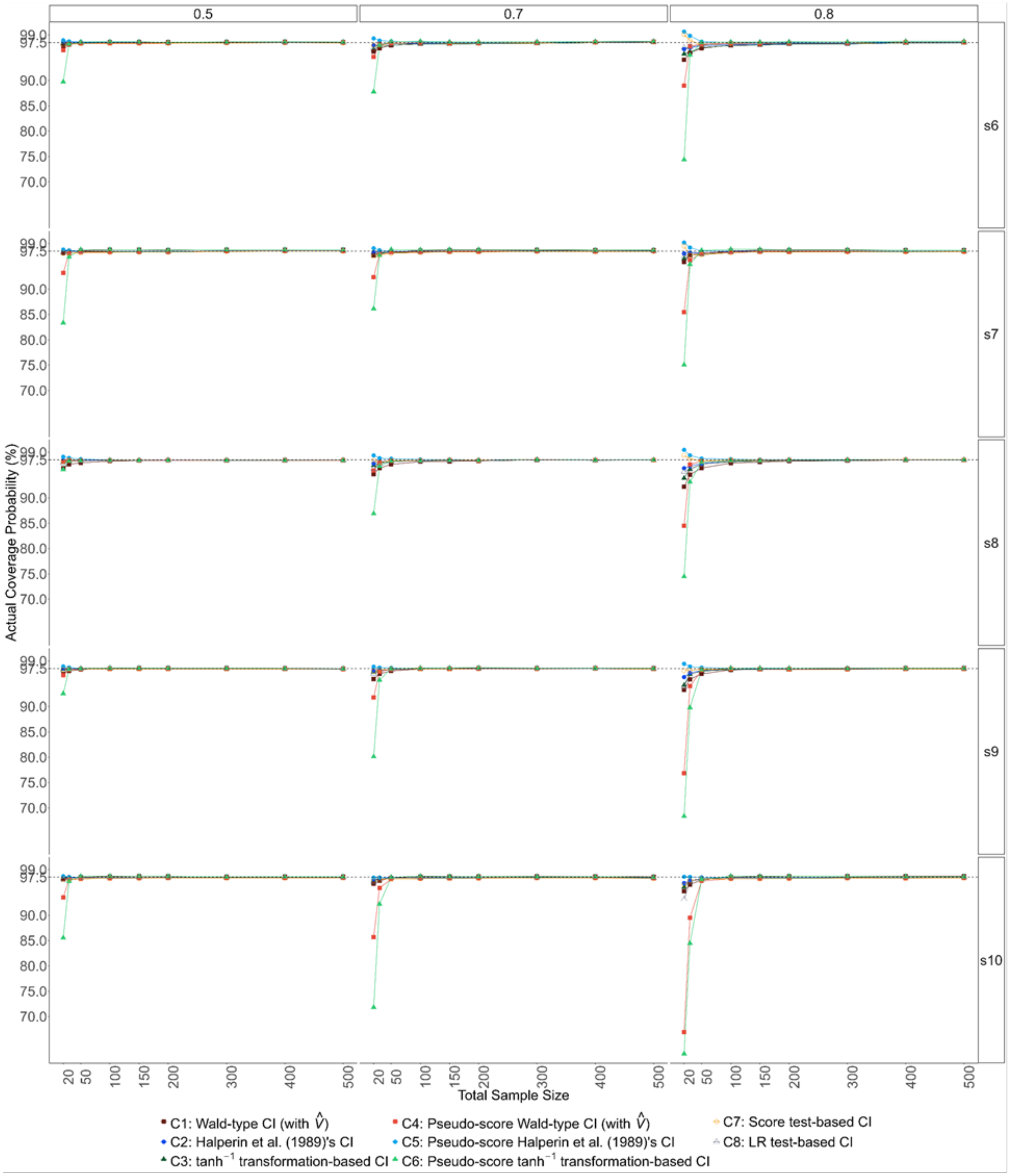

Figure A18. Behavior of the actual coverage probability of the one-sided 97.5% upper CI estimate with varying sample sizes, sample size allocation, and the shape of the DOOR outcome distribution ($K = 3$; S6–S10)

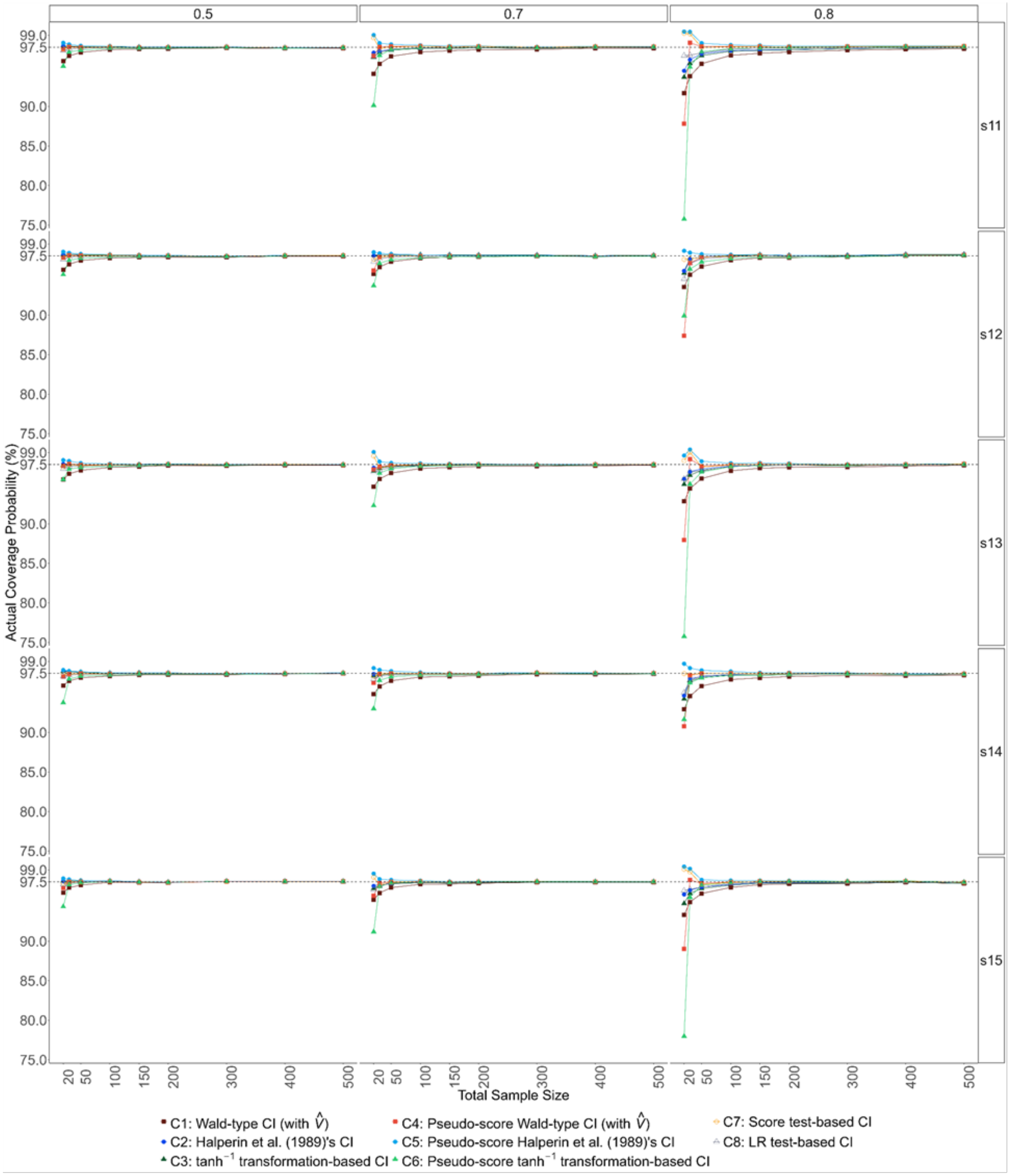

Figure A19. Behavior of the actual coverage probability of the one-sided 97.5% upper CI estimate with varying sample sizes, sample size allocation, and the shape of the DOOR outcome distribution ($K = 4$; S11–S15)

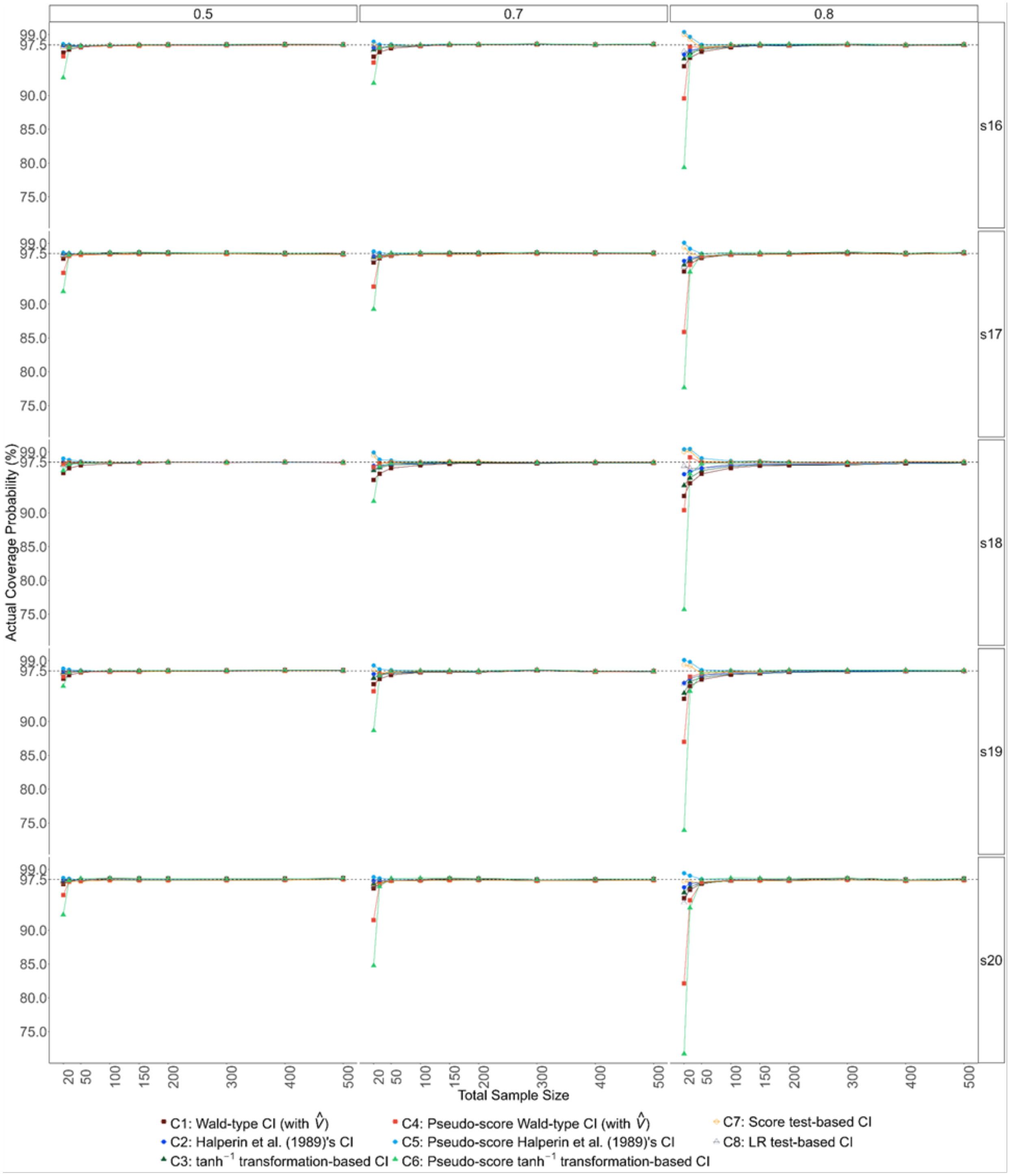

Figure A20. Behavior of the actual coverage probability of the one-sided 97.5% upper CI estimate with varying sample sizes, sample size allocation, and the shape of the DOOR outcome distribution ($K = 4$; S16–S20)

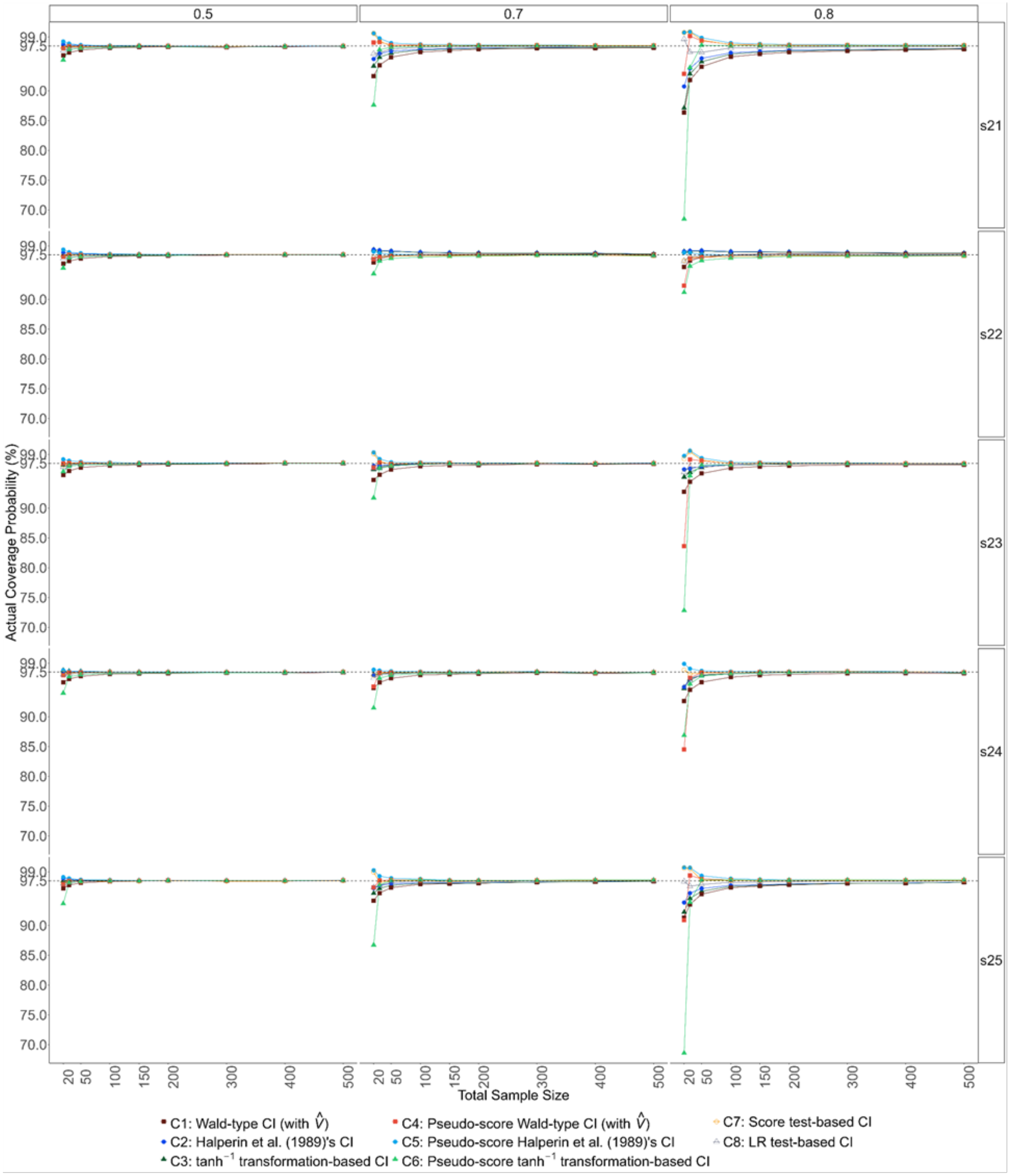

Figure A21. Behavior of the actual coverage probability of the one-sided 97.5% upper CI estimate with varying sample sizes, sample size allocation, and the shape of the DOOR outcome distribution ($K = 5$; S21–S25)

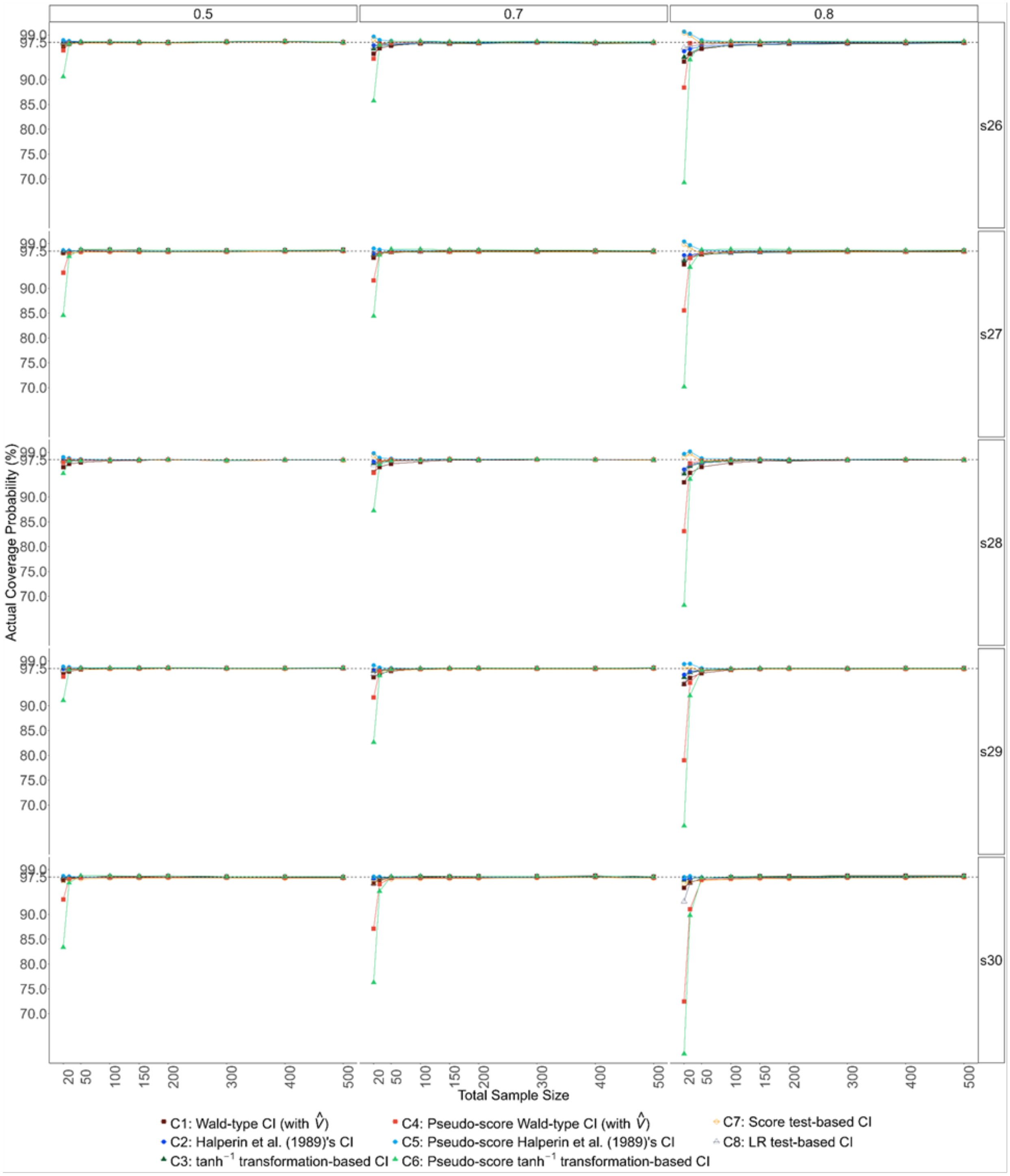

Figure A22. Behavior of the actual coverage probability of the one-sided 97.5% upper CI estimate with varying sample sizes, sample size allocation, and the shape of the DOOR outcome distribution ($K = 5$; S26–S30)

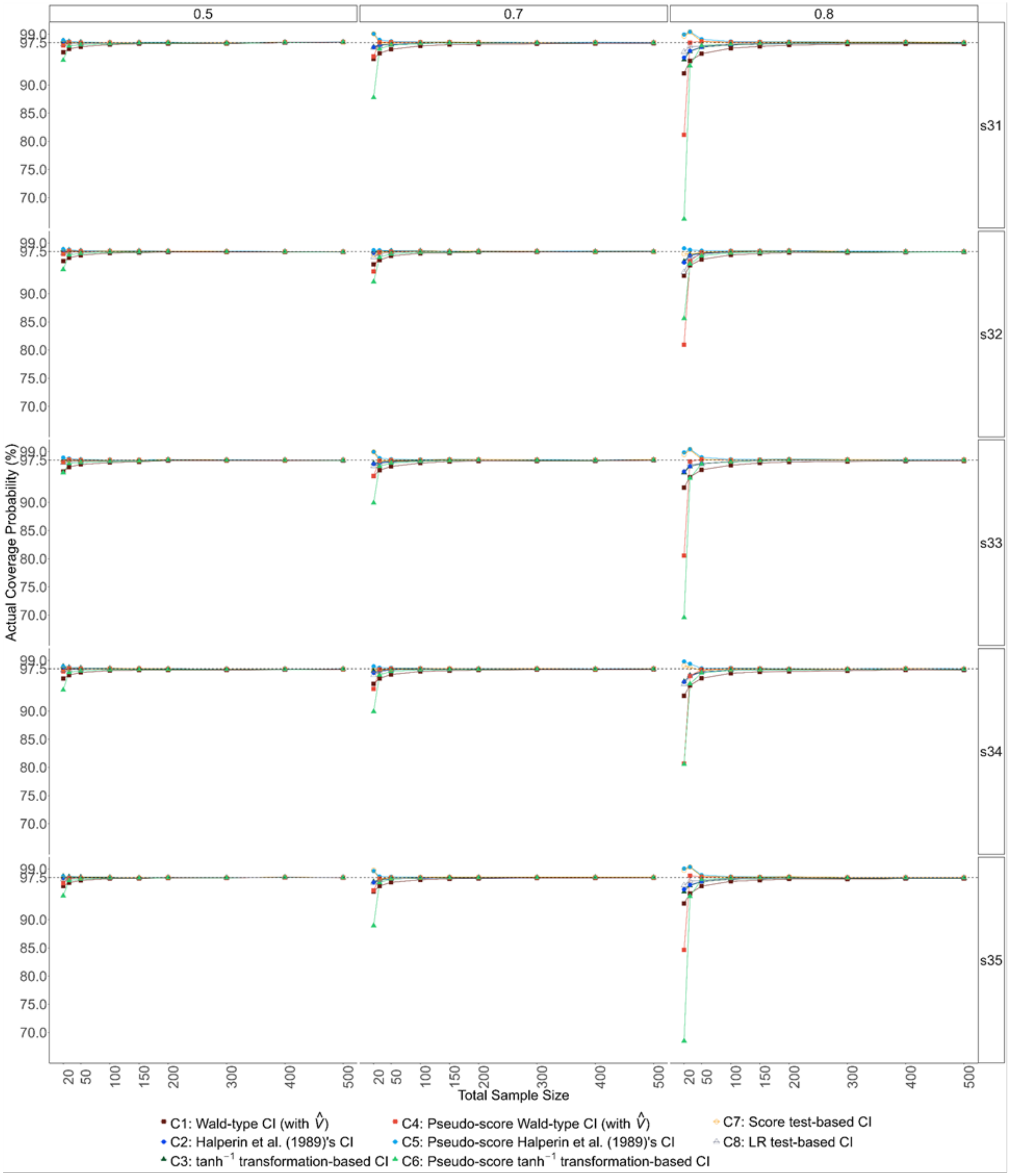

Figure A23. Behavior of the actual coverage probability of the one-sided 97.5% upper CI estimate with varying sample sizes, sample size allocation, and the shape of the DOOR outcome distribution ($K = 9$; S31–S35)

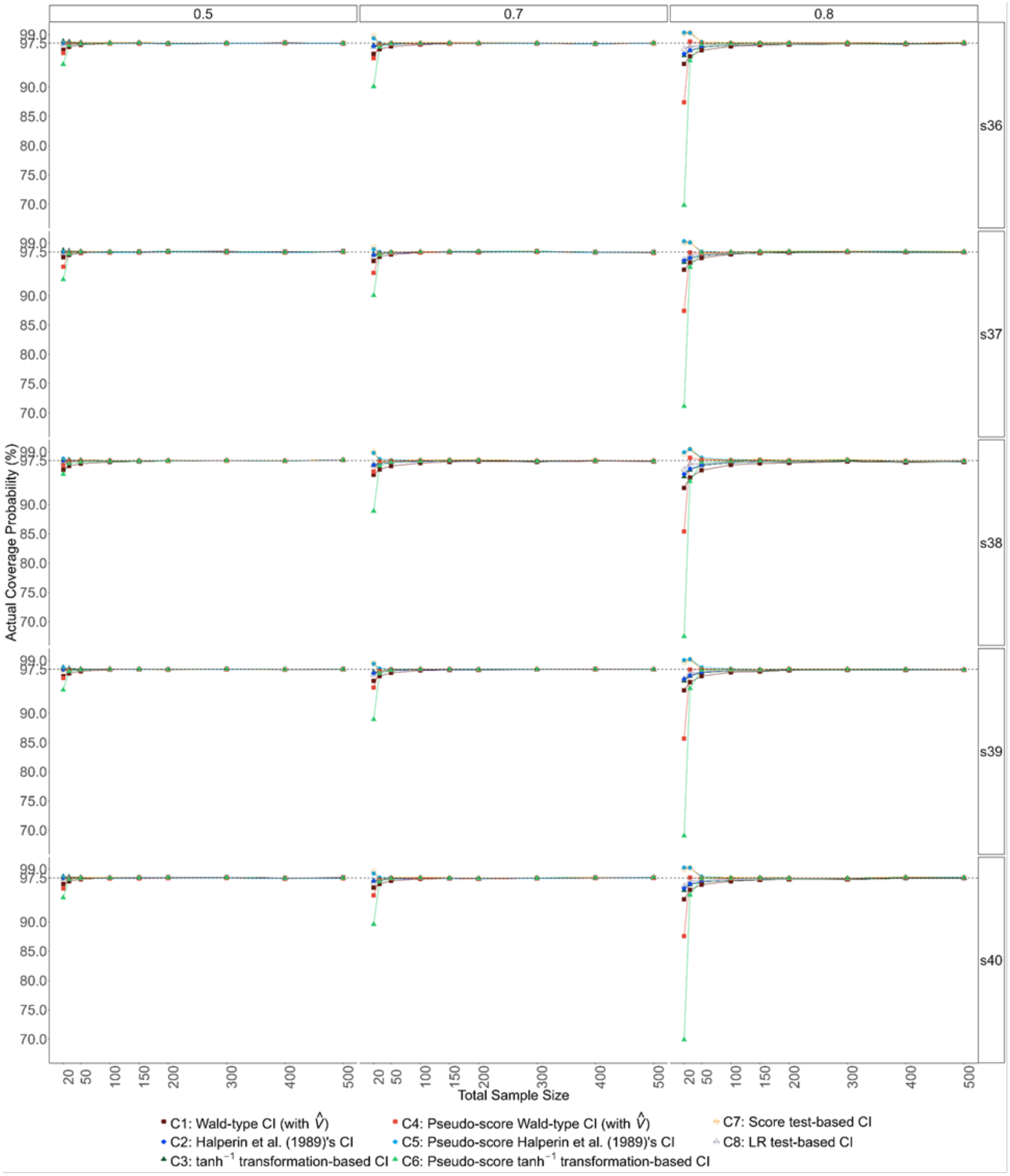

Figure A24. Behavior of the actual coverage probability of the one-sided 97.5% upper CI estimate with varying sample sizes, sample size allocation, and the shape of the DOOR outcome distribution ($K = 9$; S36–S40)

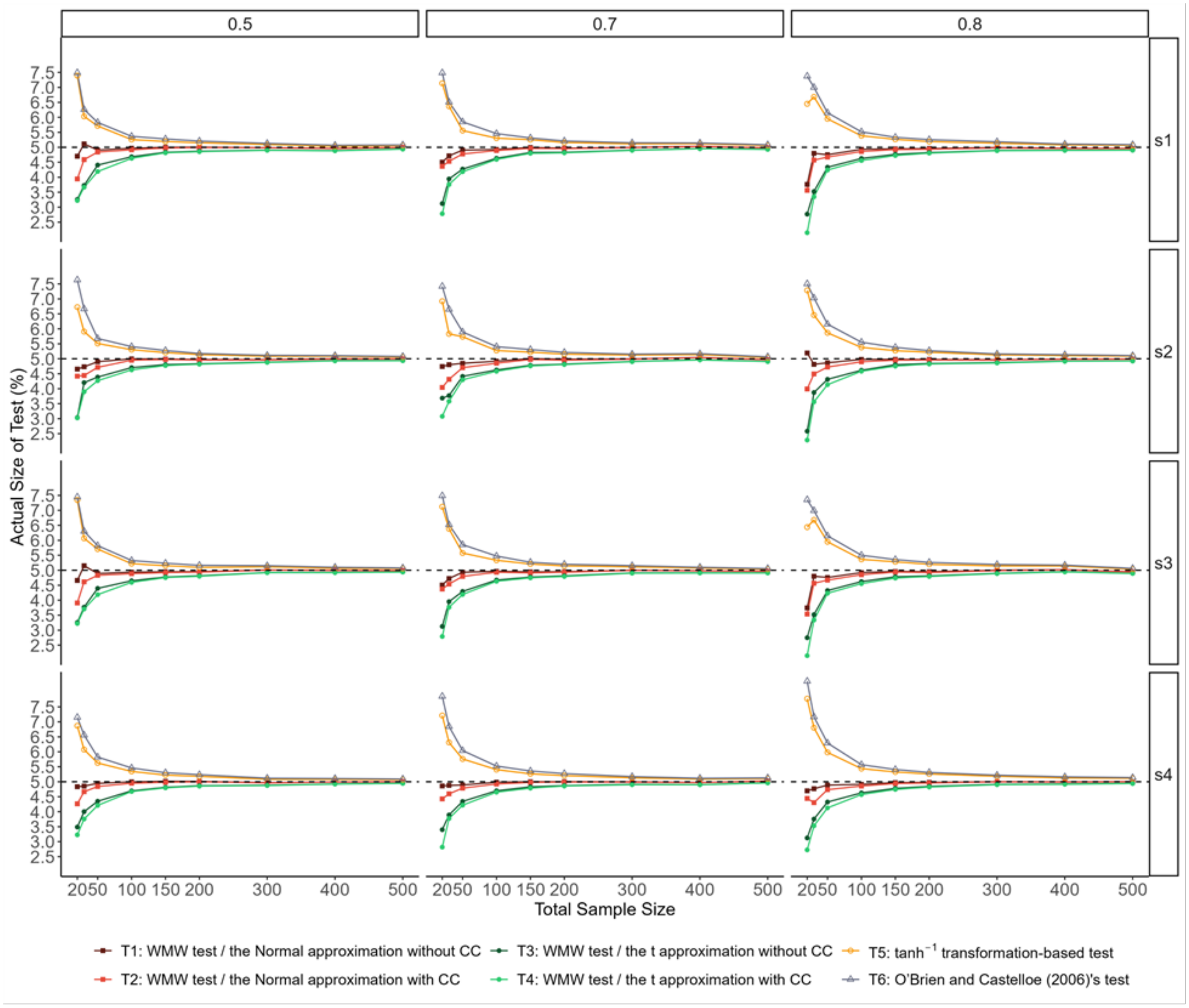

Figure A25. Behavior of the actual size of the two-sided hypothesis test evaluated at 5% significance level with varying total sample size and sample size allocation ($K = 3$; S1–S4)

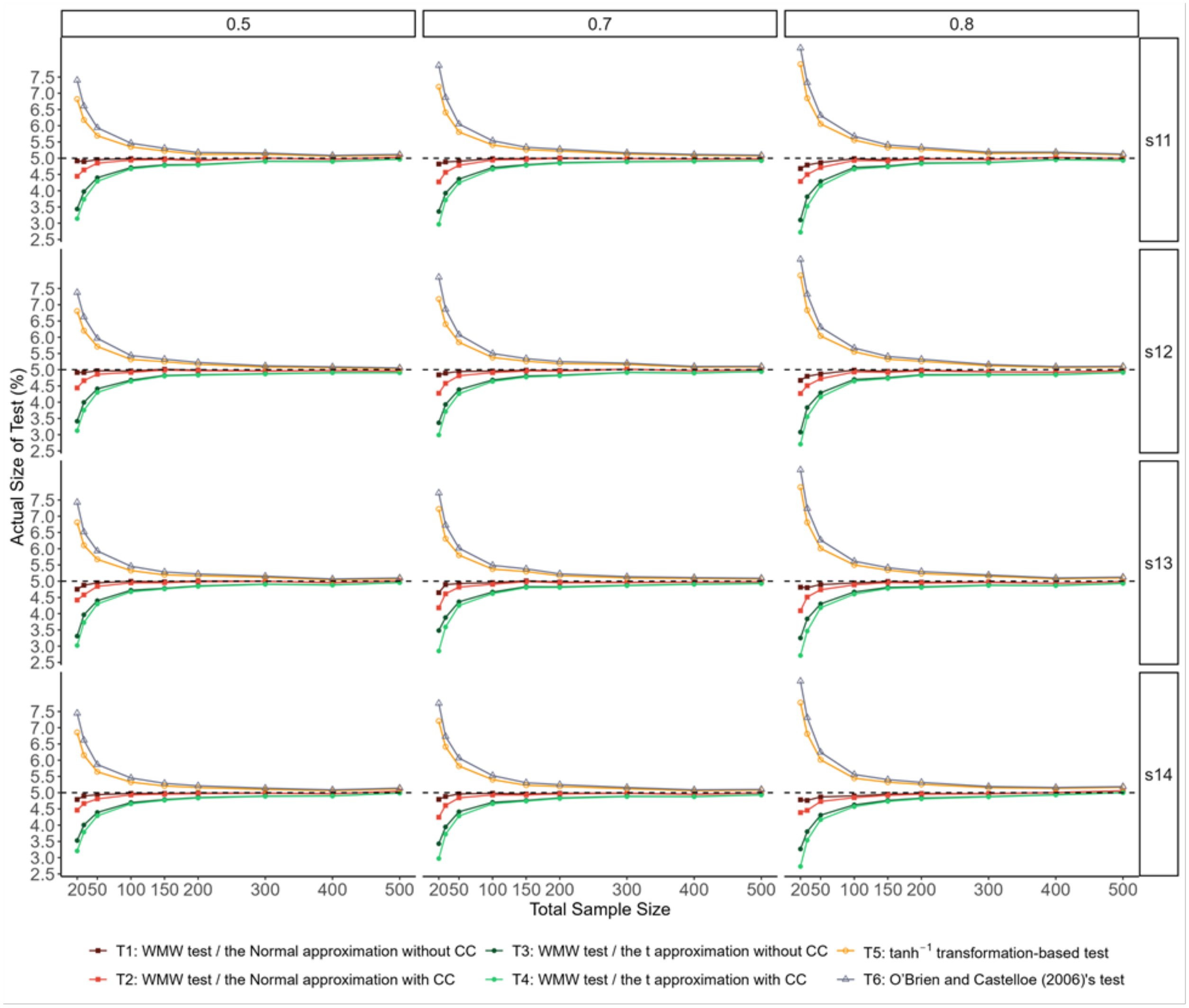

Figure A26. Behavior of the actual size of the two-sided hypothesis test evaluated at 5% significance level with varying total sample size and sample size allocation ($K = 4$; S11–S14)

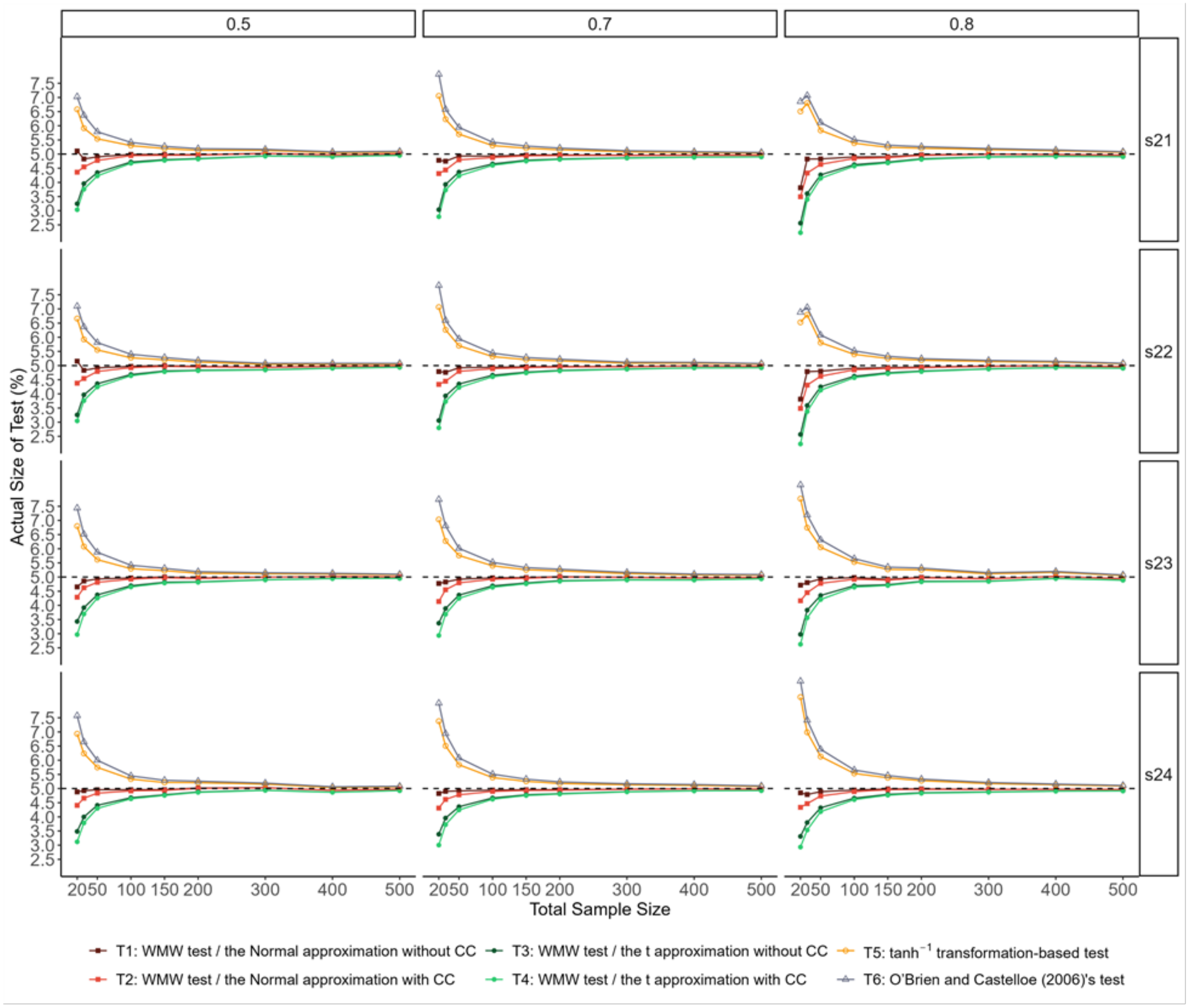

Figure A27. Behavior of the actual size of the two-sided hypothesis test evaluated at 5% significance level with varying total sample size and sample size allocation ($K = 5$; S20–S24)

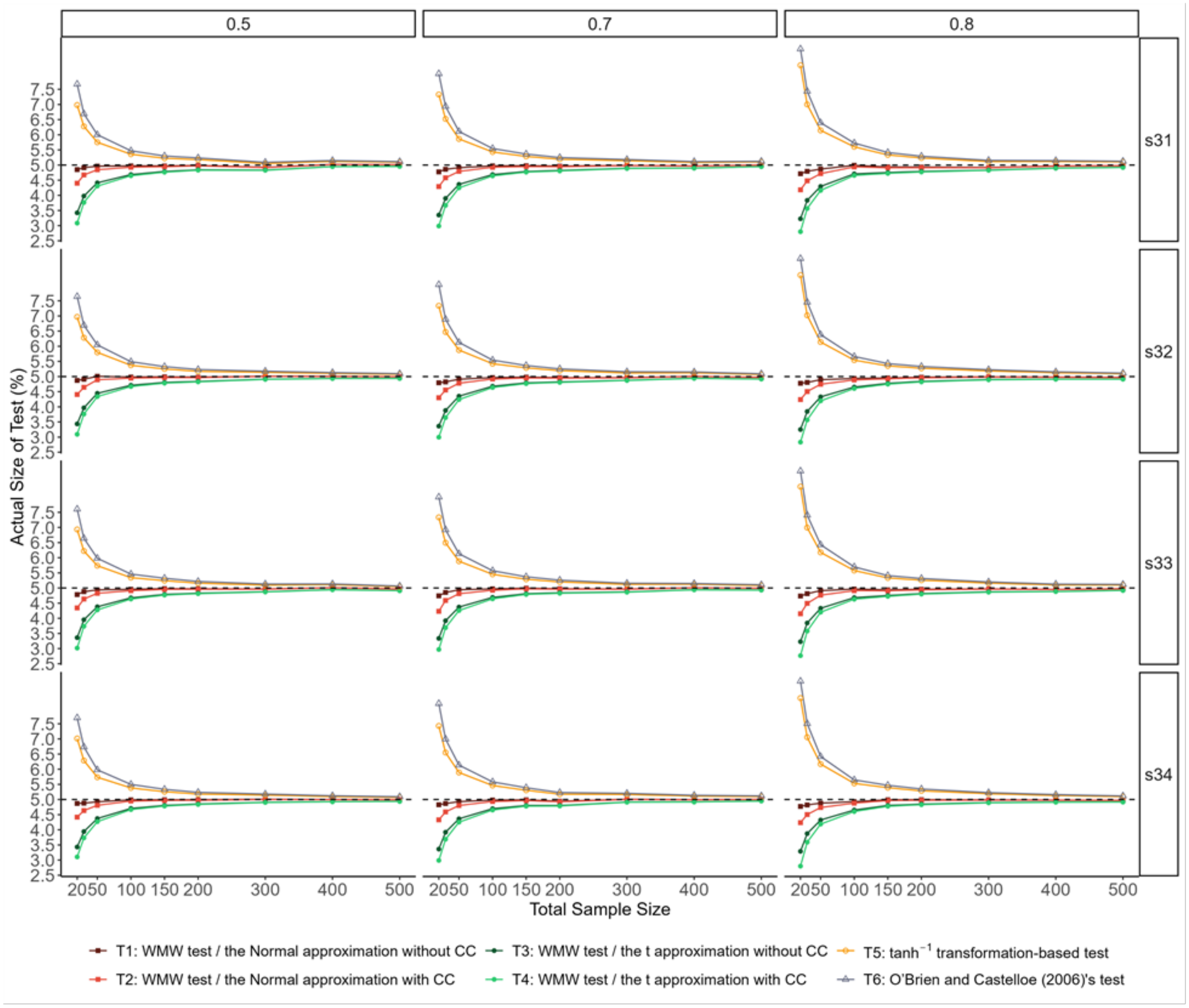

Figure A28. Behavior of the actual size of the two-sided hypothesis test evaluated at 5% significance level with varying total sample size and sample size allocation ($K = 9$; S31–S34)

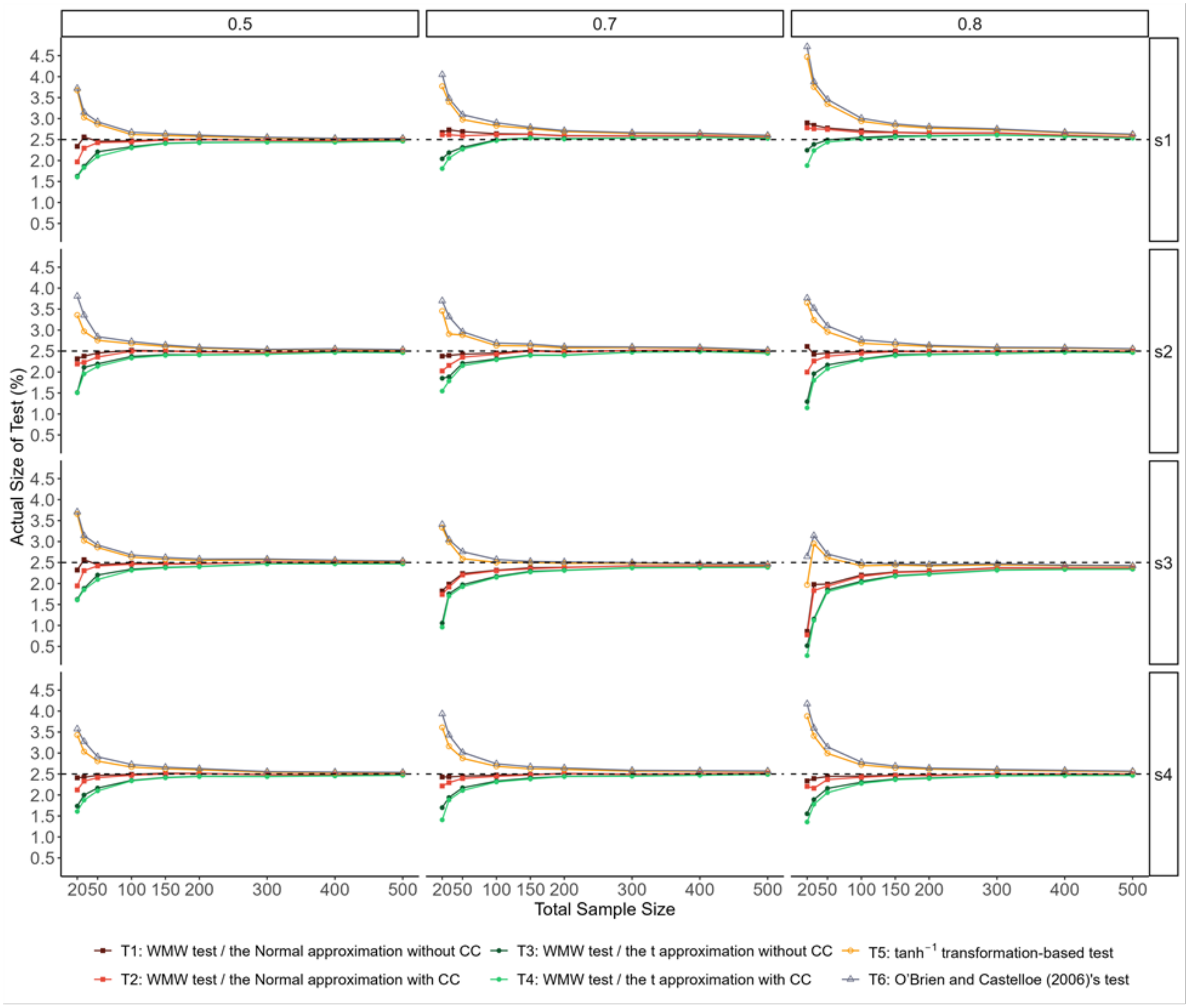

Figure A29. Behavior of the actual size (upper) of the one-sided hypothesis test evaluated at 2.5% significance level with varying total sample size and sample size allocation ($K = 3$; S1–S4)

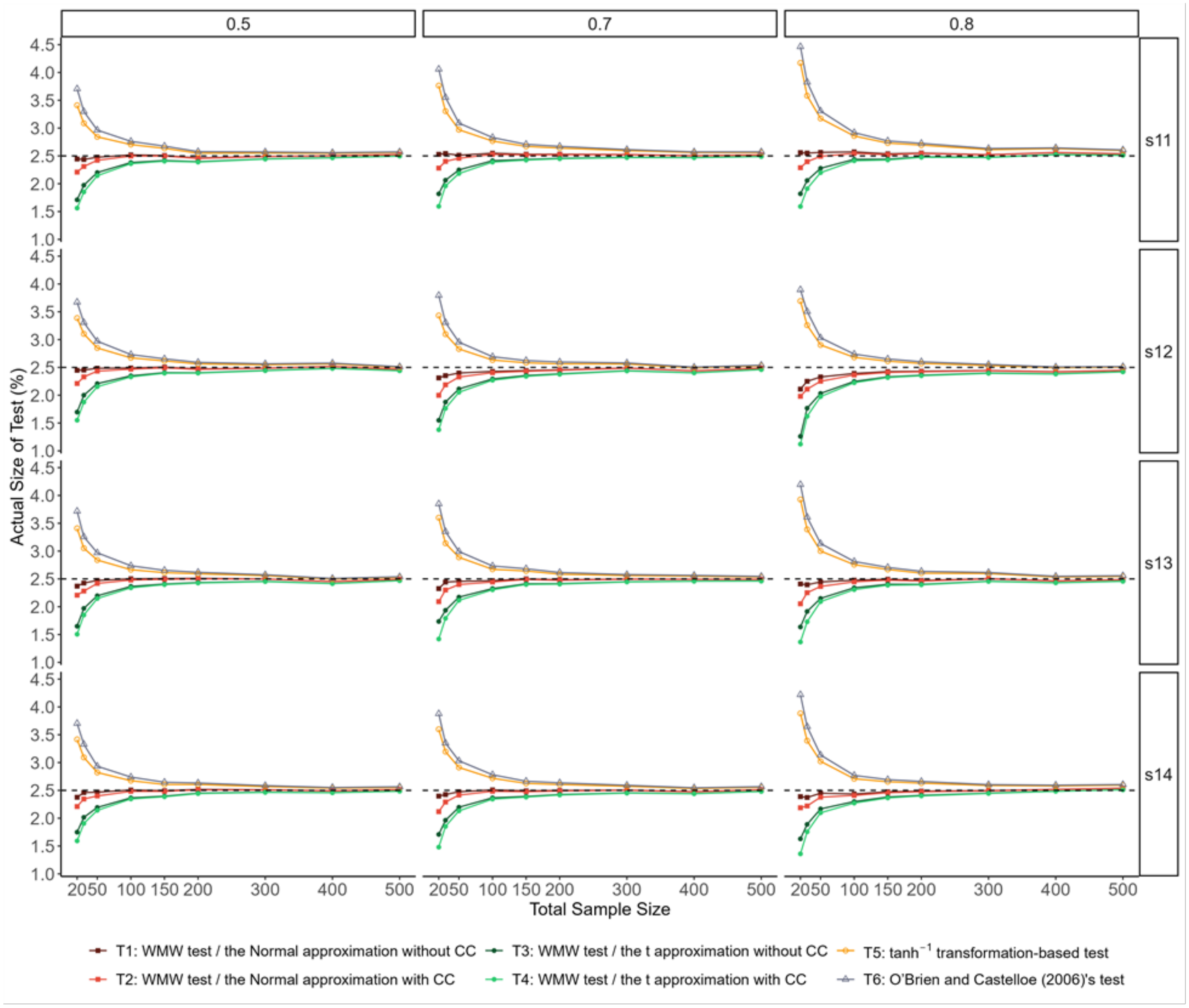

Figure A30. Behavior of the actual size (upper) of the one-sided hypothesis test evaluated at 2.5% significance level with varying total sample size and sample size allocation ($K = 4$; S10–S14)

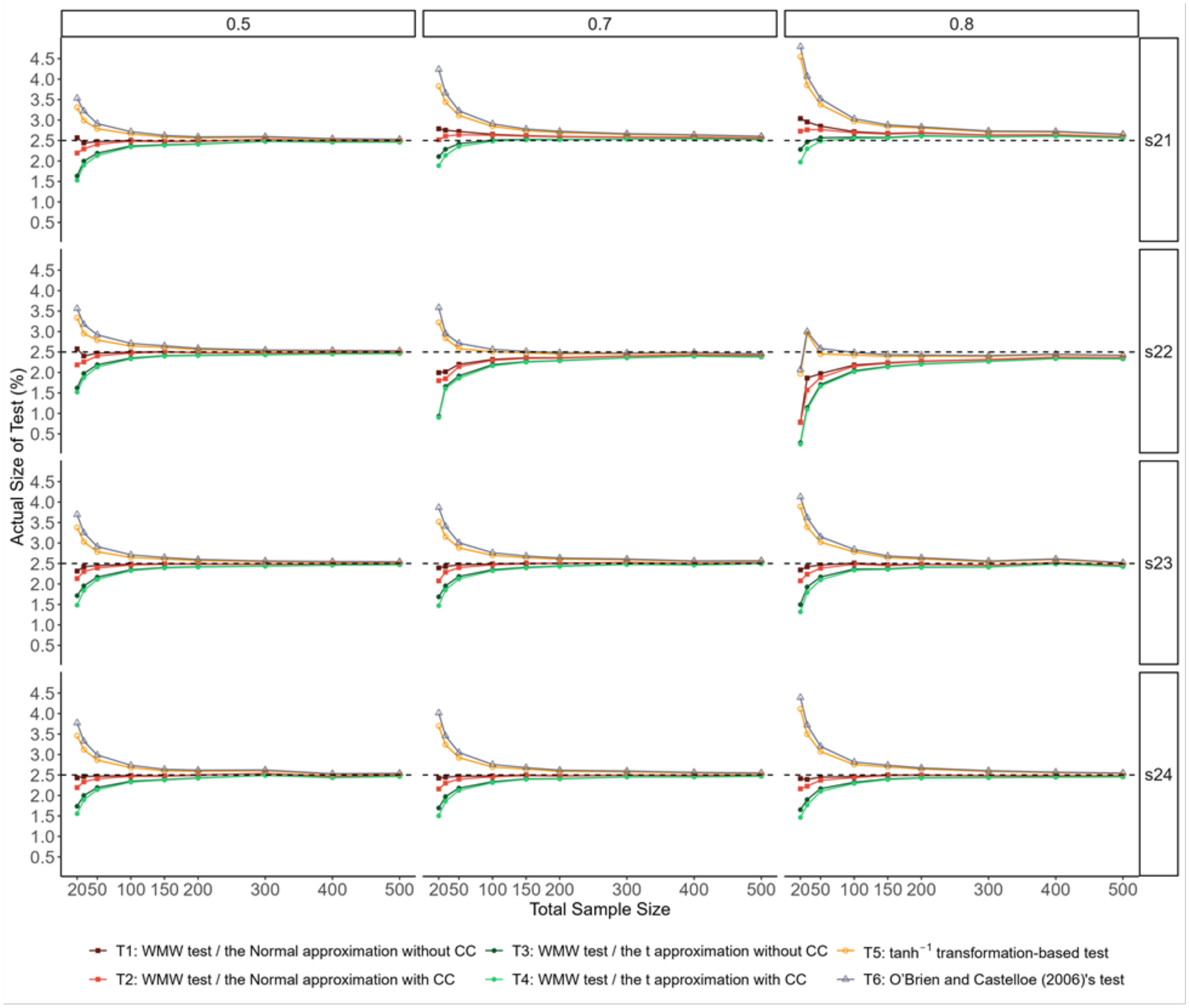

Figure A31. Behavior of the actual size (upper) of the one-sided hypothesis test evaluated at 2.5% significance level with varying total sample size and sample size allocation ($K = 5$; S21–S24)

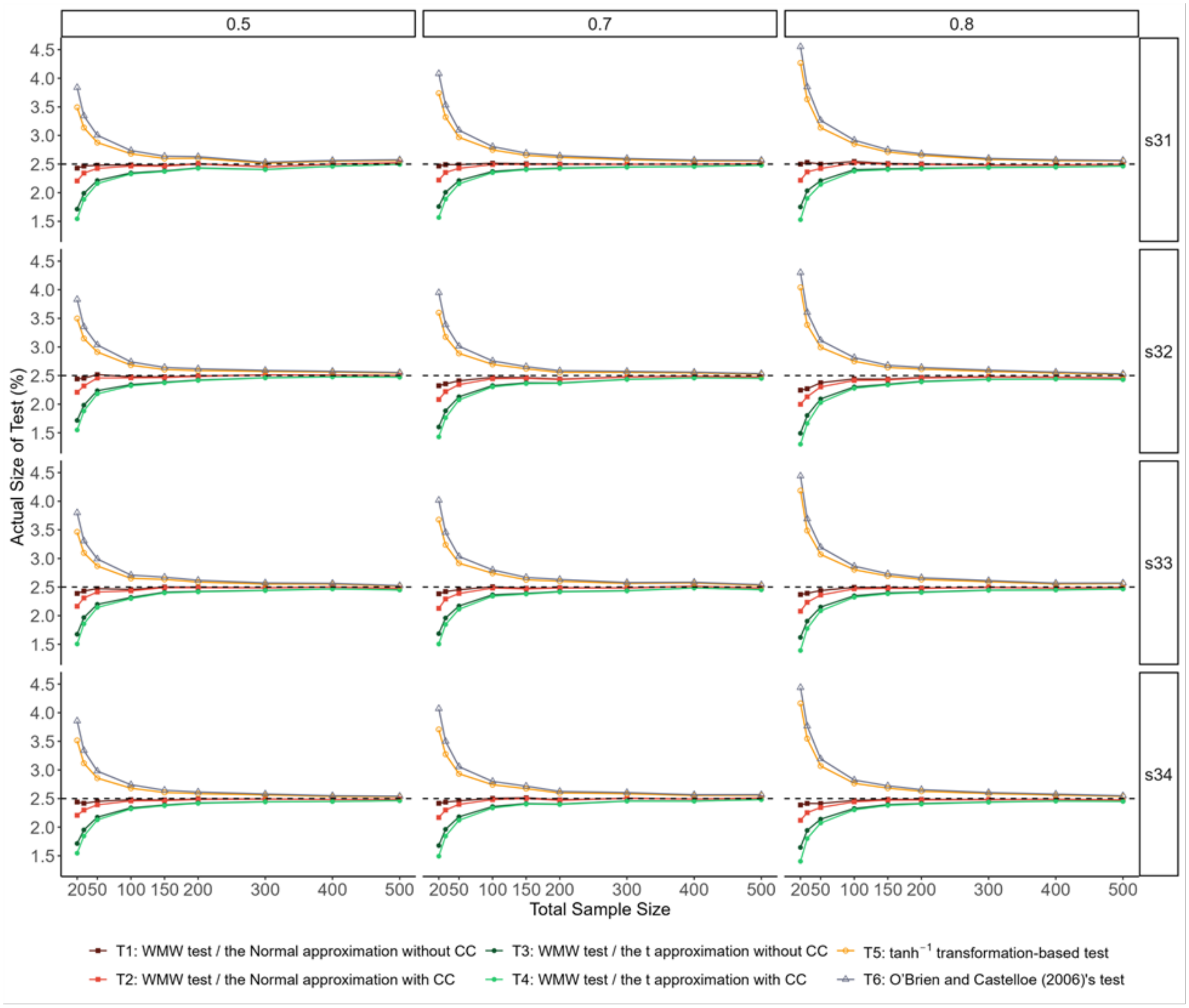

Figure A32. Behavior of the actual size (upper) of the one-sided hypothesis test evaluated at 2.5% significance level with varying total sample size and sample size allocation ($K = 9$; S31–S34)

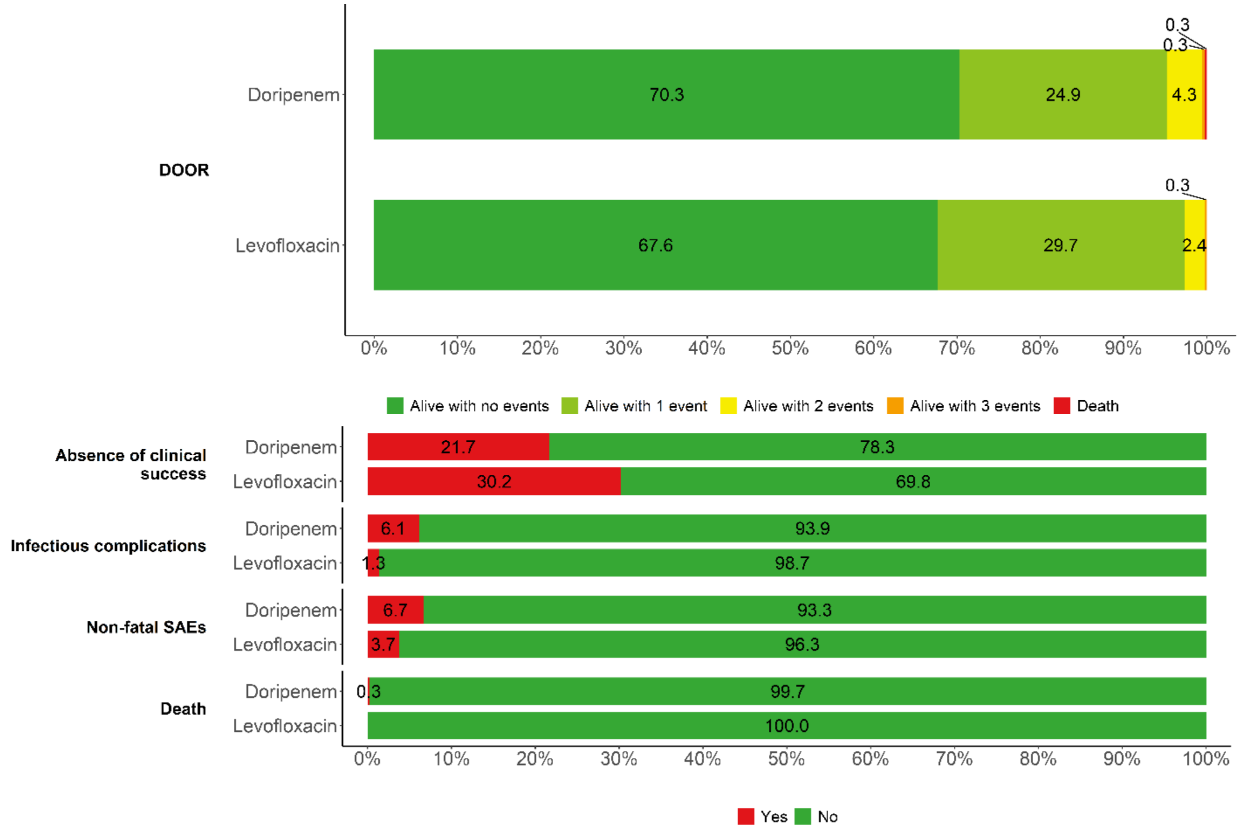

Figure A33. DORI-05: Bar chart of DOOR outcome distribution and respective DOOR components by treatment

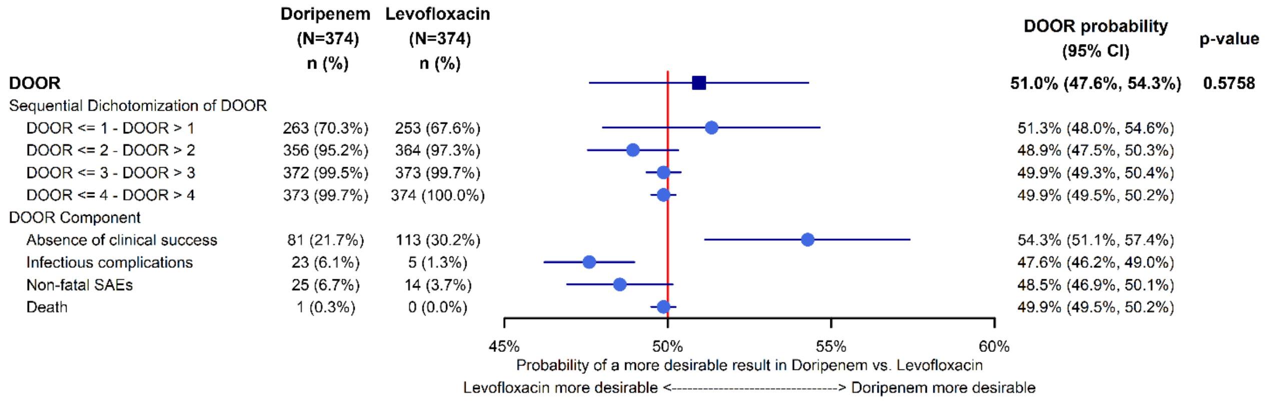

Figure A34. DORI-05: The difference of DOOR outcome and respective components between the two intervention groups

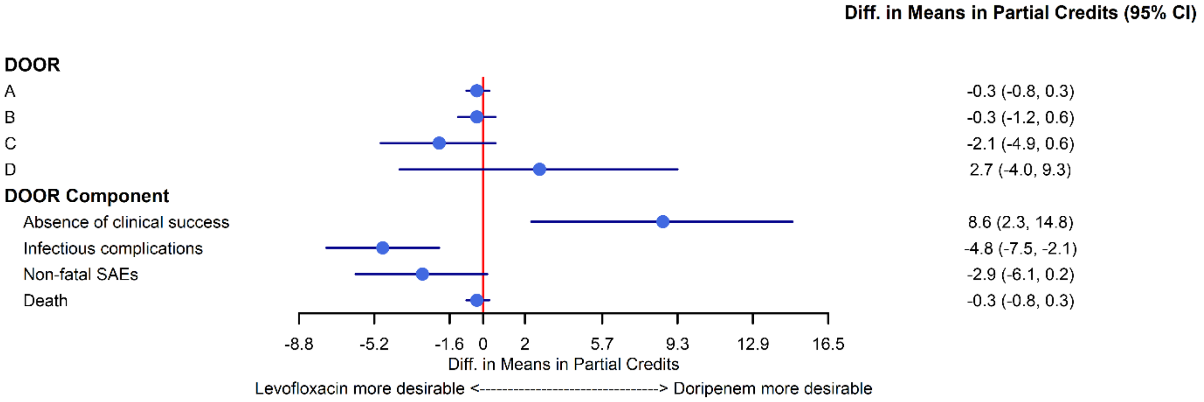

Figure A35. DORI-05: Forest of difference in means of partial credit for four grading keys. For the DOOR component, the "no" category was given 100 full credits and the "yes" category was given zero credits.

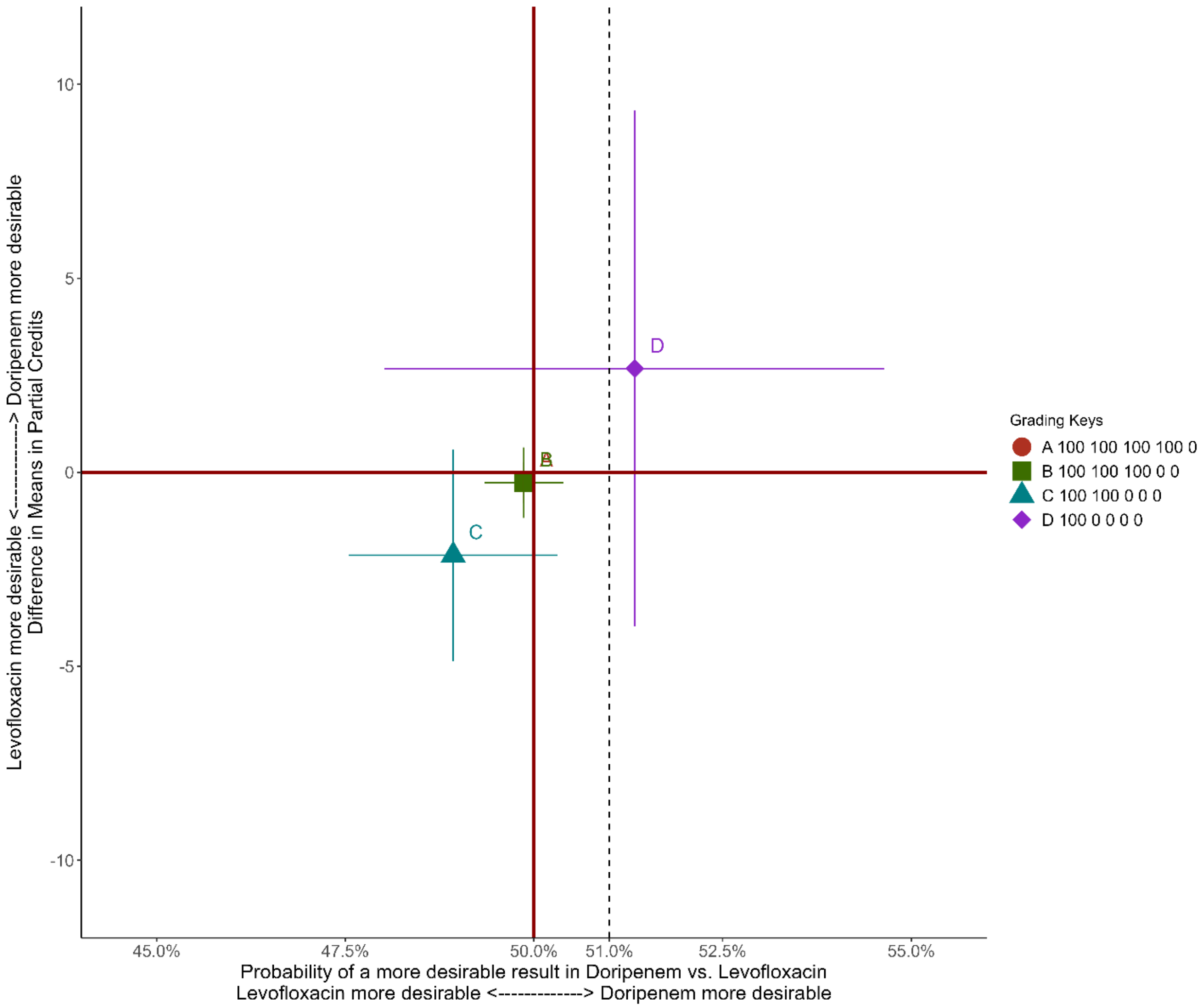

Figure A36. DORI-05: Plot of difference in means of partial credit vs. DOOR probability for four grading keys

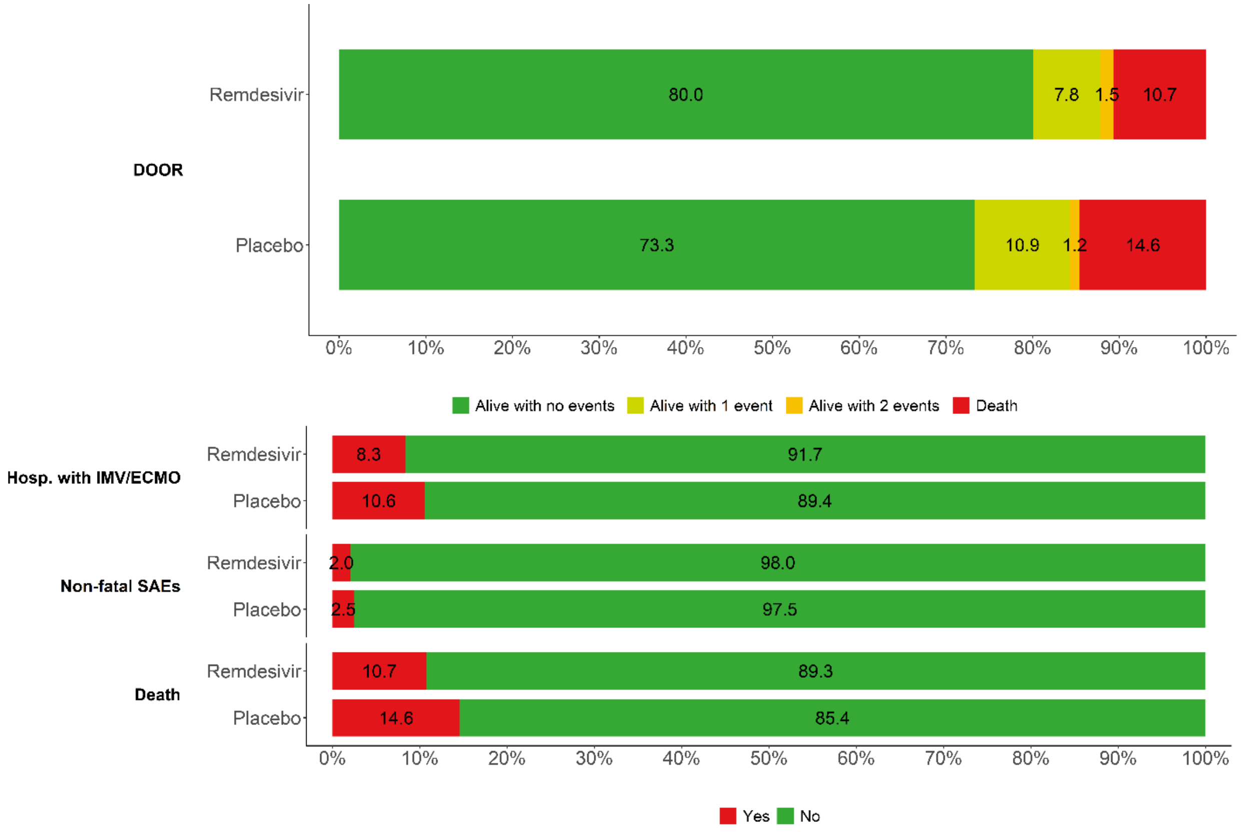

Figure A37. ACTT-1: Bar chart of DOOR outcome distribution and respective DOOR components by treatment

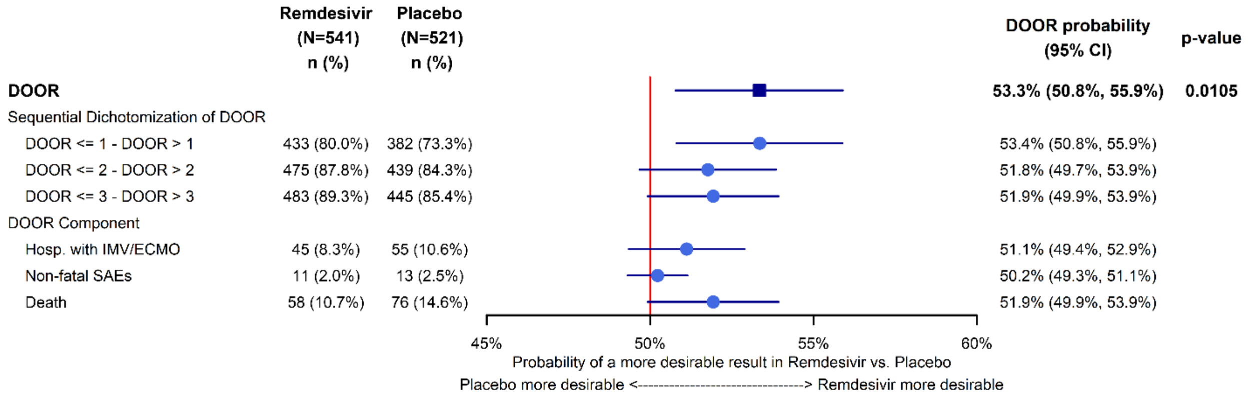

Figure A38. ACTT-1: The difference of DOOR outcome and respective components between the two intervention groups

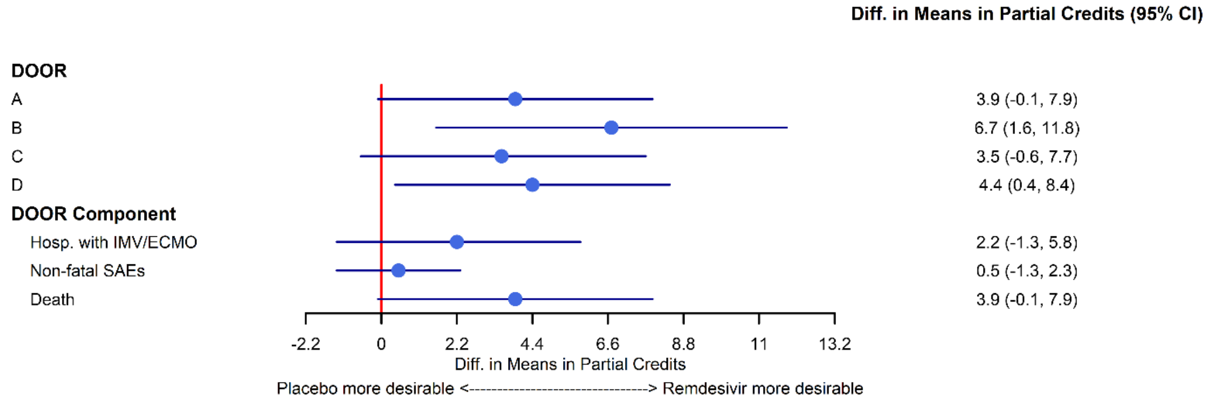

Figure A39. ACTT-1: Forest of difference in means of partial credit for four grading keys. For the DOOR component, the "no" category was given 100 full credits and the "yes" category was given zero credits.

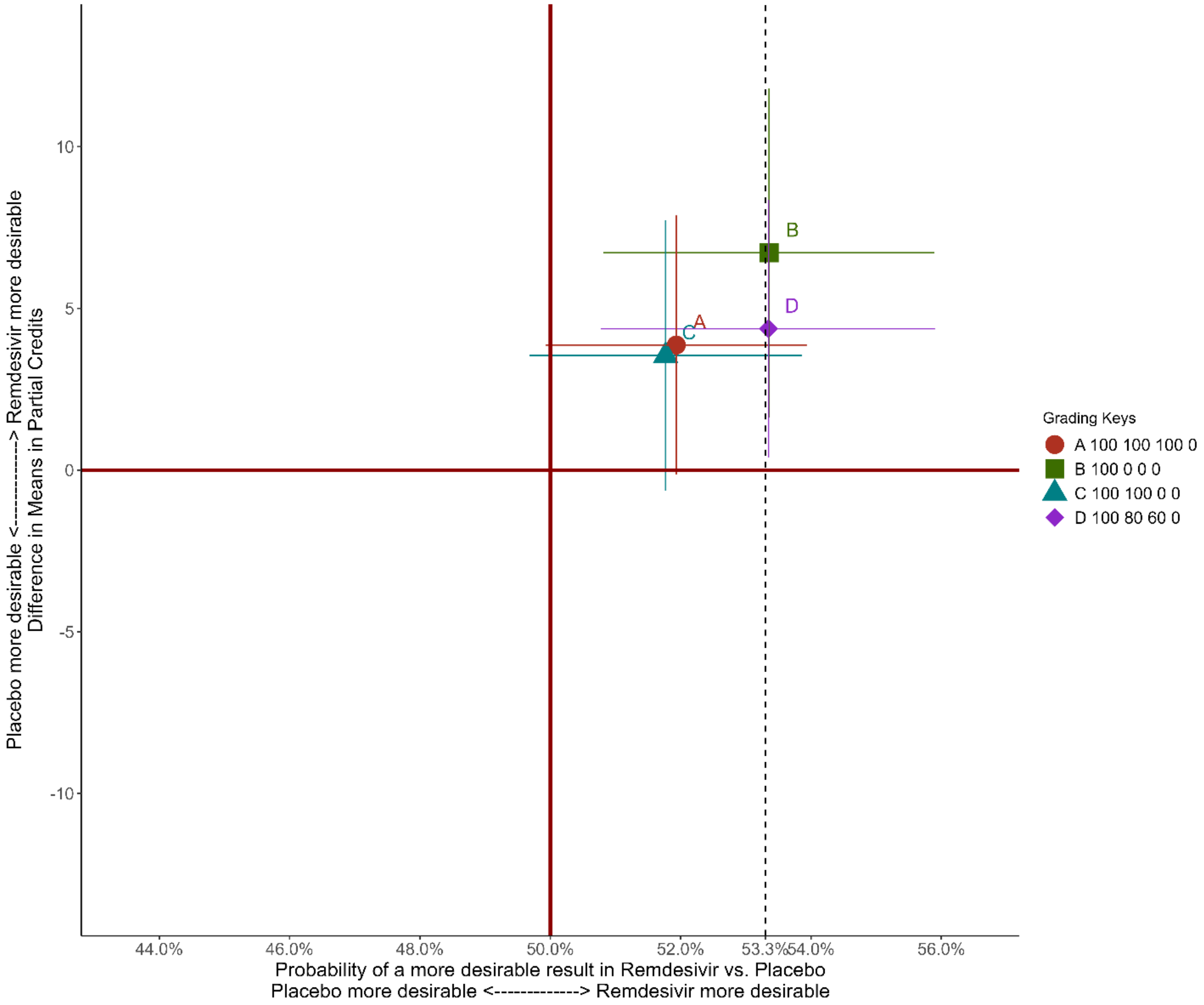

Figure A40. ACTT-1: Plot of difference in means of partial credit vs. DOOR probability for four grading keys

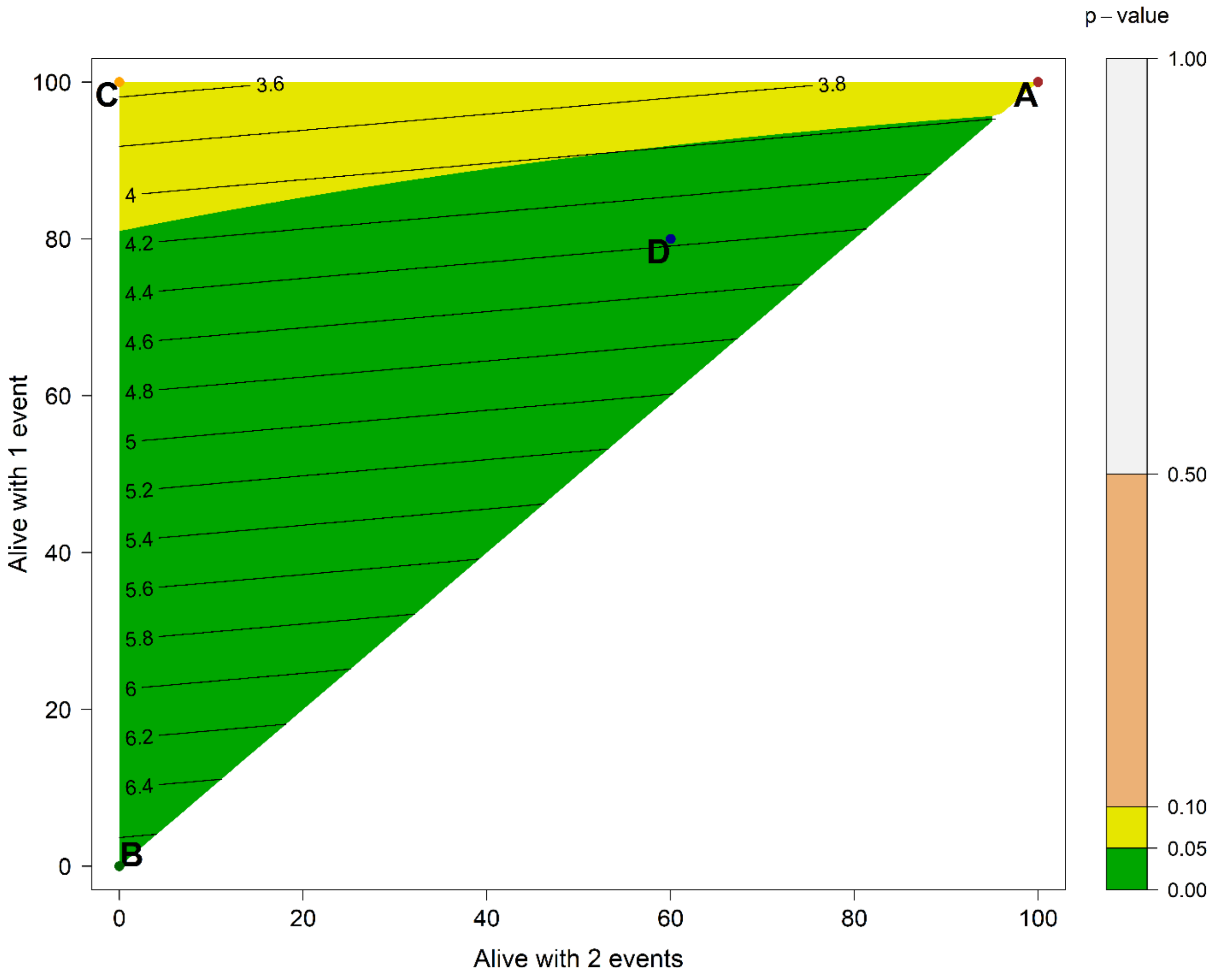

Figure A41. ACTT-1: Contour plots of the between-group difference in means as the partial credit assigned to DOOR outcome level two (alive with 1 event) and level three (alive with 2 events) vary. Green areas indicate grading key combinations that result in p-values less than 0.05; Positive numbers favor remdesivir and negative numbers favor placebo.

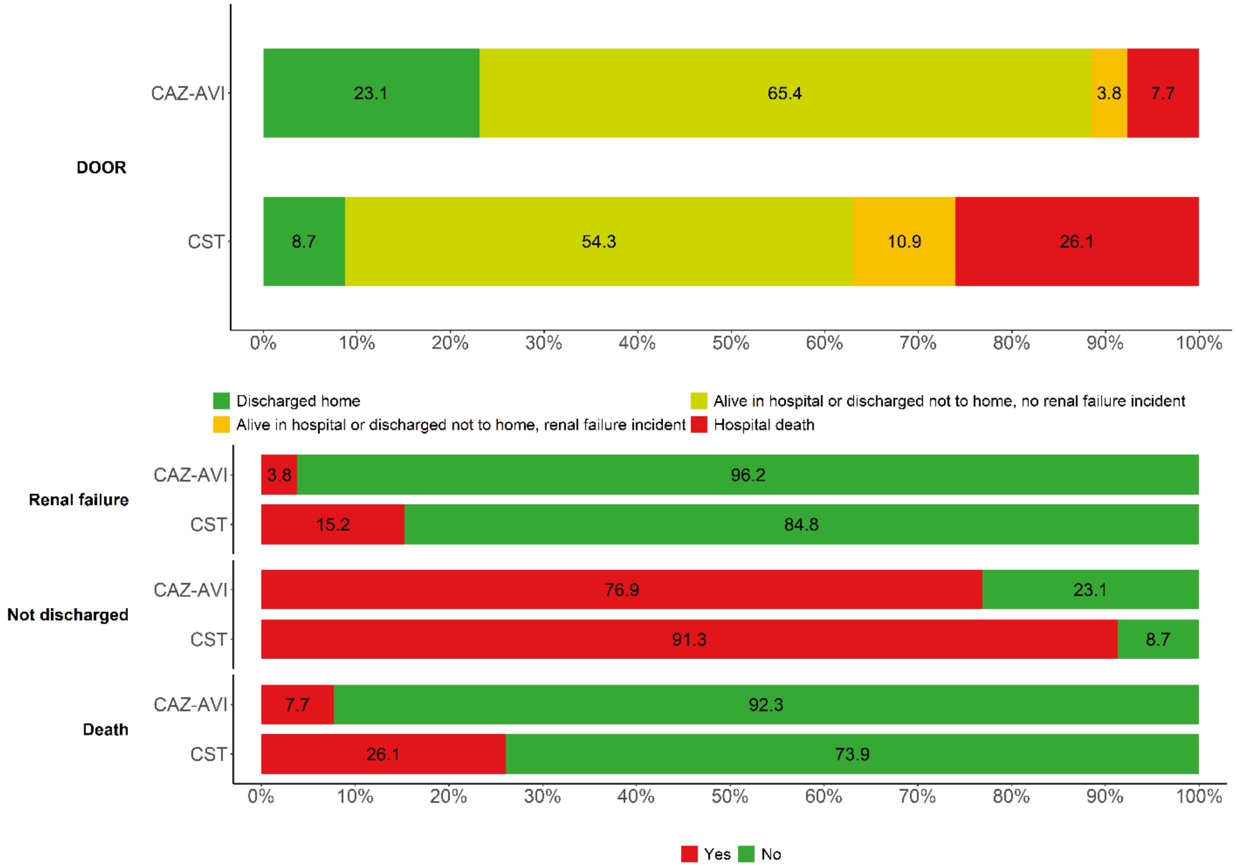

Figure A42. CRACKLE: Bar chart of DOOR outcome distribution and respective DOOR components by treatment (unadjusted)

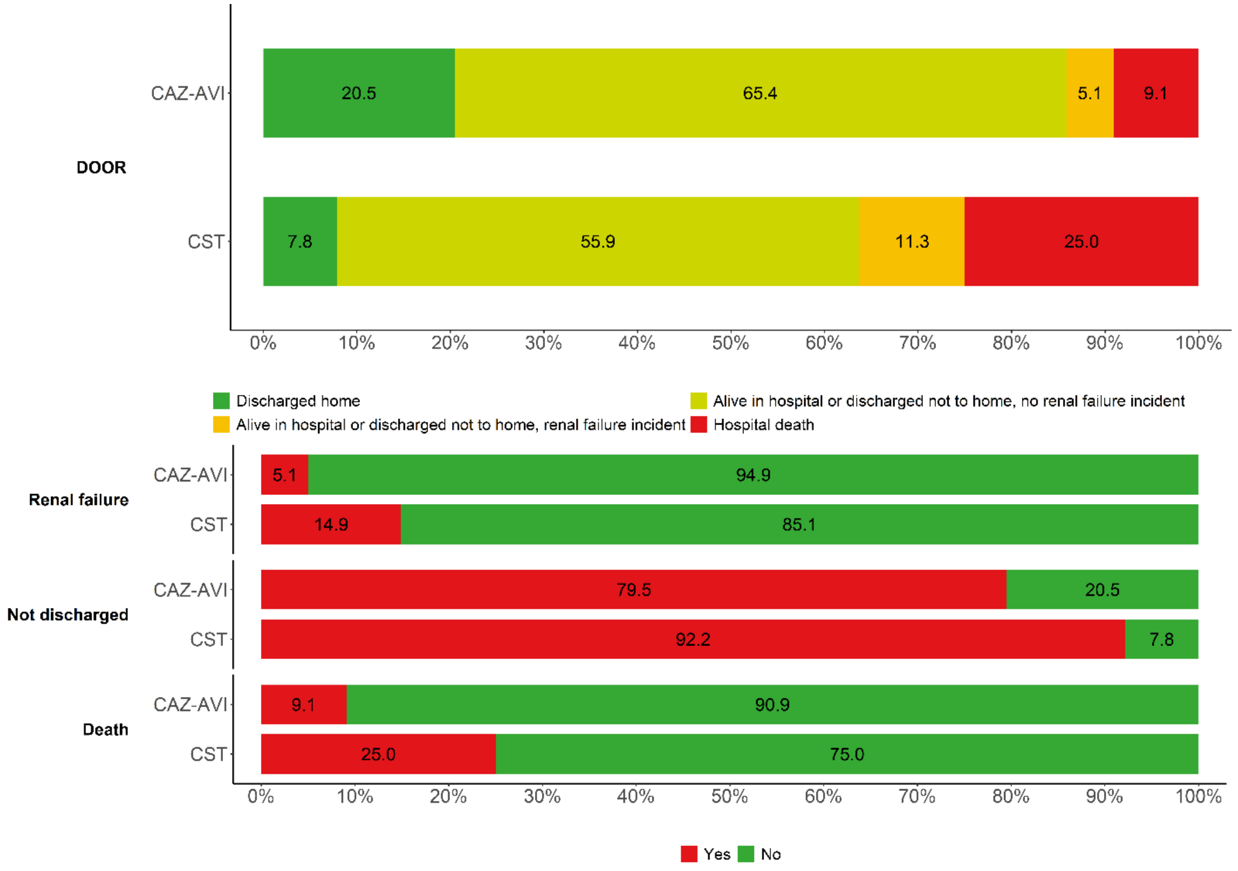

Figure A43. CRACKLE: Bar chart of DOOR outcome distribution and respective DOOR components by treatment (adjusted)

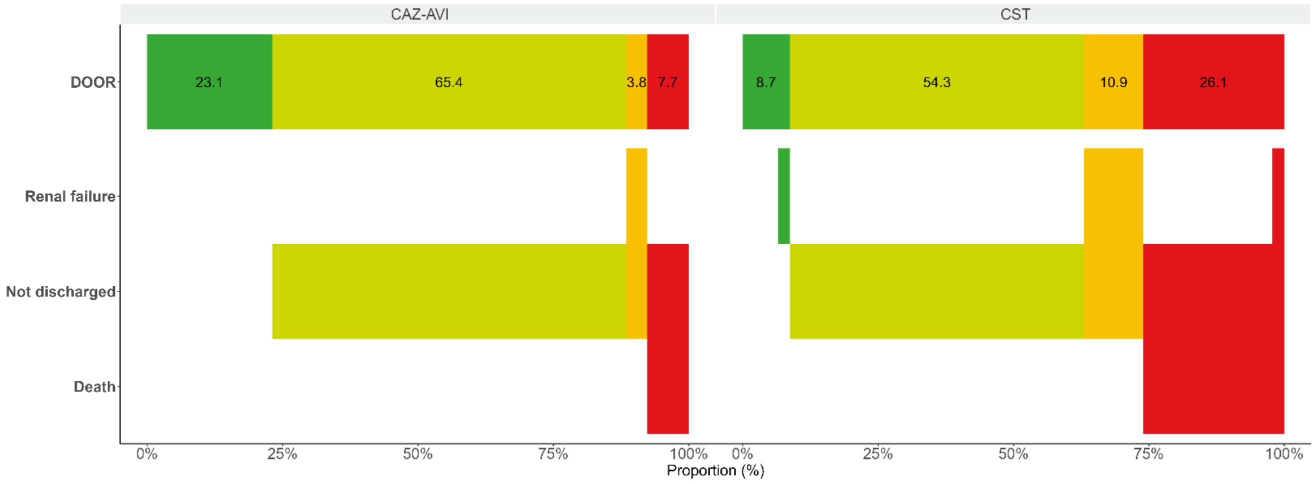

Figure A44. CRACKLE: Anthology of Patient Stories (APS) plot by treatment (unadjusted)

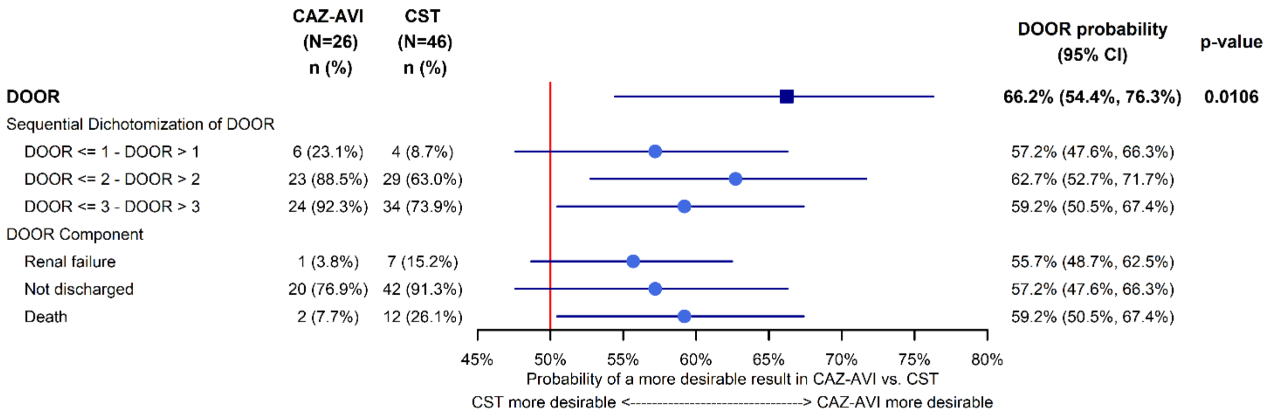

Figure A45. CRACKLE: The difference of DOOR outcome and respective components between the two intervention groups (unadjusted)

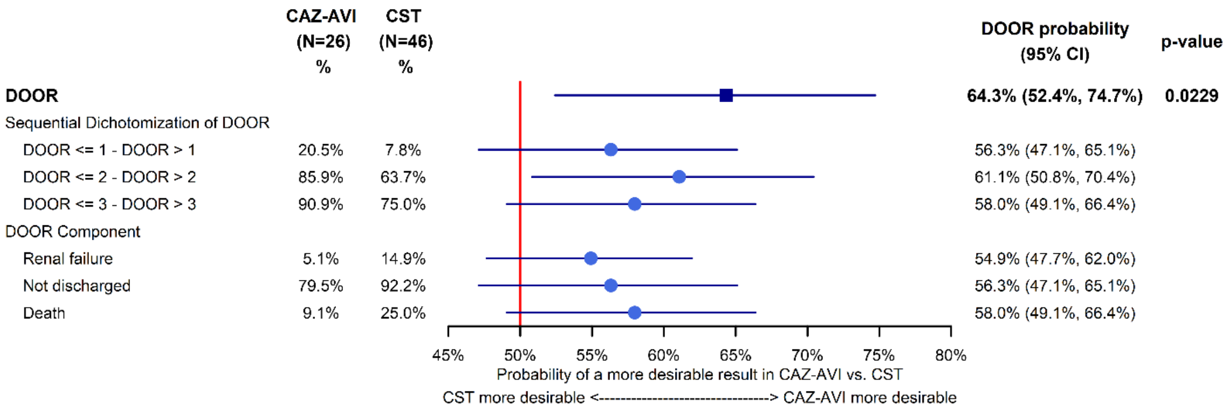

Figure A46. CRACKLE: The difference of DOOR outcome and respective components between the two intervention groups (adjusted)

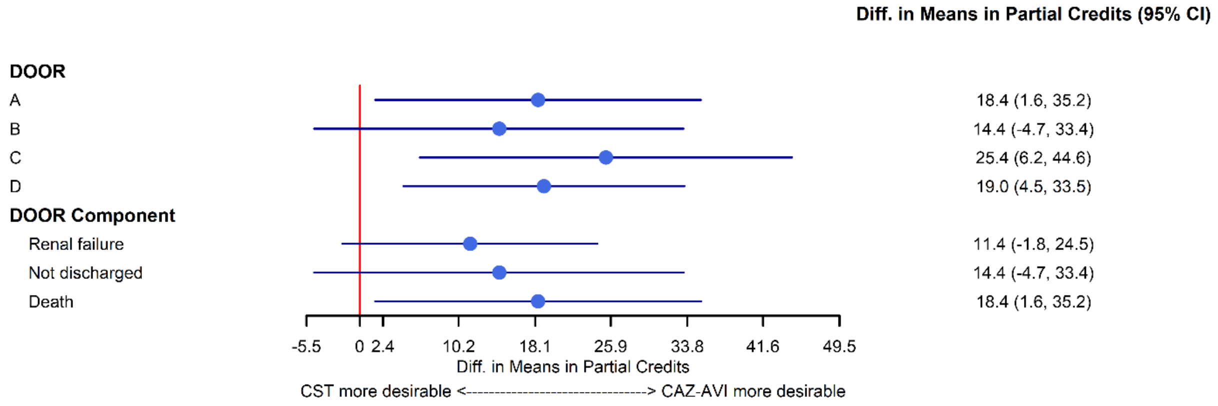

Figure A47. CRACKLE: Forest of difference in means of partial credit for four grading keys (unadjusted). For the DOOR component, the "no" category was given 100 full credits and the "yes" category was given zero credits.

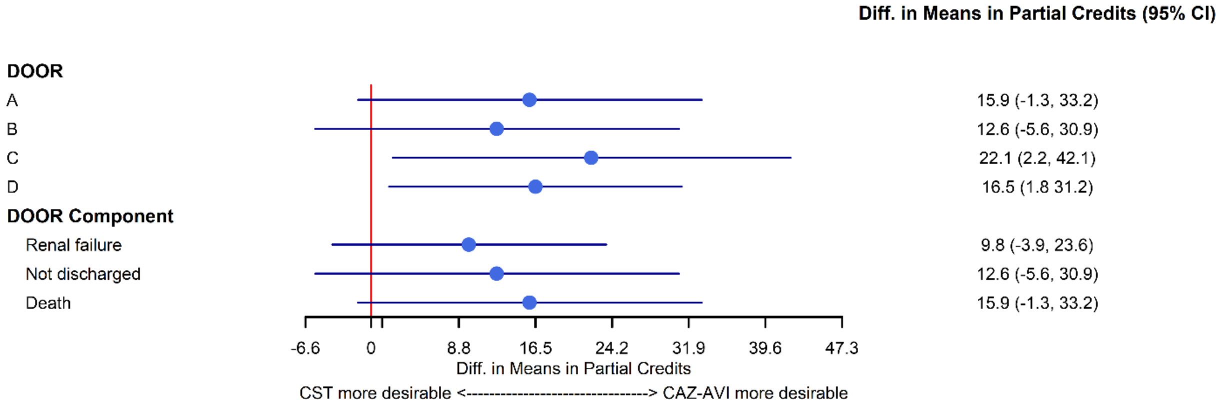

Figure A48. CRACKLE: Forest of difference in means of partial credit for four grading keys (adjusted). For the DOOR component, the "no" category was given 100 full credits and the "yes" category was given zero credits.

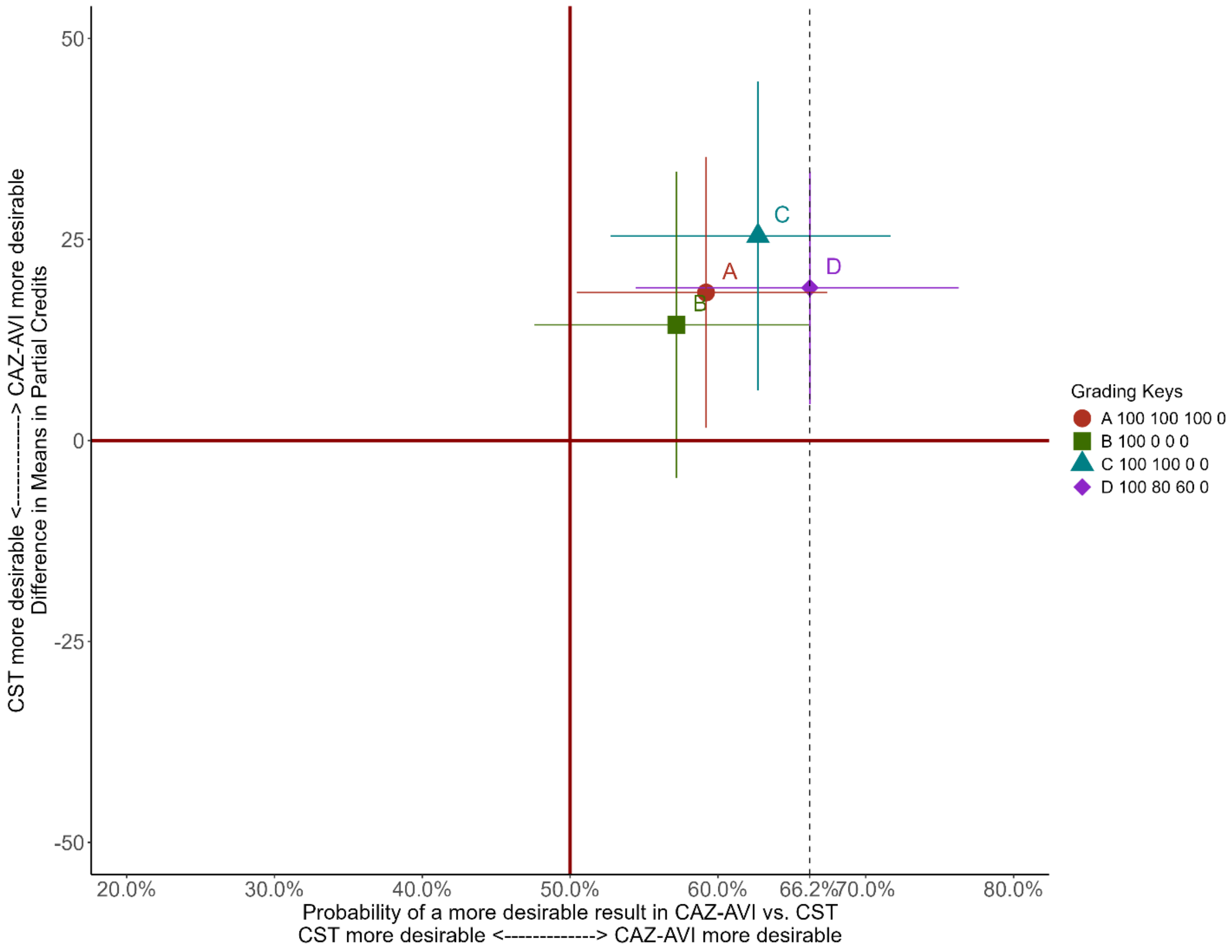

Figure A49. Plot of difference in means of partial credit vs. DOOR probability for four grading keys (unadjusted)

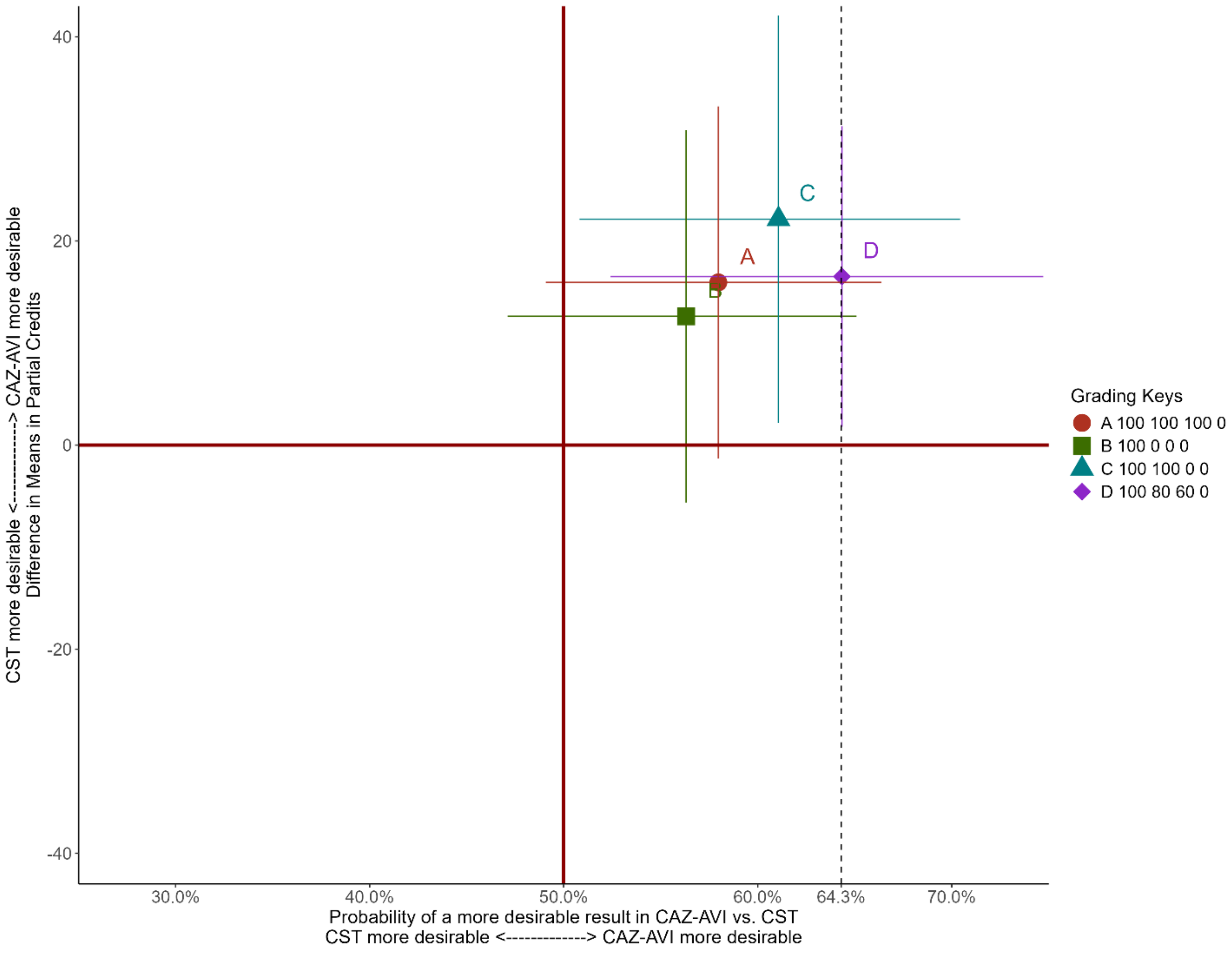

Figure A50. Plot of difference in means of partial credit vs. DOOR probability for four grading keys (adjusted)

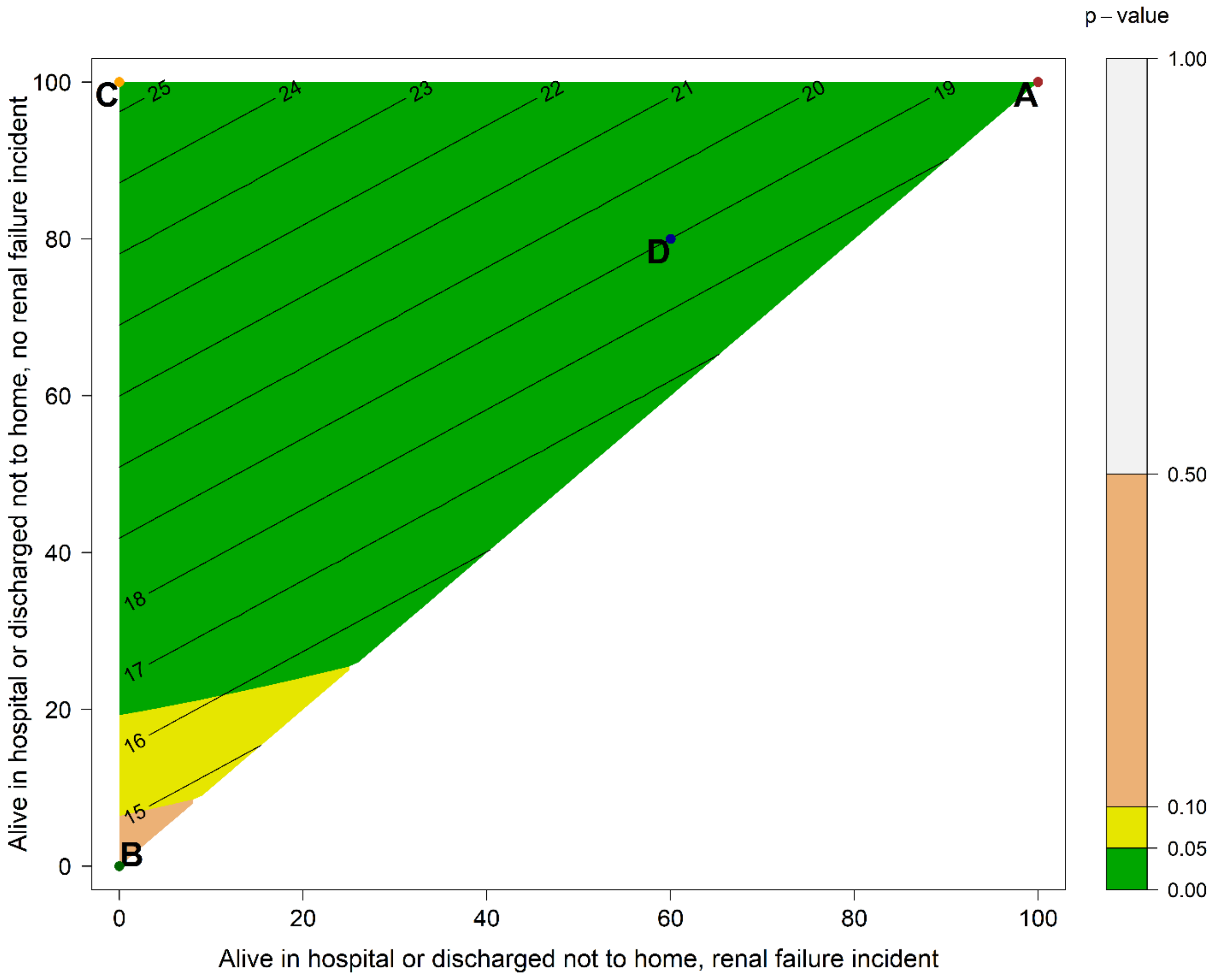

Figure A51. Contour plots of the between-group difference in means as the partial credit assigned to DOOR outcome level two (alive in hospital or discharged not to home, no renal failure incident) and level three (alive in hospital or discharged not to home, renal failure incident) vary (unadjusted). Green areas indicate grading key combinations that result in p-values less than 0.05; Positive numbers favor ceftazidime/avibactam and negative numbers favor colistin.

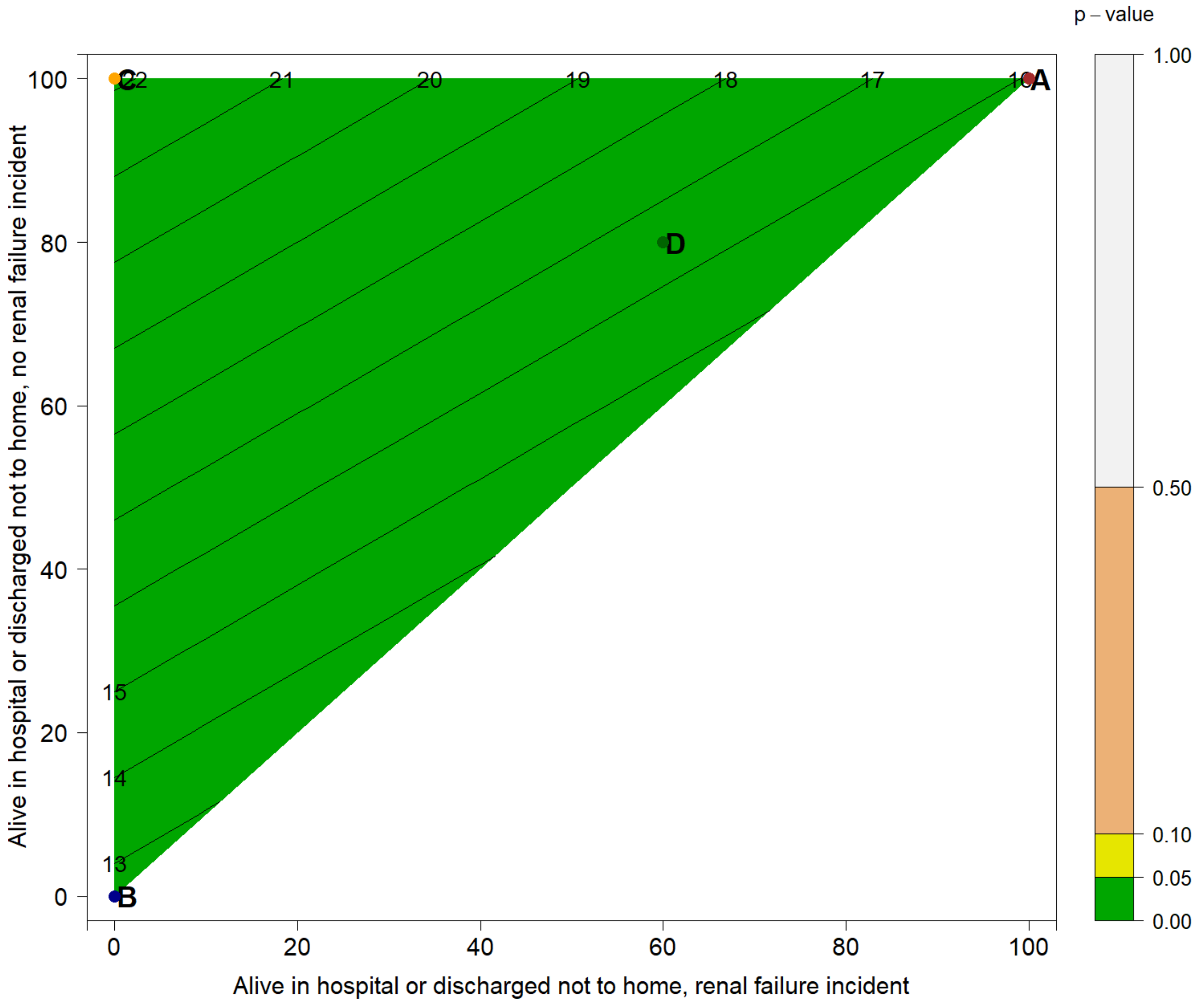

Figure A52. Contour plots of the between-group difference in means as the partial credit assigned to DOOR outcome level two (alive in hospital or discharged not to home, no renal failure incident) and level three (alive in hospital or discharged not to home, renal failure incident) vary (adjusted). Green areas indicate grading key combinations that result in p-values less than 0.05; Positive numbers favor ceftazidime/avibactam and negative numbers favor colistin.
